# Pharmacogenomics of intravenous immunoglobulin response in Kawasaki disease

**DOI:** 10.3389/fimmu.2023.1287094

**Published:** 2024-01-08

**Authors:** Sadeep Shrestha, Howard W. Wiener, Hidemi Kajimoto, Vinodh Srinivasasainagendra, Dolena Ledee, Sabrina Chowdhury, Jinhong Cui, Jake Y. Chen, Mikayla A Beckley, Luz A. Padilla, Nagib Dahdah, Hemant K. Tiwari, Michael A. Portman

**Affiliations:** ^1^ Department of Epidemiology, School of Public Health, University of Alabama at Birmingham, Birmingham, AL, United States; ^2^ Division of Cardiology, Seattle Children’s and University of Washington Department of Pediatrics, Seattle, WA, United States; ^3^ Department of Biostatistics, School of Public Health, University of Alabama at Birmingham, Birmingham, AL, United States; ^4^ Informatics Institute, School of Medicine, University of Alabama at Birmingham, Birmingham, AL, United States; ^5^ CHU Ste-Justine, Universite de Montreal, Montreal, QC, Canada

**Keywords:** Kawasaki disease, whole genome sequencing, IVIG refractory, ancestry, Pharmacaeconomics

## Abstract

**Introduction:**

Kawasaki disease (KD) is a diffuse vasculitis in children. Response to high dose intravenous gamma globulin (IVIG), the primary treatment, varies according to genetic background. We sought to identify genetic loci, which associate with treatment response using whole genome sequencing (WGS).

**Method:**

We performed WGS in 472 KD patients with 305 IVIG responders and 167 non-responders defined by AHA clinical criteria. We conducted logistic regression models to test additive genetic effect in the entire cohort and in four subgroups defined by ancestry information markers (Whites, African Americans, Asians, and Hispanics). We performed functional mapping and annotation using FUMA to examine genetic variants that are potentially involved IVIG non-response. Further, we conducted SNP-set [Sequence] Kernel Association Test (SKAT) for all rare and common variants.

**Results:**

Of the 43,288,336 SNPs (23,660,970 in intergenic regions, 16,764,594 in introns and 556,814 in the exons) identified, the top ten hits associated with IVIG non-response were in *FANK1, MAP2K3:KCNJ12, CA10, FRG1DP, CWH43* regions. When analyzed separately in ancestry-based racial subgroups, SNPs in several novel genes were associated. A total of 23 possible causal genes were pinpointed by positional and chromatin mapping. SKAT analysis demonstrated association in the entire *MANIA2, EDN1, SFMBT2*, and *PPP2R5E* genes and segments of CSMD2*, LINC01317, HIVEPI, HSP90AB1*, and *TTLL11* genes

**Conclusions:**

This WGS study identified multiple predominantly novel understudied genes associated with IVIG response. These data can serve to inform regarding pathogenesis of KD, as well as lay ground work for developing treatment response predictors.

## Introduction

KD is a life-threatening acute vasculitis that diffusely involves multiple organ systems in children and has a predilection for involvement of the coronary arteries (1, 2). The pathological walls of afflicted vessels show propensity for forming thrombosis and aneurysms. The diagnosis is made according to guidelines published by the American Academy of Pediatrics (AAP) and the American Heart Association (AHA) (1). The epidemiology of KD in the U.S still requires clarification. Current data suggest up to 7000 cases per year in the U.S. or 20 cases per 100,000 children (1, 3). The incidence of KD varies substantially with high incidence clusters in Eastern Asian countries such as Japan, approaching 300 per 100,000 children (4–6), with a similar incidence (220 per 100,000) in Japanese-Americans (7–9), Taiwan, and Korea, compared to Caucasian populations in the U.S with nearer to 20 cases per 100,000. Europe has an even lower incidence, illustrating the marked racial/ethnic heterogeneity for KD.

Primary treatment of acute KD with intravenous gamma globulin in addition to aspirin was instituted in the 1980s after multiple clinical trials showed efficacy and continued currently without much modification (10, 11). However, therapy fails in a significant number (13-23%) of children (12, 13). Patients deemed resistant to Intravenous Immunoglobulin (IVIG - * terms “IVIG resistant” and “IVIG refractory” are used interchangeably through literature) by AHA guidelines (persistent or recurrent fever) show substantially higher rates of eventually developing persistent coronary artery disease (11, 14–16). They require additional therapies (AHA recommended), which as of now still require validation for efficacy and include additional IVIG, tumor necrosis factor -alpha (TNF-α) antagonists, or steroids (1, 17). Ethnic or regional differences in treatment response are difficult to discern from literature due to practice variations and often unclear or divergent definitions for outcome parameters including IVIG resistance. Some studies show that children with Hispanic or African American ancestry demonstrate higher rates of IVIG resistance than comparable populations with European or East Asian ancestry (18, 19).

No practical or consistent biomarkers are currently available which can accurately predict risk for IVIG refractoriness in North American children with KD. Furthermore, the mechanisms for IVIG action in KD still require elucidation. The inability to predict IVIG treatment refractoriness serves as a major impediment to the progress and development of intensified therapy for KD patients. Currently available data indicate that KD treatment response, similar to susceptibility, depends on an individual patient’s genetic background (20–28). However, most of these studies investigating host genetic factors evaluated common variants, which may or may not offer relevance for treatment response for a rare disease like KD. Individual SNPs tests are still a useful tool for rare-variant (minor allele frequency (MAF) < 0.01) analysis if the sample sizes are large enough or the effects are very large; however, aggregation tests can evaluate cumulative effects of multiple genetic variants in a gene or region, increasing power. Further, studies have been impaired by 1) phenotyping lacking rigor, 2) use of genome-wide association studies often employing chips or arrays for detection of common variants rather than rare variants, 3) lack of clarity regarding the mechanism for IVIG anti-inflammation action in KD (necessary for guiding most pharmacogenomics studies) and leading to focus on gene candidates, which are impractical for clinical testing.

Our primary objective for this study was to identify genomic loci associated with IVIG response. We performed Whole Genome Sequencing (WGS) association analyses in a cohort of KD patients in a racially diverse North American population. Principal component analyses of ancestry information markers were incorporated to determine racial or ethnic diversity in treatment responses. We identified multiple loci associated with clinical IVIG refractoriness among a pediatric population of KD patients. Single nucleotide polymorphisms within these loci might serve as predictors for IVIG refractoriness, as well as inform regarding mechanisms of IVIG action.

## Materials and methods

The data that support the findings of this study are available from the corresponding author upon reasonable request.

### Study populations

This study was approved by each participating site institutional review board (IRB) and participant’s parents or legal guardians consented to all procedures and data collection. Clinical data and DNA samples were retained at a biorepository administered by M.A.P at Seattle Children’s Research Institute, and were obtained from enrolled participants in the United States and Canada. The biorepository at sequencing time contained samples from approximately 900 patients and is growing due to continued enrollment. The samples used for this study were collected between 2010 and 2019. Clinical data was confirmed by review of medical records prior to entry into the database. All patients included in this WGS qualified by meeting criteria for complete or incomplete KD diagnosis set by the AHA) and AAP (1) (included in **Supplementary Methods**), and then were treated with high dose IVIG infusion (2gm/kg) and aspirin (variable along AHA guidelines generally either moderate dose 30-50 mg/kg/day or high dose 80-100 mg/kg/day depending on practitioner (1), followed by low antiplatelet dose ASA (3-5 mg/kg/d) after fever resolved for 48 hours. Patients were excluded if they participated in a clinical trial, which might affect outcome or if they received glucocorticoids, immunosuppressives, or biologics prior to IVIG. They were assigned as IVIG responder or refractory according to AHA/AAP 2017 guidelines. Refractory was defined as having persistent or recurrent fever (> 38 C) more than 36 hours after completion of the IVIG infusion (1). We then drew from the IVIG responder pool (no recurrent or persistent fever) based on enrolment date, quantity, and quality of DNA with a target of near 1:1.5-2.0, non-responders versus responders. This resulted in 305 responders and 167 non-responders (see flow chart in **Supplementary Methods**).

#### Whole genome sequencing and variant calling

Genomic DNA was extracted from either blood or saliva using the Versagene DNA purification kit (Gentra Systems) and quantified using the PicoGreen assay for double-stranded DNA, adjusted to a final concentration of 100 ng/μL, and stored at −80°C in Tris-EDTA. DNA was checked for quality and PCR-free libraries were generated using the BGI DNBSEQ True PCR-Free platform (Beijing Genomics Institute; Guangdong, Shenzhen, China). At least 1.0 μg was obtained for each individual and used to create WGS library, which insert sizes 300–500 bp for paired-end libraries according to the BGI library preparation pipeline. Whole genome sequencing was performed on the MGISEQ-2000 instrument (Beijing Genomics Institute; Guangdong, Shenzhen, China) to generate 100 bp paired-end reads. The average sequencing depth was 30x per individual. Rigorous read mapping, variant calling, and quality recalibration for the genomic data were performed for each sample after passing data quality control.

Raw sequence data files were processed according to the Broad Institute’s Genome Analysis Tool Kit (GATK) best practices workflow for small germline variants. Briefly, FastQC data was used to check the quality of the raw reads. All reads that passed were aligned to the human reference genome (hg38) using Burrows-Wheeler aligner (BWA) v 0.7.17. Each sample had been run on four sequencing lanes so that each individual had four mapped files. Samtools version 1.12 was used to sort and index each mapped file and Picard-tools version 2.20.1 was used to merge the files into a single combined mapped file per sample. In preparation for variant calling, Picard-tools was also used to mark duplicate reads and base recalibration. Joint variant calling including insertions and deletions across all samples were called according to the GATK.

#### Quality control

Variant-level QC was performed using the Variant Quality Score Recalibration tool (VQSR) from the Genome Analysis Toolkit (GATK), using the recommended threshold of 99% sensitivity for the “true” variant. For sample-level QC, we made extensive use of X and Y chromosome data to confirm the gender, and we had four duplicates. Heterozygosity/Homozygosity ratios, transition/transversion (Ts/Tv) ratios, and missingness along with Hardy-Weinberg equilibrium test were also used for quality control.

Variant files were then annotated with ANNOVAR v. and the functional impact of the single nucleotide polymorphisms (SNPs) (i.e., synonymous, missense, frameshift, etc.) were evaluated using SIFT and PolyPhen software.

### Analysis

#### Whole genome sequencing association - single-variant analysis

Genetic kinship matrix was estimated with the *ibs* function. The kinship matrix was estimated based on all diallelic SNPs, considered as tagging SNPs that are not in high LD with each other. This was done to remove non-informative variants in the construction of the kinship matrix. Any associated top hits with rare variants SNPs in the primary analyses and HWE cut-off of *P* < 1.0E-05 were noted.

Unrelated SNPs (r^2^<0.2) throughout the genome with minor allele frequency of at least 2% were used to calculate principal components of ancestry using the principal components analysis (PCA) program from the Eigenstrat software package (29). Further, the values calculated by means of PCA were subjected to a discriminant analysis (30) by using the Discrim procedure in SAS version 9.4 (SAS Institute, Inc, Cary, NC) that categorized them to distinct ancestry background-based race.

PLINK 1.90 was used to perform an association analysis of individual SNPs based on an additive model. As described above, in a case control study design, logistic regression analyses were used to assess the association between each SNP and the odds of not responding to IVIG treatment. All models were adjusted for age at KD diagnosis, gender and three principal components for ancestry-based race (for population substructure) to estimate per-allele adjusted odds ratios (aOR) and 95% confidence intervals (95% CI). Additionally, ancestry-specific analyses assigned by PCA were also conducted for White, African-American, Asian, and Hispanic. Quantile-quantile (QQ) plots and Manhattan plots were produced with the qqman package in R. Regional association plots were constructed using Locuszoom. The color scheme for LD range was based on our own dataset since this was a case-only cohort (KD patients).

#### Functional annotation

To better understand the genetic mechanisms underlying IVIG non-response, Functional Mapping and Annotation (FUMA) v1.3.0 (31) was used to functionally map and annotate the genetic associations. SNP2GENE process annotated SNPs regarding their biological functions and mapped them to genes and GENE2FUNC annotated the mapped genes in biological contexts. SNPs with WGS association analysis *p* < 1.0  × 10^-6^ and all variants in *r* ^2^ ≥ 0.6 with them were prioritized to conduct Combined Annotation-Dependent Depletion (CADD) analysis (32), expression quantitative trait loci (eQTL) variant mapping (33), 3D chromatin interaction mapping (Hi-C) (34), annotation of enhancers (35), tissue-specific expression of genes identified via Hi-C and eQTL mapping (33). Positional mapping was used to map SNPs based on their physical position inside a gene using a 10 kb window. eQTL mapping was used to map SNPs to genes within 1 Mb (*cis*-eQTL) based on the evidence that the SNP was associated with the expression of that gene (false discovery rate ≤0.05).

### Gene-based analysis

Standard methods used to test for association with variants are underpowered for rare variants unless sample sizes or effect sizes are very large. Thus, we considered an alternative approach of assessing cumulative effects of multiple variants (specifically rare) in the gene region. We extracted gene borders from the National Center for Biotechnology Information Reference Sequence Database, and we included an additional window of 200 kb flanking on either side of each gene. We used SNP-set [Sequence] Kernel Association Test (SKAT) that aggregates the associations of rare variants and the phenotype (IVIG response) through a kernel matrix allowing SNP-SNP interactions (36). It is a non-burden test so we also used the Optimal Unified Test SKAT-O, which uses the data to adaptively select the best linear combination of the burden test and SKAT to maximize test power (37). Genes were prioritized based on the positional locations, e-QTL or chromatin interaction function of the associated SNPs. We also used all variants (rare and common) in both of these models (38). SKAT analyses were conducted with SNPs in the entire gene, but also at various segments of intronic and exonic regions.

## Results

We performed whole genome sequencing in 472 patients, who rigorously fulfilled American Heart Association criteria for complete (362) or incomplete (110) diagnosis and IVIG treatment response. Study participant demographics are shown in **Table 1**. Among the 472 patients, 305 (148 Whites, 83 Asian, 45 Hispanics and 29 African Americans) were IVIG responsive by AHA criteria, while 170 (103 Whites, 28 Asians, 26 Hispanics and 10 African Americans) exhibited recurrent or persistent fever deeming them nonresponsive. The median age of IVIG responders at diagnosis, was 32 months and non-responders was 34 months. The Principal Component plot (PC1 vs. PC2 vs. PC3) are shown in **Supplementary Figure 1**.

**Table 1:** Demographics of Kawasaki Disease Patients in the Study.

	IVIG Responders (N = 305)	IVIG Non-responders (N = 167)
Median (IQR] age (months	32 [25-41]	34 [27-42]
Gender Males Females	177 (48%) 128 (42%)	106 (63%) 61 (37%)
PCA-based Ancestry White Asian Black Hispanic	148 (48.5%) 83 (27%) 29 (9.5%) 45 (15%)	103 (62%) 28 (17%) 10 (6%) 26 (15%)

Among the 472 KD patients, 49,686,005 variants passed VQSR quality check of which 48,304,062 variants had calls for both reference and alternate allele variants in our study population, including 43,288,336 single nucleotide polymorphism **Supplementary Table 1A**). Majority of the SNPs were in the intergenic region (23,660,970) and intronic (16,764,594 SNPs). We also identified 556,814 SNPs in the exon, including 8,250 frameshift, 8 non-frameshift, 216,966 nonsynonymous, 1,312 stop-gain, 530 stop-loss, 161,094 synonymous and 2028 unknown function. SNPs in other parts of the genome are shown in **Supplementary Table 1B**. The average ts/tv ratio was 2.05 and the average GC content was 41.5% (ranging 40.0% - 44.1%) without any base bias (**Supplementary Table 2**). The average coverage depth was 28.07X. The 11 duplicates showed high SNP genotype concordance, with a kinship coefficient estimate, Φ > 0.497 between duplicates (which shows high concordance, max Φ = 0.5), but none of the participants in the study were related. The proportion of heterozygous vs. homozygous variants among all the samples is 1.39, consistent with the statistical expectation indicating good quality of the variant calls. In total, 19,531,404 of the variants were uniquely present in single patients.

### Variants significantly associated with IVIG refractoriness

**Figure 1:**
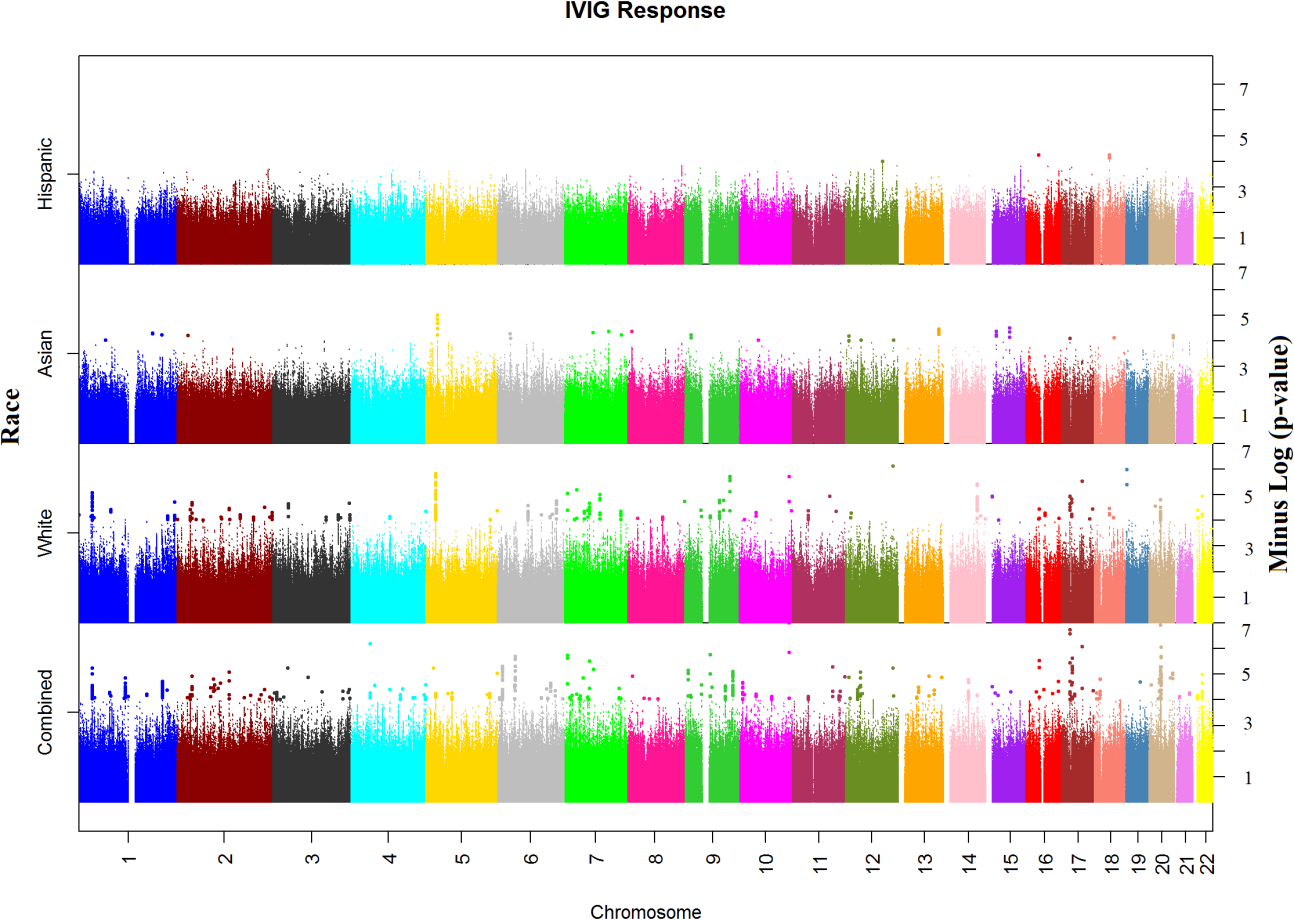
Manhattan plots of Whole Genome Sequencing (WGS) association analysis for IVIG refractory. Plots for Combined and Ancestry-specific Whites, Asians and Hispanics separately are shown.

In the WGS association analysis for entire study population (combined), the top significant signals for IVIG response were in Chromosomes 10, 17, and 20 (**Figure 1**). We considered all these top hits as novel as they have not been previously associated with KD or IVIG treatment responses. The top ten hits reside in Chromosome 10 (rs77740910 p < 4.96E-08) and rs73368612 (p < 1.72E-06) both in intronic region of *FANK1*; chromosome 17 (rs113336767 p < 4.11E-07), rs75317727 p < 5.80E-07) in intergenic region of *MAP2K3:KCNJ12* and rs74255119 p < 1.01E-06) in intronic region of *CA10*; in chromosome 20 (C20_29079715 p < 2.45E-07) upstream of *FRG1DP* and C20_30491365 p < 1.84E-06) in the intragenic region of *FRG1BP : DEFB115*; and C4_49554582 (p < 7.74E-07) in intergenic region of CWH43 in chromosome 4, C7_6981323 1.86E-06 in intergenic region of CCZ1B:MIR3683 in chromosome 7, and C9_639065872.07E-06 in intergenic region of FRG1JP : FLJ43315 in chromosomes 9. Other top SNPs and gene/gene regions (including *CSMD2, MAN1A2, PPP2R5A, LINC01317, LINC02211:CDH9, HIVEPI : EDN1, HSP90AB1, TTLL11, SFMBT2, PPP2RSE and GSTTP2*) in combined or individual ancestry are listed in **Supplementary Table 3**).

**Figure 2:**
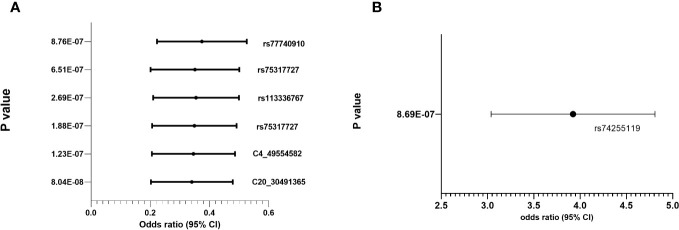
Genotype distribution and OR (95% CI) of the allelic additive models for minor alleles for the top hits in the chromosomal regions for IVIG refractory **(A)** Protective Alleles **(B)** Risk Allele.

Regional association plots in genes with top hits in combined population, Whites, Asians and African-Americans (no region with statistically significant SNP(s) in Hispanic) are shown in **Supplementary Figures 2A–D**. The minor allele frequencies of the lead SNPs in our population (both IVIG responders and non-responders and the 5 major continent populations (East-Asia (EAS), Americas (AMR), Africa (AFR), Europe (EUR) and South Asia (SAS)) in 1000 genome are shown in **Supplementary Table 3**. The association results using allelic additive model (OR and 95% CI) for the top hits are shown in **Figure 2** (results for other associated SNPs are shown in **Supplementary Figure 3** (genotype distribution) and **Supplementary Figures 4A, B** (Odd ratio and 95% CI from association study).

Manhattan plot of SNP analysis for each ancestry (White, Asian and Hispanic) are shown also in **Figure 1** and the related QQ-plots for all combined and individuals ancestry analysis in **Supplementary Figures 5A–D**. When stratified by ancestry, only 2 top hit SNPs (C4_49554582 near *CWH43* gene in chromosome 4 and rs324130 in the intragenic region between *SLC29A1* and *HSP90AB1* genes in chromosome 6) showed statistical association (p<0.05) in all four ancestral group (**Supplementary Table 3**). For most SNPs, association was statistically significant in only one ancestral population that also drove the association in the combined analyses. When examining the top hits in the combined population, several of these SNPs showed statistical association in 2 or 3 ancestral population and the associations in the same direction (beta values) despite small sample sizes (**Supplementary Table 3**), specifically in African American and Hispanic populations.

### Functional mapping and annotation for genes associated with IVIG response

**Figure 3:**
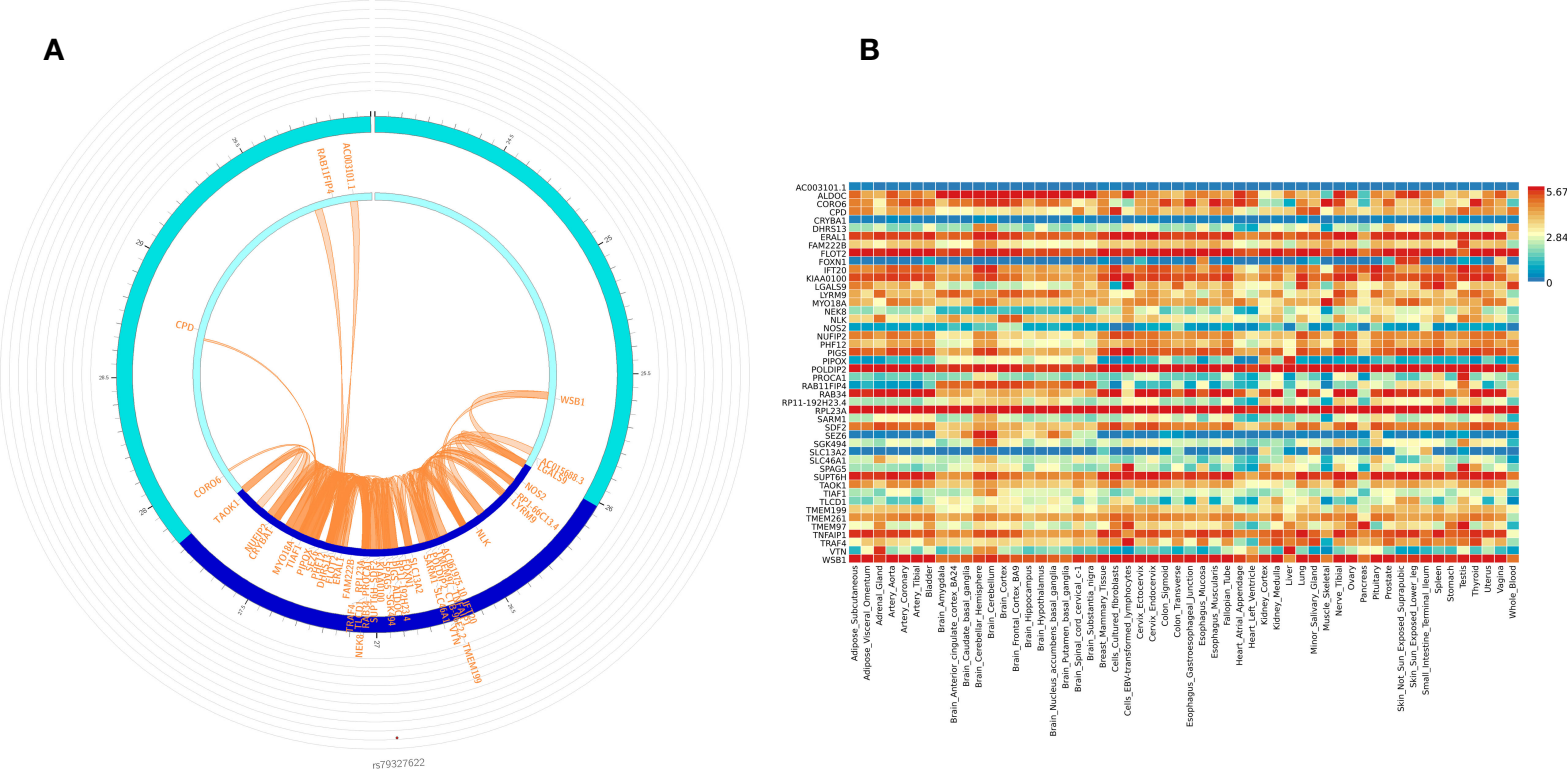
Functionally significant genes: **(A)** 3D chromatin interaction (Hi-C) and eQTL analysis for genomic risk locus in chromosome 17. The most outer layer displays the rsID of the top SNPs in each risk locus and other SNPs with P < 0.05 from Manhattan plot in the genomic risk loci. SNPs in genomic risk loci are color-coded as a function of their maximum r^2^ to the one of the independent significant SNPs in the locus, as follows: red (r^2^ > 0.8), orange (r^2^ > 0.6), green (r^2^ > 0.4) and blue (r^2^ > 0.2). SNPs that are not in LD with any of the independent significant SNPs (with r^2^ ≤ 0.2) are grey. The second layer is the chromosome ring and the Genomic risk loci are highlighted in blue. Only mapped genes by either chromatin interaction and/or eQTLs (conditional on user-defined parameters) are displayed. If the gene is mapped only by chromatin interactions or only by eQTLs, it is colored orange or green, respectively. When both map the gene, it is colored red. The third layer shows the genomic coordinate that aligns with the position of the SNPS and the genes **(B)** Tissue-specific expression of *all 49 genes*. Colors reflect average expression (log2 transformed) from highest (red) to lowest/absent (blue).

We used FUMA to prioritize genes and gene regions (p < 3.10E-06) from all participant WGS association analysis for IVIG non-response. Within FUMA 18 SNPs (4 independent significant SNPs) with 2 lead SNPs (rs12378393 in chromosome 9 and rs79327622 in chromosome 17) were identified using the SNP2GENE function (**Supplementary Table 5A**). For these 18 SNPs, 9 intron variants, 4 intergenic variants, one upstream, one downstream, one on 5’UTR, one on ncRNA intron, and unknown variant, CADD analysis, a scoring system for deleteriousness of genetic variants, identified one SNP rs199862288 (in intron of *TAOK1* gene) with CADD 14.68, which places it among the top 10% (CADD > 10) to 1% (CADD > 20) of most deleterious mutations in the genome. FUMA mapped 50 genes (**Supplementary Table 3**) to these 2 genomic risk loci (**Supplementary Table 5B**). 3D chromatin interaction mapping (Hi-C) analysis showed chromatin interaction between rs79327622 and forty-nine genes on chromosome 17 (**Figure 3A**). **Figure 3B** shows the expression of these genes in various tissues. While eQTL information was not available for the SNPs, 23 genes were prioritized by both positional and chromatin mapping strategies.

### SKAT analysis

Aggregate SNPs (both rare and common) analyses using various models of SKAT demonstrated association of rare variants, functional variants and all variants in the entire *MANIA2, EDN1, SFMBT2*, and *PPP2R5E* genes and segments of *CSMD2, LINC01317, HIVEPI, HSP90AB1*, and *TTLL11* genes (**Supplementary Table 4**).

## Discussion

We leveraged high-coverage WGS data in a cohort of racially diverse Kawasaki disease (KD) patients in North America. We assembled a catalogue of genomic variants in patients with KD, a rare pediatric disease, and conducted the first WGS pharmacogenomics study that revealed novel SNPs and genes involved in IVIG treatment response. These novel genetic markers might be used eventually to rapidly identify high-risk KD patients and initiate aggressive therapy. The interactions of these genes also inform the potential mechanism of IVIG anti-inflammatory action and identify potential therapeutic targets to alleviate adverse outcomes including development of coronary artery disease (CAD). Consistent with other studies of complex diseases, significant associations of noncoding variants vastly outnumber those with coding variation (39). Additionally, we found no highly significant associations among the many genes previously identified as potential determinants of IVIG response in KD.

Interestingly, top hits (intronic rs10473594 in *CDH12* (chr 5), intragenic rs7579420 in *LOC728597|LOC727982* (chr 2), and intronic rs3777914 and rs1883137 in *TRAF3IP2* (chr 6)) from our previous family based Transmission Disequilibrium Test TDT analysis in Whites (40) were also statistically associated with IVIG non-response among Whites in this population-based study (**Supplementary Table 6A**). However, SKAT analysis of different regions of these previously associated genes (*CASP3, ABCC4, IL6, IL4R and NFATC2*) showed significant associations and warrants further investigation in the future (**Supplementary Table 6B**).

In line of locus heterogeneity, there were other SNPs in several of these genes that were found to be significant (although not the top hits) that require further investigation. In contrast, we found the associations of several other novel SNPs in different genes with the two outcomes that have biological plausibility. The most significant SNPS was in the *Fibronectin Type III and Ankyrin Repeat Domains 1* (*FANK1*) gene. This gene is understudied but has been shown to trigger the activator protein 1 (AP-1) pathway in a Jun activation domain-binding protein 1 (Jab1) dependent manner and thereby suppresses cell apoptosis (41). Thus, mutations in *FANK1* may dysregulate AP-1 signaling and impair cell death, increasing an individual’s risk for inflammatory processes and disease. Heat shock proteins (HSPs) are a large group of chaperones that stabilize proteins. HSPs respond to various cellular stressors and are involved in protein transport across membranes, including the mitochondrial membrane (42). Of the exonic SNPs, rs13296 in chromosome 6 was the most statistically significant SNP p < 7.49E-06) associated with IVIG non-response and is located in *Heat Shock Protein 90 kDA Alpha, Class B, Member* 1 (*HSP90AB1*). It is a member of the HSP family that depends on ATP (42). HSP90s alter immune processes, such as activating lymphocytes (43). Highly expressed HSP90AB1 and HSP90B1 negatively correlated with the infiltration of CD4+ T cells in a study exploring immune infiltration in breast cancer (44). Further, HSP90s act as immunogens, serving as a “danger signal” to stimulate effector T cells (45). Thus, overexpression of *HSP90AB1* may exaggerate the activation of immune cells and lead to a hyper inflammatory response. Interestingly, a polymorphism at rs13296 within the exome is associated with SLE susceptibility in a Chinese cohort (46).

Several mechanisms for the anti-inflammatory actions of IVIG have been proposed including inhibition of pathogenic auto-antibodies, enhanced IgG clearance, complement modulation, altered function of macrophage/dendritic cells, suppression of pathogenic cytokines/induction of anti-inflammatory cytokines, Fc gamma receptors (FcGR) blockade, and neutralization of B- and T-cell function (47, 48). At present it remains unknown which of these mechanisms best explain the therapeutic effects of IVIG in KD. Our group has also worked in detail with genetic variants in *FcGR* gene family to determine their role in IVIG response (20–22, 49). However, *FcGR* genes comprise a fairly complex gene region including duplications, overlaps, and alternative splicings, and therefore require very rigorous sequencing and analyses (49–52); and may not be practical for rapid clinical informative testing. However, *Mannosidase Alpha Class 1A Member 2* (*MAN1A2*) is a protein coding gene. Alpha 1,2-mannosidases are essential for the formation of N-glycans on mammalian glycoproteins (53). Post translational modifications like membrane protein folding depend on N-linked glycosylation (54). Glycans have several biological functions, including activating the innate immune system and serving as pathogenic trigger for autoimmune diseases (55). Patients with rheumatoid arthritis exhibit altered N-glycosylation structures of immunoglobulin Gs (IgGs) (56). Site-specific changes in N-glycosylation impair antigen binding fragment (Fab) of IgGs, as well as the Fc portion (57). Thus, expression of dysfunctional glycoproteins on the cell surface and in extracellular compartments may impair signaling pathways that promote immune-cell differentiation. In particular, alterations in glycosylation of cell receptors for immunoglobulins, such as DC-SIGN could modify the anti-inflammatory response to IVIG (58).

Besides the 2 genomic regions chromosomes 9 and 17 from FUMA analyses, there were several genes that showed regional significant association in several different genes (**Supplementary Table 4**.) There were several SNPS and regions in SKAT analysis in the *CUB and Sushi Domain-Containing Protein 2* (*CSMD2*) gene that appeared to be associated with IVIG non-response. This gene encodes a protein likely involved in controlling the complement cascade of the immune response (59, 60). A study found that the development and maintenance of dendrites and dendritic spines in the brain depends on CSMD2 (61) Downregulation of CSMD2 is associated with a poor prognosis in colorectal cancer (62). Further, in many cancer types, CSMD2 expression is upregulated and is associated with a higher stage and poor prognosis. Further, CSMD2 was negatively correlated with MHC-I molecules and positively correlated with MHC-II molecules. MHC-I molecules present endogenous antigens and activate cytotoxic CD8+ T-cells. MHC-II molecules present exogenous antigenic peptides to CD4+ T-cells, which activate CD4+ helper T-cells. CSMD2 expression was also negatively correlated with infiltration of anti-tumor immune cells, including macrophages and natural killer cells, and was positively associated with immune evasion (63). Thus, for patients with Kawasaki disease, a mutation altering CSMD2 may promote a hyper inflammatory response.

Several SNPs in EDN1 gene were associated with IVIG response and the gene burden test using SKAT also indicated association with EDN1 gene. *EDN1* alters physiological processes through transmembrane G-protein coupled receptors and is synthesized in a variety of cells, including endothelial cells, macrophages, and cardiomyocytes (64). As a vasoconstrictor, *EDN1* induces platelet aggregation and increases expression of leukocyte adhesion molecules, which plays an important role in vascular dysfunction and inflammatory processes (65). Data suggest involvement of *EDN1* in various infectious disorders (i.e., sepsis and viral or bacterial pneumonia), as well as KD (64, 66, 67). During the acute stage of KD, especially for those with coronary artery dilation, increased plasma concentrations of *EDN1* are present compared with controls (67). Thus, patients with IVIG non-responsiveness may have an exaggerated immune response and express *EDN1* at higher levels than responders. The risk locus (rs9349152) in the *EDN1* gene region is associated with eQTL of *PHACTR1* gene. Variants in the *PHACTR1/EDN1* gene locus have been associated with vasculopathies including spontaneous coronary artery dissection (SCAD), fibromuscular dysplasia, and cervical artery dissection (68, 69).

Our ancestry-specific analysis was limited by sample-size, specifically in African American and Hispanic subgroups. However, several interesting observations were noted. First, locus and allelic heterogeneity appear to occur across the four ancestral population in our study. For instance, the top SNP in Whites (rs117408018 in the intron of *CABP1* gene) was not significant in the other ancestral population. The MAF was <0.05 in both cases and controls in all other ancestries and similar to the 1000 Genome cohorts. While the White KD controls had a MAF of 8% and the 1000 Genome of 14%, the KD non-responders had significantly higher frequency (23%). Some SNPs significant in one ancestral population are also absent or rare in other ancestral population (e.g. rs77721353). We have observed similar discrepancies in our previous studies in FcGR genes (22). Second, most of the SNPs and genes (including the top hits in *FANK1* and *FRG1DP*) indicated in our study are novel and understudied. Several SNPs did not have frequency documented in the 1000 Genome and FUMA indicated that there was limited functional information available.

In summary, we have identified novel gene loci, predominantly in non-exonic regions, which associate with IVIG treatment response in KD. Although WGS could not be performed in a timely manner for prediction of IVIG treatment response, this study could further identify a select panel genetic loci, which could serve as risk predictors, and be amenable to other more rapid type analyses similar to many diagnostic panels used today.

## Data availability statement

The data have been deposited with links to BioProject accession number PRJNA1055092 in the NCBI BioProject database (https://www.ncbi.nlm.nih.gov/bioproject/).

## Ethics statement

The parent cohort/study and this genomic study conformed to the procedures for informed consent (parental permission) approved by institutional review boards at all sponsoring organizations and to human-experimentation guidelines set forth by the United States Department of Health and Human Services. The studies were conducted in accordance with the local legislation and institutional requirements. Written informed consent for participation in this study was provided by the participants’ legal guardians/next of kin.

## Author contributions

SS: Conceptualization, Formal analysis, Funding acquisition, Investigation, Methodology, Resources, Supervision, Validation, Visualization, Writing – original draft. HW: Data curation, Formal analysis, Software, Writing – review & editing. HK: Data curation, Writing – review & editing. VS: Data curation, Formal analysis, Visualization, Writing – review & editing. DL: Data curation, Project administration, Writing – review & editing. SC: Data curation, Visualization, Writing – review & editing. JC: Data curation, Formal analysis, Writing – review & editing. JYC: Supervision, Validation, Writing – review & editing. MB: Data curation, Project administration, Writing – review & editing. LP: Data curation, Methodology, Project administration, Writing – review & editing. ND: Investigation, Methodology, Validation, Writing – review & editing. HT: Methodology, Software, Supervision, Validation, Writing – review & editing. MP: Conceptualization, Funding acquisition, Investigation, Methodology, Resources, Supervision, Validation, Visualization, Writing – review & editing.

## Supplementary material

Supplementary Figure 1 Principal Component Analysis Plot (PC1 vs PC2 vs PC3) for ancestry inference.

Supplementary Figure 2
**(A–D)** LocusZoom plot of top hit SNP association with IVIG refractory in combined population and by ancestry (Whites, Blacks, Asians). P-values (additive genetic model using logistic regression adjusted for age, gender, PC 1-3 for genetic ancestry) on the-log 10 scale are plotted on the left vertical axis and the chromosomal position is plotted along the horizontal axis along with the gene names and size of flanking region. SNPs are colored based on their pairwise correlation (r2) within our study population with the labeled top SNP, which has the smallest P value in the region (diamond purple). The right vertical axis indicates the regional recombination rate (cM/Mb), estimated from 1000 Genomes (EUR), which is overlaid in light blue. Arrows on the horizontal blue lines show the direction of transcription, and rectangles are exons.

Supplementary Figure 3 Genotype distribution between KD patients who respond to IVIG treatment and those who are refractory. Results for the top hits based on smallest p-value.

Supplementary Figure 4
**(A, B)** Associations of individual SNPs with IVIG refractoriness. Odds Ratio and (95% CI) for being a non-responder, based on the additive genetic model (for individual SNPs) using logistic regression adjusted for age, gender, PC 1-3 for genetic ancestry. Results for the top hits based on smallest p-value and **(A)** Susceptible Loci (OR>1) and **(B)** Protective Loci (OR<1).

Supplementary Figure 5
**(A–H)** Quantile–quantile (QQ) plots of observed versus expected *P* values for Whole Genome Sequencing (WGS) association analysis for IVIG refractory (corrected for genomic inflation (*λ)*. Plots for Combined and Ancestry-specific Whites, Asians and Hispanics separately are shown.

Supplementary Table 1
**(A)** Location of all SNPs with both reference and alternate variants observed in KD participants. **(B)** Functional SNPs observed in KD Participants.

Supplementary Table 2 Number of SNPs, Private SNPs and transition/transversion (Ts/Tv) ratio and mean depth coverage in each KD participant.

Supplementary Table 3 Minor Allele Frequency, SNP Characteristics in the Study Population and Reference 1000 Genome Populations and association with IVIG response in additive genetic model (beta and p-values).

Supplementary Table 4 SNP-set [Sequence] Kernel Association Test (SKAT) analysis (SKAT, SKATO, RareComm, RareCommoO)^1^ of gene prioritized from Whole Genome Sequencing Analysis (Combined ancestry) SKAT for Entire gene and sub-regions defined by Exons (E) and Introns (I) (start and end of genomic locations) (SKAT = using all SNPS in the region; SKAT = Optimal Unified Test (SKAT-O) using all SNPs in the region; RareComm = SKAT using only rare SNPS in the region; RareCommO = Optimal Unified Test (SKAT-O) using rare SNPs in the region).

Supplementary Table 5
**(A)** SNPs from WGS association analysis for IVIG refractory used in positional and functional (eQTL and chromatin interactions) mapping; **(B)** Prioritized genes identified from WGS association analysis for IVIG refractory by positional and functional (eQTL and chromatin interactions) mapping.

Supplementary Table 6
**(A)** Combined and Ancestry-specific Analysis results for SNPs in genes previously associated with IVIG response; **(B)** SKAT analysis of genes previously associated with IVIG response.

## References

[1] McCrindle BW Rowley AH Newburger JW Burns JC Bolger AF Gewitz M. Diagnosis, treatment, and long-term management of kawasaki disease: A scientific statement for health professionals from the American heart association. Circulation (2017) 135(17):e927–e99. doi: 10.1161/CIR.0000000000000484 28356445

[2] Orenstein JM Shulman ST Fox LM Baker SC Takahashi M Bhatti TR. Three linked vasculopathic processes characterize Kawasaki disease: a light and transmission electron microscopic study. PloS One (2012) 7(6):e38998. doi: 10.1371/journal.pone.0038998 22723916 PMC3377625

[3] Elakabawi K Lin J Jiao F Guo N Yuan Z. Kawasaki disease: global burden and genetic background. Cardiol Res (2020) 11(1):9–14. doi: 10.14740/cr993 32095191 PMC7011927

[4] Newburger JW Taubert KA Shulman ST Rowley AH Gewitz MH Takahashi M. Summary and abstracts of the seventh international Kawasaki disease symposium: December 4-7, 2001, Hakone, Japan. Pediatr Res (2003) 53(1):153–7. doi: 10.1203/00006450-200301000-00026 12508096

[5] Nakamura Y Yashiro M Uehara R Sadakane A Tsuboi S Aoyama Y. Epidemiologic features of Kawasaki disease in Japan: results of the 2009-2010 nationwide survey. J Epidemiol / Japan Epidemiological Assoc (2012) 22(3):216–21. doi: 10.2188/jea.JE20110126 PMC3798622 22447211

[6] Pilania RK Singh S. Kawasaki Disease. In: Cimaz R, editors. Periodic and Non-Periodic Fevers. Rare Disease Springer 2020. pp. 45–63. doi: 10.1007/978-3-030-19055-2_4

[7] Dean AG Melish ME Hicks R Palumbo NE. An epidemic of Kawasaki syndrome in Hawaii. J pediatrics. (1982) 100(4):552–7. doi: 10.1016/S0022-3476(82)80751-8 PMC7131104 7062202

[8] Burns JC Cayan DR Tong G Bainto EV Turner CL Shike H. Seasonality and temporal clustering of Kawasaki syndrome. Epidemiology (2005) 16(2):220–5. doi: 10.1097/01.ede.0000152901.06689.d4 PMC2894624 15703537

[9] Holman RC Christensen KY Belay ED Steiner CA Effler PV Miyamura J. Racial/ethnic differences in the incidence of Kawasaki syndrome among children in Hawaii. Hawaii Med J (2010) 69(8):194–7. PMC3118023 20845285

[10] Furusho K Kamiya T Nakano H Kiyosawa N Shinomiya K Hayashidera T. High-dose intravenous gammaglobulin for Kawasaki disease. Lancet (1984) 2(8411):1055–8. doi: 10.1016/S0140-6736(84)91504-6 6209513

[11] Newburger JW Takahashi M Burns JC Beiser AS Chung KJ Duffy CE. The treatment of Kawasaki syndrome with intravenous gamma globulin. N Engl J Med (1986) 315(6):341–7. doi: 10.1056/NEJM198608073150601 2426590

[12] Burns JC Capparelli EV Brown JA Newburger JW Glode MP. Intravenous gamma-globulin treatment and retreatment in Kawasaki disease. US/Canadian Kawasaki Syndrome Study Group. Pediatr Infect Dis J (1998) 17(12):1144–8. doi: 10.1097/00006454-199812000-00009 9877364

[13] Wallace CA French JW Kahn SJ Sherry DD. Initial intravenous gammaglobulin treatment failure in Kawasaki disease. Pediatrics (2000) 105(6):E78. doi: 10.1542/peds.105.6.e78 10835091

[14] Burns JC Glode MP. Kawasaki syndrome. Lancet (2004) 364(9433):533–44. doi: 10.1016/S0140-6736(04)16814-1 15302199

[15] Newburger JW Takahashi M Beiser AS Burns JC Bastian J Chung KJ. A single intravenous infusion of gamma globulin as compared with four infusions in the treatment of acute Kawasaki syndrome. N Engl J Med (1991) 324(23):1633–9. doi: 10.1056/NEJM199106063242305 1709446

[16] Jaggi P Wang W Dvorchik I Printz B Berry E Kovalchin JP. Patterns of fever in children after primary treatment for Kawasaki disease. Pediatr Infect Dis J (2015) 34(12):1315–8. doi: 10.1097/INF.0000000000000891 PMC4718845 26353031

[17] Burns JC Best BM Mejias A Mahony L Fixler DE Jafri HS. Infliximab treatment of intravenous immunoglobulin-resistant Kawasaki disease. J pediatrics. (2008) 153(6):833–8. doi: 10.1016/j.jpeds.2008.06.011 PMC2856847 18672254

[18] Padilla LA Collins JL Idigo AJ Lau Y Portman MA Shrestha S. Kawasaki disease and clinical outcome disparities among black children. J pediatrics. (2021) 229:54–60 e2. doi: 10.1016/j.jpeds.2020.09.052 PMC7513890 32980379

[19] Portman MA Dahdah NS Slee A Olson AK Choueiter NF Soriano BD. Etanercept with IVIg for acute Kawasaki disease: A randomized controlled trial. Pediatrics (2019) 143(6). doi: 10.1542/peds.2018-3675 PMC6564061 31048415

[20] Makowsky R Wiener HW Ptacek TS Silva M Shendre A Edberg JC. FcgammaR gene copy number in Kawasaki disease and intravenous immunoglobulin treatment response. Pharmacogenet Genomics (2013) 23(9):455–62. doi: 10.1097/FPC.0b013e328363686e PMC4400828 23778324

[21] Shrestha S Wiener H Shendre A Kaslow RA Wu J Olson A. Role of activating FcgammaR gene polymorphisms in Kawasaki disease susceptibility and intravenous immunoglobulin response. Circ Cardiovasc Genet (2012) 5(3):309–16. doi: 10.1161/CIRCGENETICS.111.962464 PMC3444514 22565545

[22] Shrestha S Wiener HW Olson AK Edberg JC Bowles NE Patel H. Functional FCGR2B gene variants influence intravenous immunoglobulin response in patients with Kawasaki disease. J Allergy Clin Immunol (2011) 128(3):677–80. doi: 10.1016/j.jaci.2011.04.027 PMC3444515 21601260

[23] Wu SF Chang JS Wan L Tsai CH Tsai FJ. Association of IL-1Ra gene polymorphism, but no association of IL-1beta and IL-4 gene polymorphisms, with Kawasaki disease. J Clin Lab Anal (2005) 19(3):99–102. doi: 10.1002/jcla.20059 15900570 PMC6807836

[24] Minami T Suzuki H Takeuchi T Uemura S Sugatani J Yoshikawa N. A polymorphism in plasma platelet-activating factor acetylhydrolase is involved in resistance to immunoglobulin treatment in Kawasaki disease. J pediatrics. (2005) 147(1):78–83. doi: 10.1016/j.jpeds.2005.03.037 16027700

[25] Burns JC Shimizu C Shike H Newburger JW Sundel RP Baker AL. Family-based association analysis implicates IL-4 in susceptibility to Kawasaki disease. Genes Immun (2005) 6(5):438–44. doi: 10.1038/sj.gene.6364225 PMC2911125 15889128

[26] Burns JC Shimizu C Gonzalez E Kulkarni H Patel S Shike H. Genetic variations in the receptor-ligand pair CCR5 and CCL3L1 are important determinants of susceptibility to Kawasaki disease. J Infect Dis (2005) 192(2):344–9. doi: 10.1086/430953 PMC2894631 15962231

[27] Khor CC Davila S Breunis WB Lee YC Shimizu C Wright VJ. Genome-wide association study identifies FCGR2A as a susceptibility locus for Kawasaki disease. Nat Genet (2011) 43(12):1241–6. doi: 10.1038/ng.981 22081228

[28] Onouchi Y Ozaki K Burns JC Shimizu C Terai M Hamada H. A genome-wide association study identifies three new risk loci for Kawasaki disease. Nat Genet (2012) 44(5):517–21. doi: 10.1038/ng.2220 22446962

[29] Price AL Patterson NJ Plenge RM Weinblatt ME Shadick NA Reich D. Principal components analysis corrects for stratification in genome-wide association studies. Nat Genet (2006) 38(8):904–9. doi: 10.1038/ng1847 16862161

[30] CR R. Linear statistical inference. New York: John Wiley & Sons (1973).

[31] Watanabe K Taskesen E van Bochoven A Posthuma D. Functional mapping and annotation of genetic associations with FUMA. Nat Commun (2017) 8(1):1826. doi: 10.1038/s41467-017-01261-5 29184056 PMC5705698

[32] Kircher M Witten DM Jain P O'Roak BJ Cooper GM Shendure J. A general framework for estimating the relative pathogenicity of human genetic variants. Nat Genet (2014) 46(3):310–5. doi: 10.1038/ng.2892 PMC3992975 24487276

[33] Consortium GT. Human genomics. The Genotype-Tissue Expression (GTEx) pilot analysis: multitissue gene regulation in humans. Science (2015) 348(6235):648–60. doi: 10.1126/science.1262110 PMC4547484 25954001

[34] van Berkum NL Lieberman-Aiden E Williams L Imakaev M Gnirke A Mirny LA. Hi-C: a method to study the three-dimensional architecture of genomes. J Vis Exp (2010) 39). doi: 10.3791/1869-v PMC3149993 20461051

[35] Roadmap Epigenomics C Kundaje A Meuleman W. Integrative analysis of 111 reference human epigenomes. Nature (2015) 518(7539):317–30. doi: 10.1038/nature14248 PMC4530010 25693563

[36] Wu MC Lee S Cai T Li Y Boehnke M Lin X. Rare-variant association testing for sequencing data with the sequence kernel association test. Am J Hum Genet (2011) 89(1):82–93. doi: 10.1016/j.ajhg.2011.05.029 21737059 PMC3135811

[37] Lee S Wu MC Lin X. Optimal tests for rare variant effects in sequencing association studies. Biostatistics (2012) 13(4):762–75. doi: 10.1093/biostatistics/kxs014 PMC3440237 22699862

[38] Ionita-Laza I Lee S Makarov V Buxbaum JD Lin X. Sequence kernel association tests for the combined effect of rare and common variants. Am J Hum Genet (2013) 92(6):841–53. doi: 10.1016/j.ajhg.2013.04.015 PMC3675243 23684009

[39] Zhu Y Tazearslan C Suh Y. Challenges and progress in interpretation of non-coding genetic variants associated with human disease. Exp Biol Med (Maywood). (2017) 242(13):1325–34. doi: 10.1177/1535370217713750 PMC5529005 28581336

[40] Shendre A Wiener HW Zhi D Vazquez AI Portman MA Shrestha S. High-density genotyping of immune loci in Kawasaki disease and IVIG treatment response in European-American case-parent trio study. Genes Immun (2014) 15(8):534–42. doi: 10.1038/gene.2014.47 PMC4257866 25101798

[41] Wang H Song W Hu T Zhang N Miao S Zong S. Fank1 interacts with Jab1 and regulates cell apoptosis *via* the AP-1 pathway. Cell Mol Life Sci (2011) 68(12):2129–39. doi: 10.1007/s00018-010-0559-4 PMC11114715 20978819

[42] Haase M Fitze G. HSP90AB1: Helping the good and the bad. Gene (2016) 575(2 Pt 1):171–86. doi: 10.1016/j.gene.2015.08.063 PMC5675009 26358502

[43] Graner MW. HSP90 and immune modulation in cancer. Adv Cancer Res (2016) 129:191–224. doi: 10.1016/bs.acr.2015.10.001 26916006

[44] Lin T Qiu Y Peng W Peng L. Heat shock protein 90 family isoforms as prognostic biomarkers and their correlations with immune infiltration in breast cancer. BioMed Res Int (2020) 2020:2148253. doi: 10.1155/2020/2148253 33145341 PMC7596464

[45] Dubaniewicz A. Microbial and human heat shock proteins as 'danger signals' in sarcoidosis. Hum Immunol (2013) 74(12):1550–8. doi: 10.1016/j.humimm.2013.08.275 23993988

[46] Zhang M Gu Y Huang S Lou Q Xie Q Xu Z. Copy number variations and polymorphisms in HSP90AB1 and risk of systemic lupus erythematosus and efficacy of glucocorticoids. J Cell Mol Med (2019) 23(8):5340–8. doi: 10.1111/jcmm.14410 PMC6653051 31124601

[47] Binstadt BA Geha RS Bonilla FA. IgG Fc receptor polymorphisms in human disease: implications for intravenous immunoglobulin therapy. J Allergy Clin Immunol (2003) 111(4):697–703. doi: 10.1067/mai.2003.1380 12704346

[48] Tsujimoto H Takeshita S Nakatani K Kawamura Y Tokutomi T Sekine I. Intravenous immunoglobulin therapy induces neutrophil apoptosis in Kawasaki disease. Clin Immunol (2002) 103(2):161–8. doi: 10.1006/clim.2002.5209 12027421

[49] Su K Li X Edberg JC Wu J Ferguson P Kimberly RP. A promoter haplotype of the immunoreceptor tyrosine-based inhibitory motif-bearing FcgammaRIIb alters receptor expression and associates with autoimmunity. II. Differential binding of GATA4 and Yin-Yang1 transcription factors and correlated receptor expression and function. J Immunol (2004) 172(11):7192–9. doi: 10.4049/jimmunol.172.11.7192 15153544

[50] Su K Wu J Edberg JC Li X Ferguson P Cooper GS. A promoter haplotype of the immunoreceptor tyrosine-based inhibitory motif-bearing FcgammaRIIb alters receptor expression and associates with autoimmunity. I. Regulatory FCGR2B polymorphisms and their association with systemic lupus erythematosus. J Immunol (2004) 172(11):7186–91. doi: 10.4049/jimmunol.172.11.7186 15153543

[51] Li X Wu J Carter RH Edberg JC Su K Cooper GS. A novel polymorphism in the Fcgamma receptor IIB (CD32B) transmembrane region alters receptor signaling. Arthritis Rheumatol (2003) 48(11):3242–52. doi: 10.1002/art.11313 14613290

[52] Kimberly RP Wu J Gibson AW Su K Qin H Li X. Diversity and duplicity: human FCgamma receptors in host defense and autoimmunity. Immunol Res (2002) 26(1-3):177–89. doi: 10.1385/IR:26:1-3:177 12403356

[53] Herscovics A. Structure and function of Class I alpha 1,2-mannosidases involved in glycoprotein synthesis and endoplasmic reticulum quality control. Biochimie (2001) 83(8):757–62. doi: 10.1016/S0300-9084(01)01319-0 11530208

[54] Esmail S Manolson MF. Advances in understanding N-glycosylation structure, function, and regulation in health and disease. Eur J Cell Biol (2021) 100(7-8):151186. doi: 10.1016/j.ejcb.2021.151186 34839178

[55] Marth JD Grewal PK. Mammalian glycosylation in immunity. Nat Rev Immunol (2008) 8(11):874–87. doi: 10.1038/nri2417 PMC2768770 18846099

[56] Ercan A Cui J Chatterton DE Deane KD Hazen MM Brintnell W. Aberrant IgG galactosylation precedes disease onset, correlates with disease activity, and is prevalent in autoantibodies in rheumatoid arthritis. Arthritis Rheumatol (2010) 62(8):2239–48. doi: 10.1002/art.27533 PMC4118465 20506563

[57] Su Z Xie Q Wang Y Li Y. Abberant Immunoglobulin G Glycosylation in Rheumatoid Arthritis by LTQ-ESI-MS. Int J Mol Sci. (2020) 21(6):2045. doi: 10.3390/ijms21062045 32192063 PMC7139372

[58] Portman MA Wiener HW Silva M Shendre A Shrestha S. DC-SIGN gene promoter variants and IVIG treatment response in Kawasaki disease. Pediatr Rheumatol Online J (2013) 11(1):32. doi: 10.1186/1546-0096-11-32 24006904 PMC3847673

[59] Ojha H Ghosh P Singh Panwar H Shende R Gondane A Mande SC. Spatially conserved motifs in complement control protein domains determine functionality in regulators of complement activation-family proteins. Commun Biol (2019) 2:290. doi: 10.1038/s42003-019-0529-9 31396570 PMC6683126

[60] Lau WL Scholnick SB. Identification of two new members of the CSMD gene family. Genomics (2003) 82(3):412–5. doi: 10.1016/S0888-7543(03)00149-6 12906867

[61] Gutierrez MA Dwyer BE Franco SJ. Csmd2 is a synaptic transmembrane protein that interacts with PSD-95 and is required for neuronal maturation. eNeuro (2019) 6(2). doi: 10.1523/ENEURO.0434-18.2019 PMC6506821 31068362

[62] Zhang R Song C. Loss of CSMD1 or 2 may contribute to the poor prognosis of colorectal cancer patients. Tumour Biol (2014) 35(5):4419–23. doi: 10.1007/s13277-013-1581-6 24408017

[63] Zhang H Huang T Ren X Fang X Chen X Wei H. Integrated pan-cancer analysis of CSMD2 as a potential prognostic, diagnostic, and immune biomarker. Front Genet (2022) 13:918486. doi: 10.3389/fgene.2022.918486 36061177 PMC9428318

[64] Freeman BD MaChado FS Tanowitz HB Desruisseaux MS. Endothelin-1 and its role in the pathogenesis of infectious diseases. Life Sci (2014) 118(2):110–9. doi: 10.1016/j.lfs.2014.04.021 PMC4538933 24780317

[65] Shinagawa S Okazaki T Ikeda M Yudoh K Kisanuki YY Yanagisawa M. T cells upon activation promote endothelin 1 production in monocytes *via* IFN-gamma and TNF-alpha. Sci Rep (2017) 7(1):14500. doi: 10.1038/s41598-017-14202-5 29101349 PMC5670167

[66] Xu M Qi Q Men L Wang S Li M Xiao M. Berberine protects Kawasaki disease-induced human coronary artery endothelial cells dysfunction by inhibiting of oxidative and endoplasmic reticulum stress. Vascul Pharmacol (2020) 127:106660. doi: 10.1016/j.vph.2020.106660 32070767

[67] Ogawa S Zhang J Yuge K Watanabe M Fukazawa R Kamisago M. Increased plasma endothelin-1 concentration in Kawasaki disease. J Cardiovasc Pharmacol (1993) 22 Suppl 8:S364–6. doi: 10.1097/00005344-199322008-00095 7509988

[68] Gupta RM Hadaya J Trehan A Zekavat SM Roselli C Klarin D. A genetic variant associated with five vascular diseases is a distal regulator of endothelin-1 gene expression. Cell (2017) 170(3):522–33 e15. doi: 10.1016/j.cell.2017.06.049 28753427 PMC5785707

[69] Saw J Yang ML Trinder M Tcheandjieu C Xu C Starovoytov A. Chromosome 1q21.2 and additional loci influence risk of spontaneous coronary artery dissection and myocardial infarction. Nat Commun (2020) 11(1):4432. doi: 10.1038/s41467-020-17558-x 32887874 PMC7474092

