# Supplementary material for: Pharmacogenomics of intravenous immunoglobulin response in Kawasaki disease
Source: Front Immunol. 2024 Jan 8;14:1287094. doi: 10.3389/fimmu.2023.1287094 (PMC10800400; doi:10.3389/fimmu.2023.1287094)
Supplement: Supplementary file 1 [file DataSheet_1.pdf]

Supplemental Methods

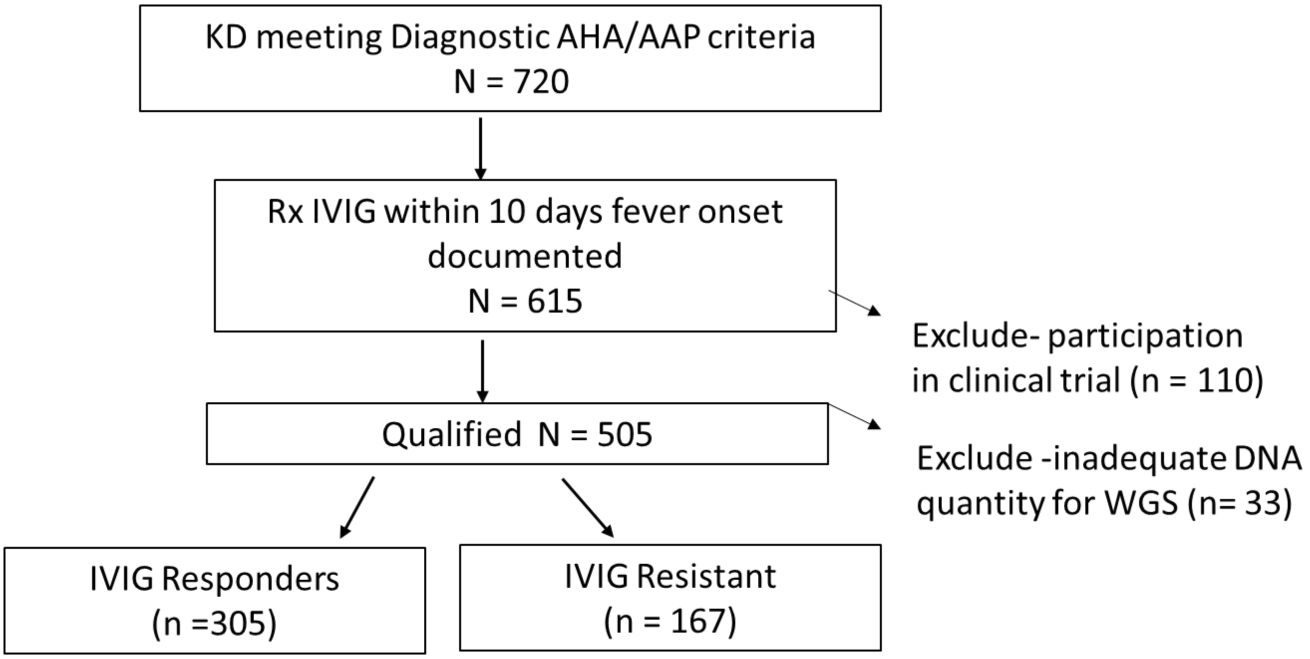

Flow diagram describes selection of patients for inclusion in whole genome sequencing study. North American patients were enrolled in data and DNA biorepository at Seattle Children's Research Institute. Patients were included if they met KD diagnostic criteria published in a scientific statement by the American Heart Association,[1] and then were treated with intravenous gamma globulin (2 gm/kg) within 10 days of fever onset. Patients were excluded for participation in a clinical trial which might impact outcome or if quantity of DNA was insufficient for whole genome sequencing.

AHA diagnostic criteria for "Complete Kawasaki Diagnosis" extracted from the AHA Statement (2017). "The KD complete diagnosis requires presence of fever for at least 5 days together with at least 4 of the 5 following principal features: 1. Erythema and cracking of lips, strawberry tongue, and/or erythema of oral and pharyngeal mucosa; 2. Bilateral bulbar conjunctival injection without exudate; 3. Rash: maculopapular, diffuse erythroderma, or erythema multiform-like; 4. Erythema and edema of the hands and feet in acute phase and/or periungual desquamation in subacute phase; 5. Cervical lymphadenopathy (≥1.5 cm diameter), usually unilateral.). If coronary artery abnormalities are detected, the diagnosis of KD is considered confirmed in most cases. The diagnosis of "Incomplete KD" can be made with fever presence, CRP > 3 gm/dl or ESR > 40mm/hr and 2 to 3 criteria in addition to at least 3 supplemental laboratory values including 1)anemia for age; 2) platelet count ≥450,000 after the seventh day of fever; 3) serum albumin ≤3.0 gm/dl; 4) elevated ALT; 5) WBC count of ≥ 15,000/mm^3; 6) urine ≥10 WBC/hp; Or positive echocardiogram. IVIG resistance was defined as persistent or recurrent fever greater than 36 hours after completing IVIG infusion.

1. McCrindle BW, Rowley AH, Newburger JW, Burns JC, Bolger AF, Gewitz M, Baker AL, Jackson MA, Takahashi M, Shah PB, et al. Diagnosis, Treatment, and Long-Term Management of Kawasaki Disease: A Scientific Statement for Health Professionals From the American Heart Association. *Circulation*. 2017;135:e927-e999. doi: 10.1161/CIR.0000000000000484
