# Supplementary material for: Pharmacogenomics of intravenous immunoglobulin response in Kawasaki disease
Source: Front Immunol. 2024 Jan 8;14:1287094. doi: 10.3389/fimmu.2023.1287094 (PMC10800400; doi:10.3389/fimmu.2023.1287094)

Supplemental Figure 1

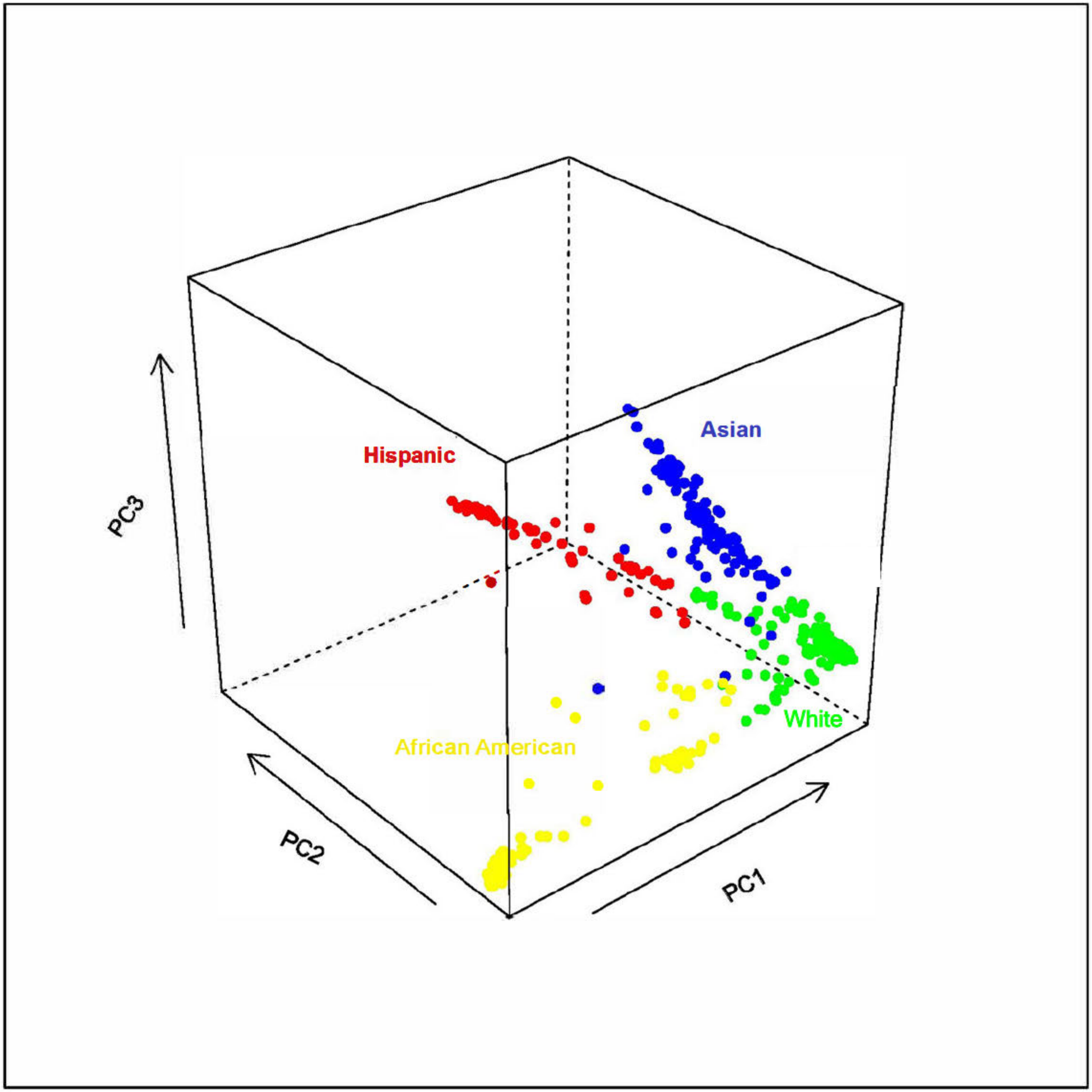

Supplemental Figure 2a

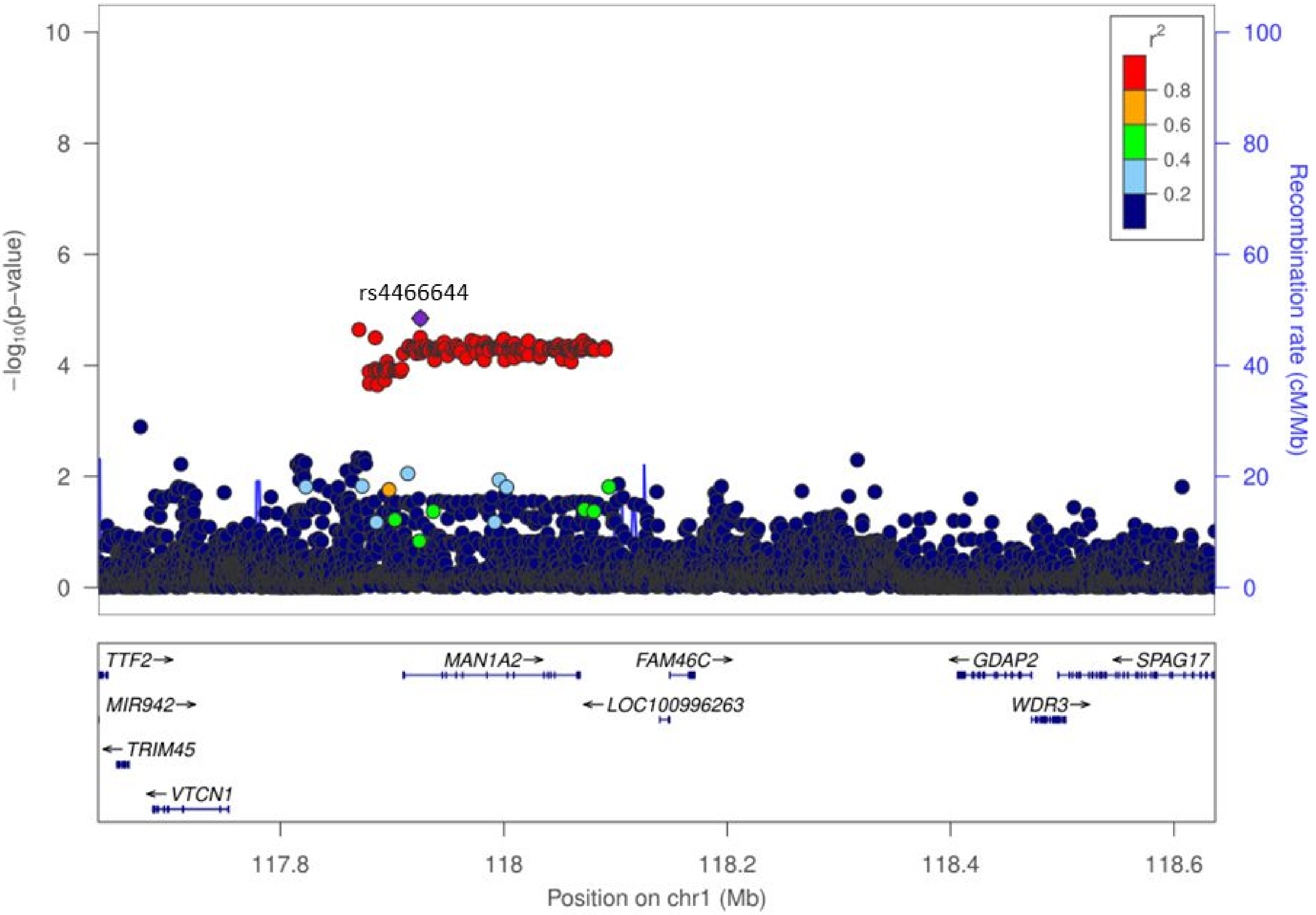

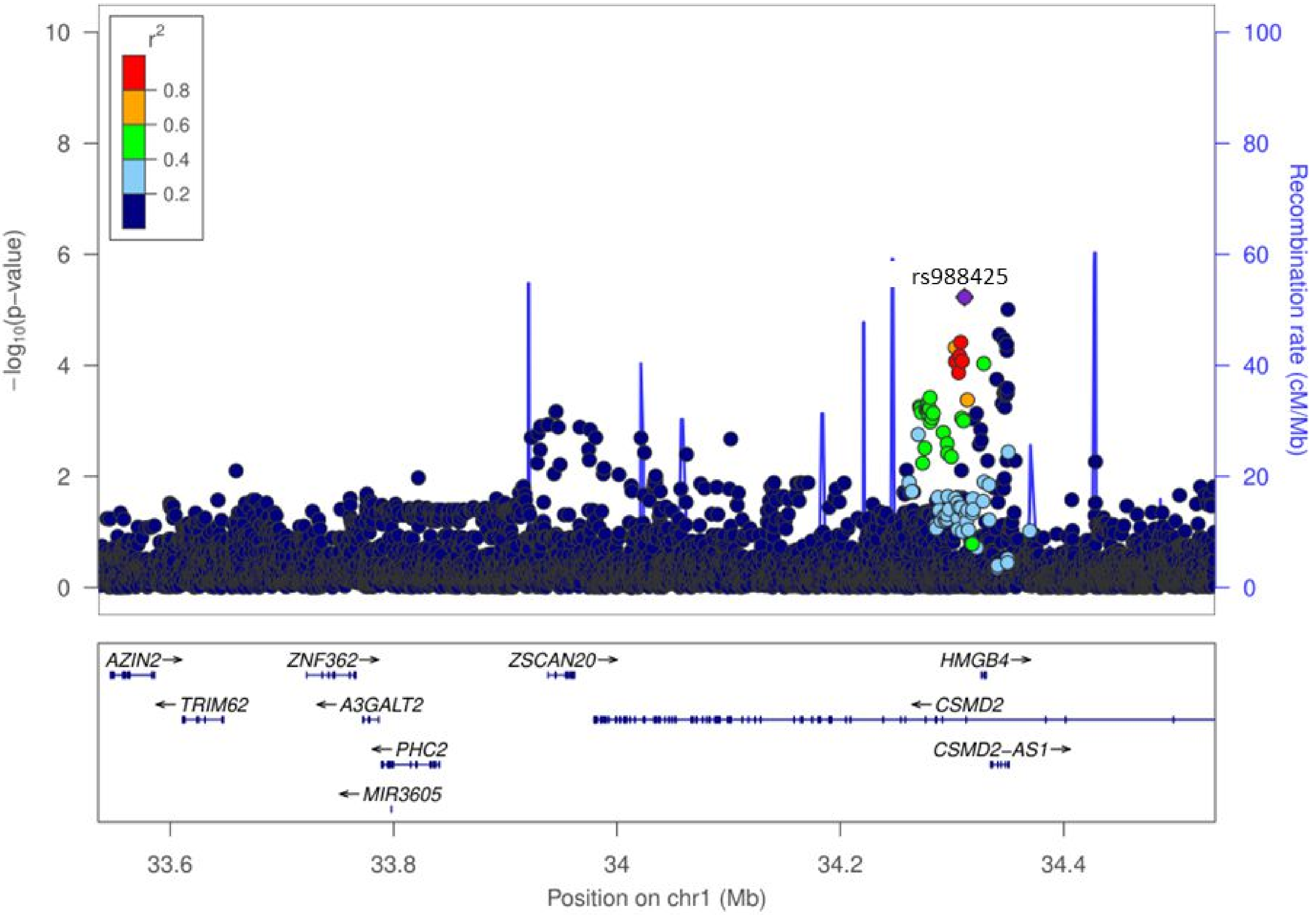
r2
0.8
0.6
0.4
0.2
10
8
6
4
2
0
−log10(p−value)
100
80
60
40
20
0
Recombination rate (cM/Mb)
rs988425
AZIN2→
←TRIM62
ZNF362→
←A3GALT2
←PHC2
←MIR3605
ZSCAN20→
HMGB4→
←CSMD2
CSMD2−AS1→
33.6
33.8
34
34.2
34.4
Position on chr1 (Mb)

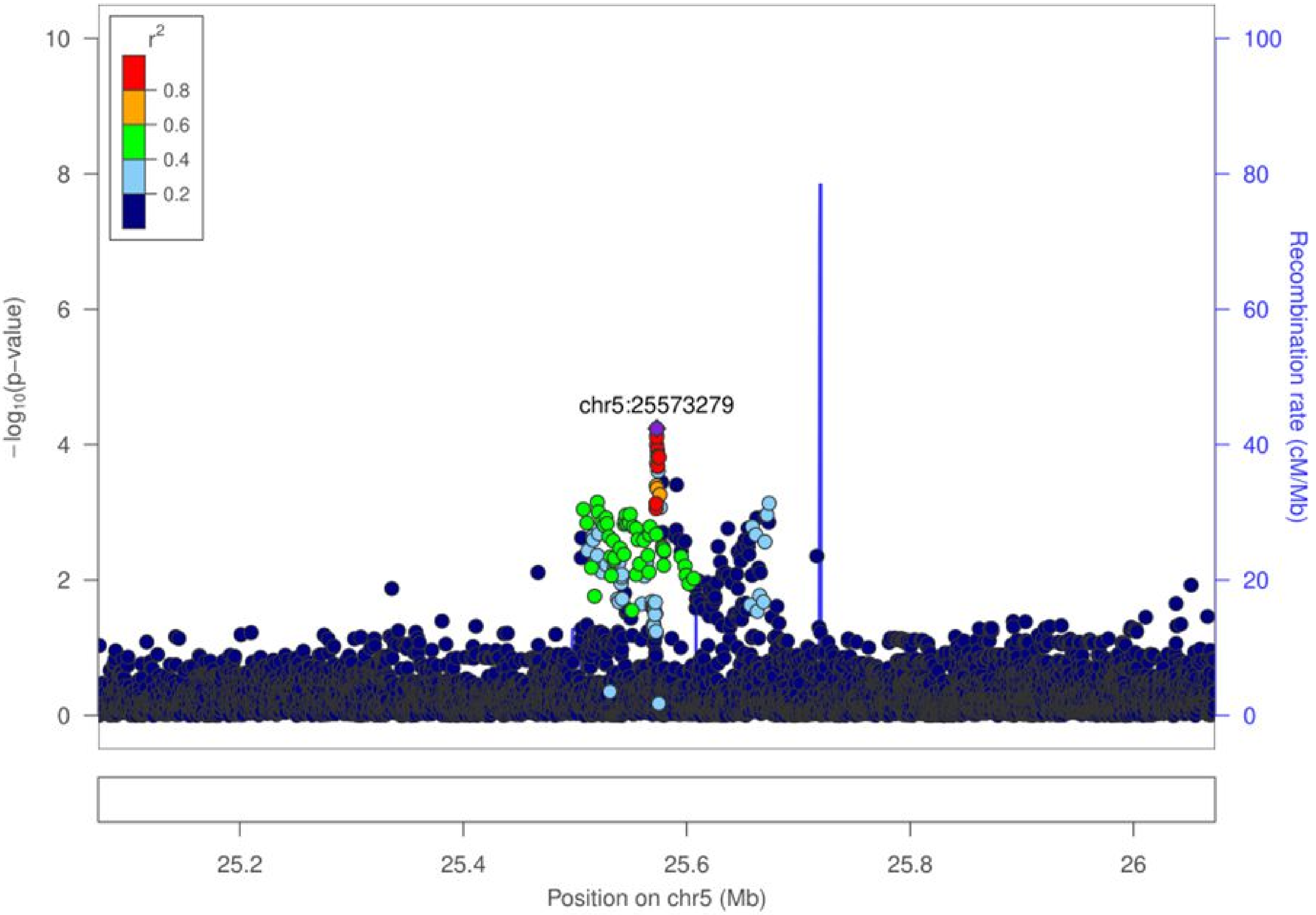

r2
0.8
0.6
0.4
0.2
chr5:25573279
−log10(p−value)
Recombination rate (cM/Mb)
Position on chr5 (Mb)
25.2
25.4
25.6
25.8
26
0
2
4
6
8
10
20
40
60
80
100

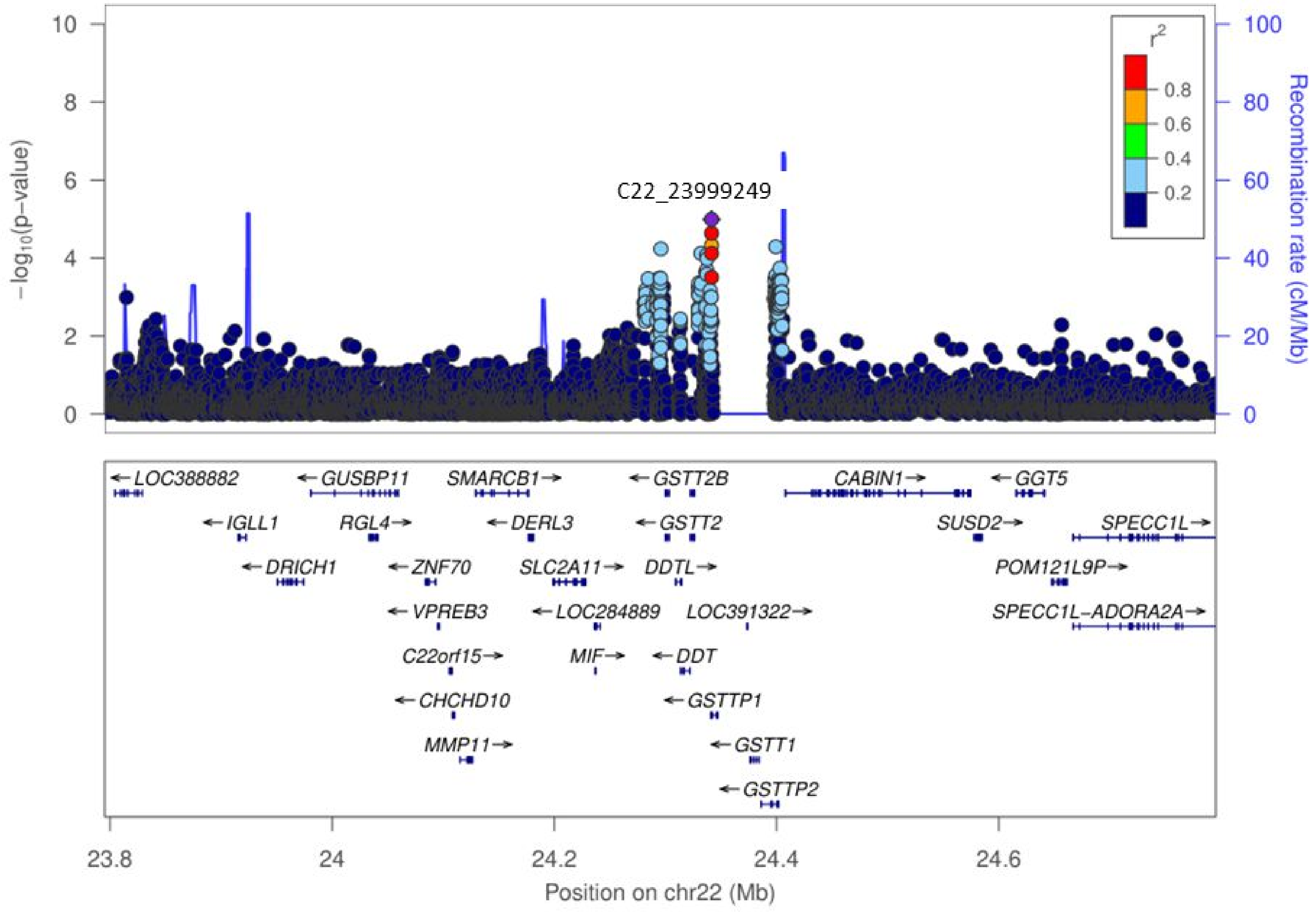

C22_23999249
-log10(p-value)
Recombination rate (cM/Mb)
r2
0.8
0.6
0.4
0.2
LOC388882
GUSBP11
SMARCB1
GSTT2B
CABIN1
GGT5
IGLL1
RGL4
DERL3
GSTT2
SUSD2
SPECC1L
DRICH1
ZNF70
SLC2A11
DDTL
POM121L9P
VPREB3
LOC284889
LOC391322
SPECC1L-ADORA2A
C22orf15
MIF
DDT
CHCHD10
GSTTP1
MMP11
GSTT1
GSTTP2
23.8
24
24.2
24.4
24.6
Position on chr22 (Mb)

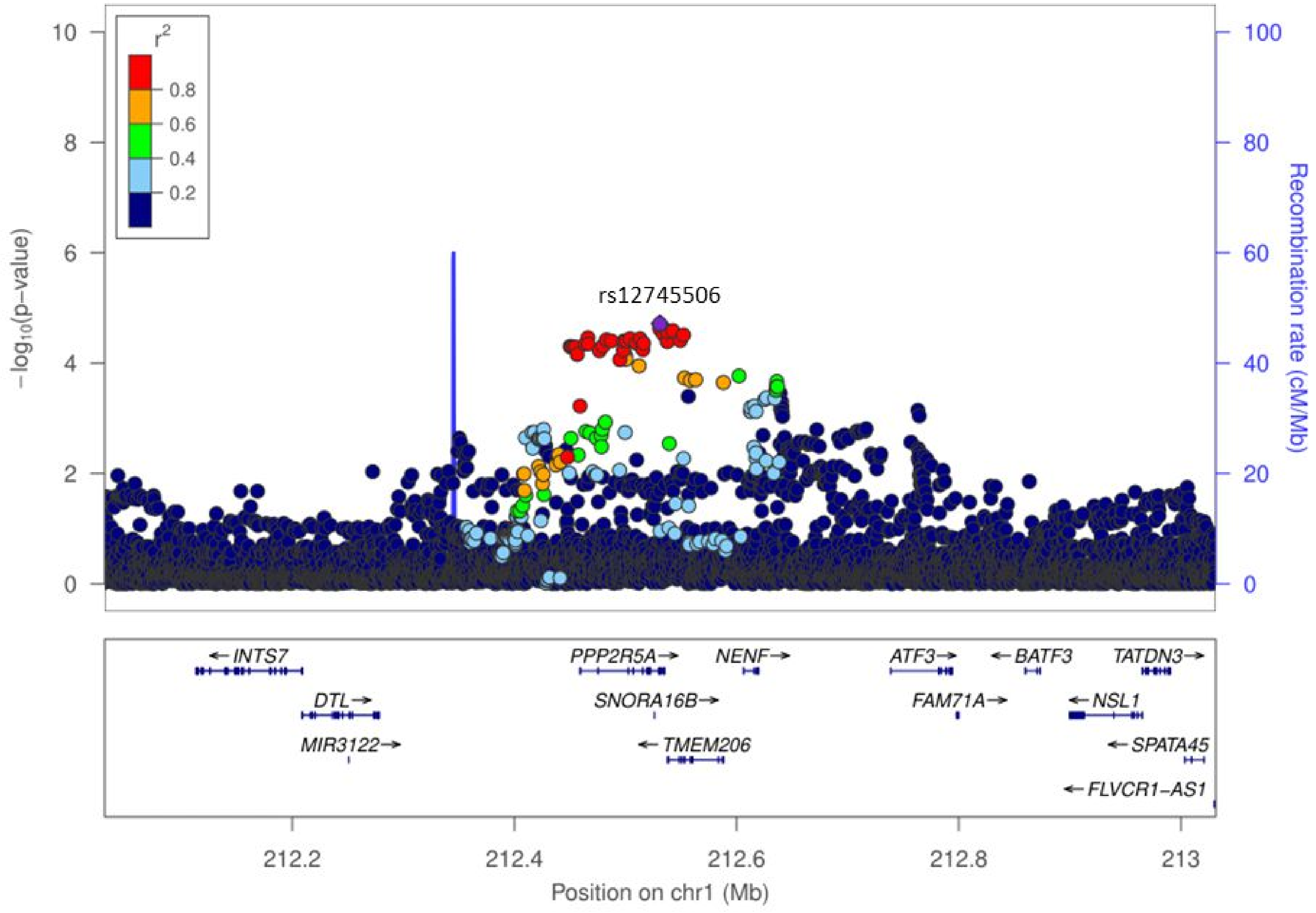

rs12745506
r2
0.8
0.6
0.4
0.2
-log10(p-value)
Recombination rate (cM/Mb)
INTS7
DTL
MIR3122
PPP2R5A
SNORA16B
TMEM206
NENF
ATF3
FAM71A
BATF3
NSL1
TATDN3
SPATA45
FLVCR1-AS1
212.2
212.4
212.6
212.8
213
Position on chr1 (Mb)

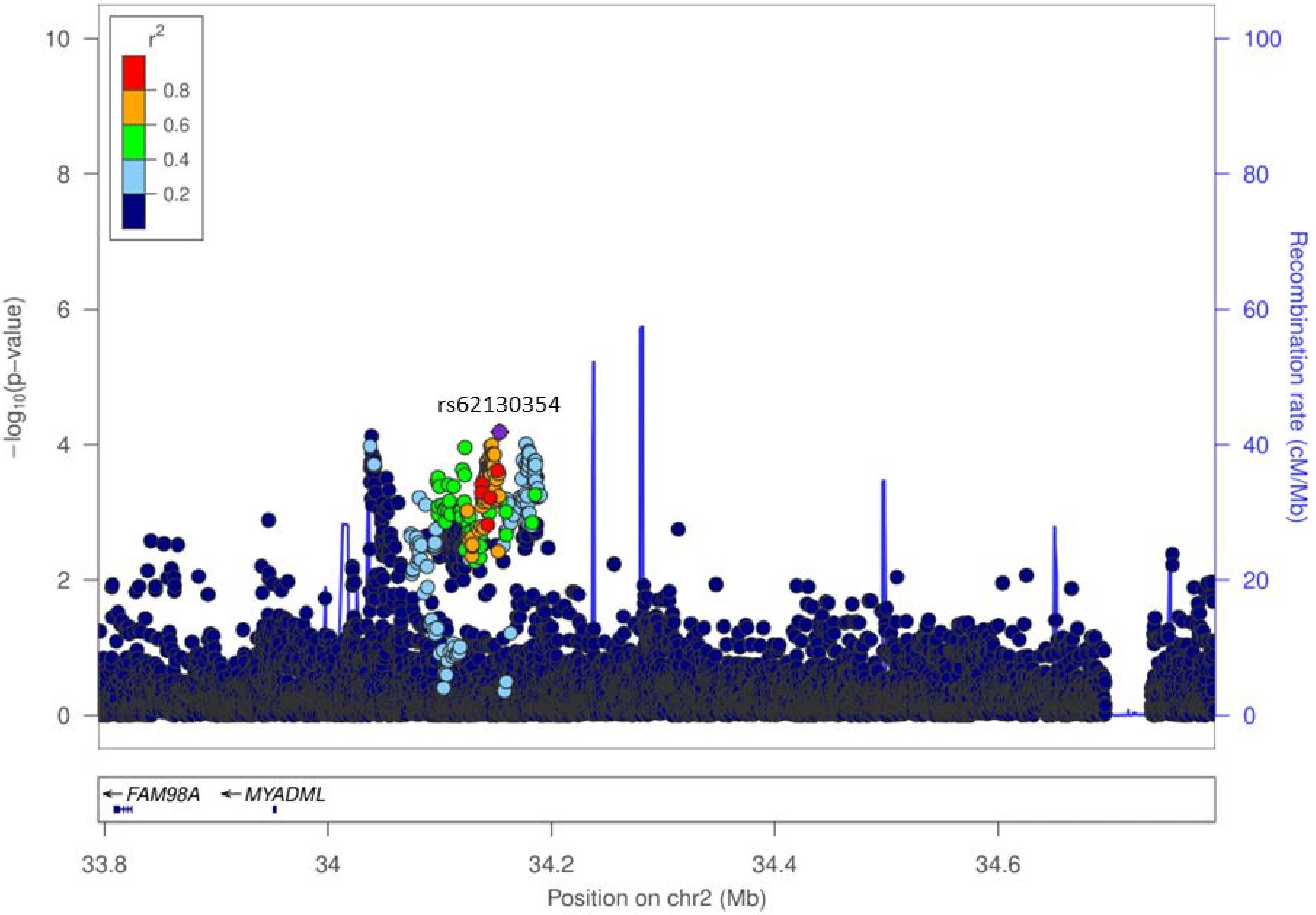

r2
0.8
0.6
0.4
0.2
rs62130354
-log10(p-value)
Recombination rate (cM/Mb)
0
2
4
6
8
10
0
20
40
60
80
100
FAM98A
MYADML
33.8
34
34.2
34.4
34.6
Position on chr2 (Mb)

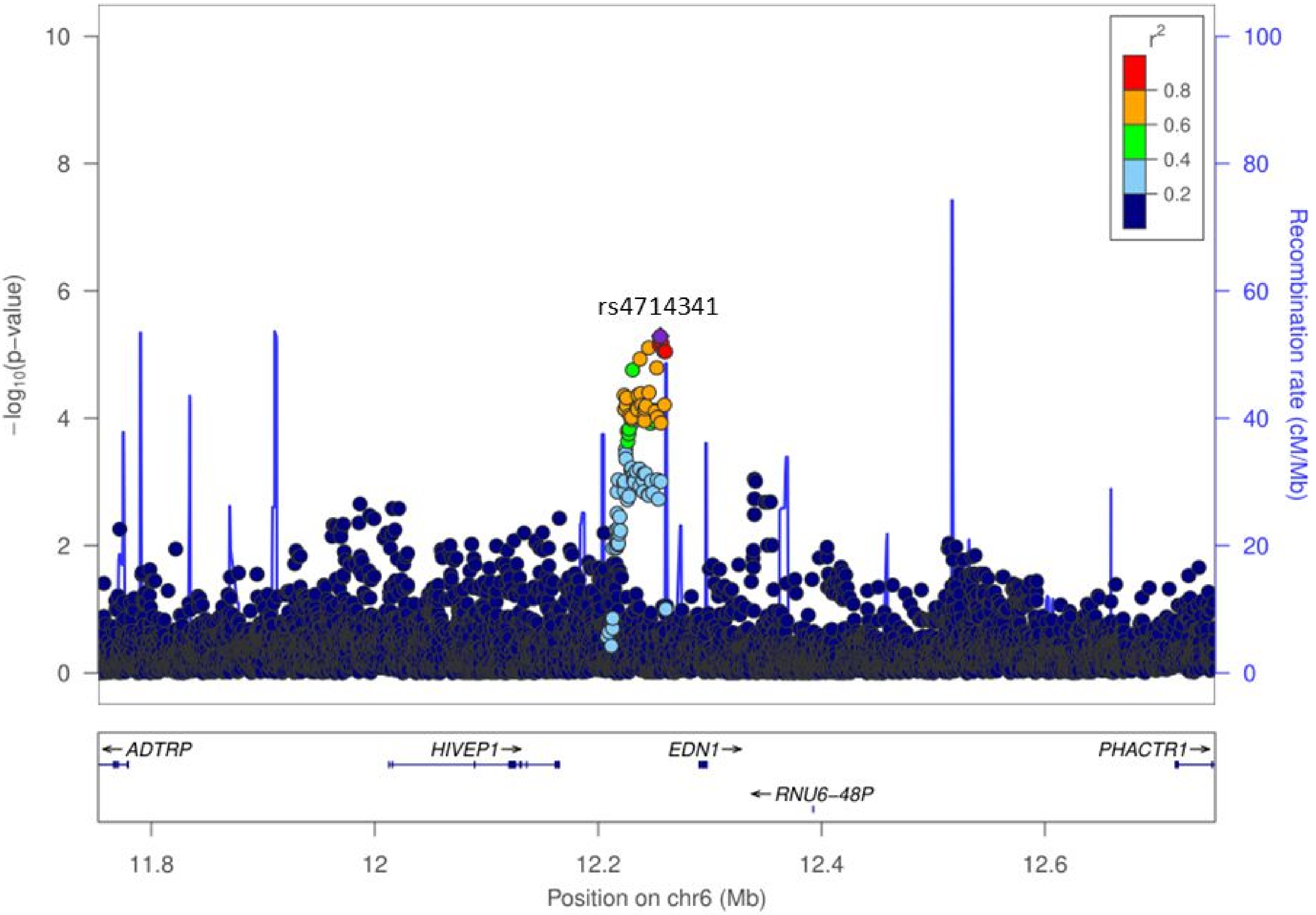

rs4714341
r2
0.8
0.6
0.4
0.2
-log10(p-value)
Recombination rate (cM/Mb)
ADTRP
HIVEP1
EDN1
PHACTR1
RNU6-48P
11.8
12
12.2
12.4
12.6
Position on chr6 (Mb)

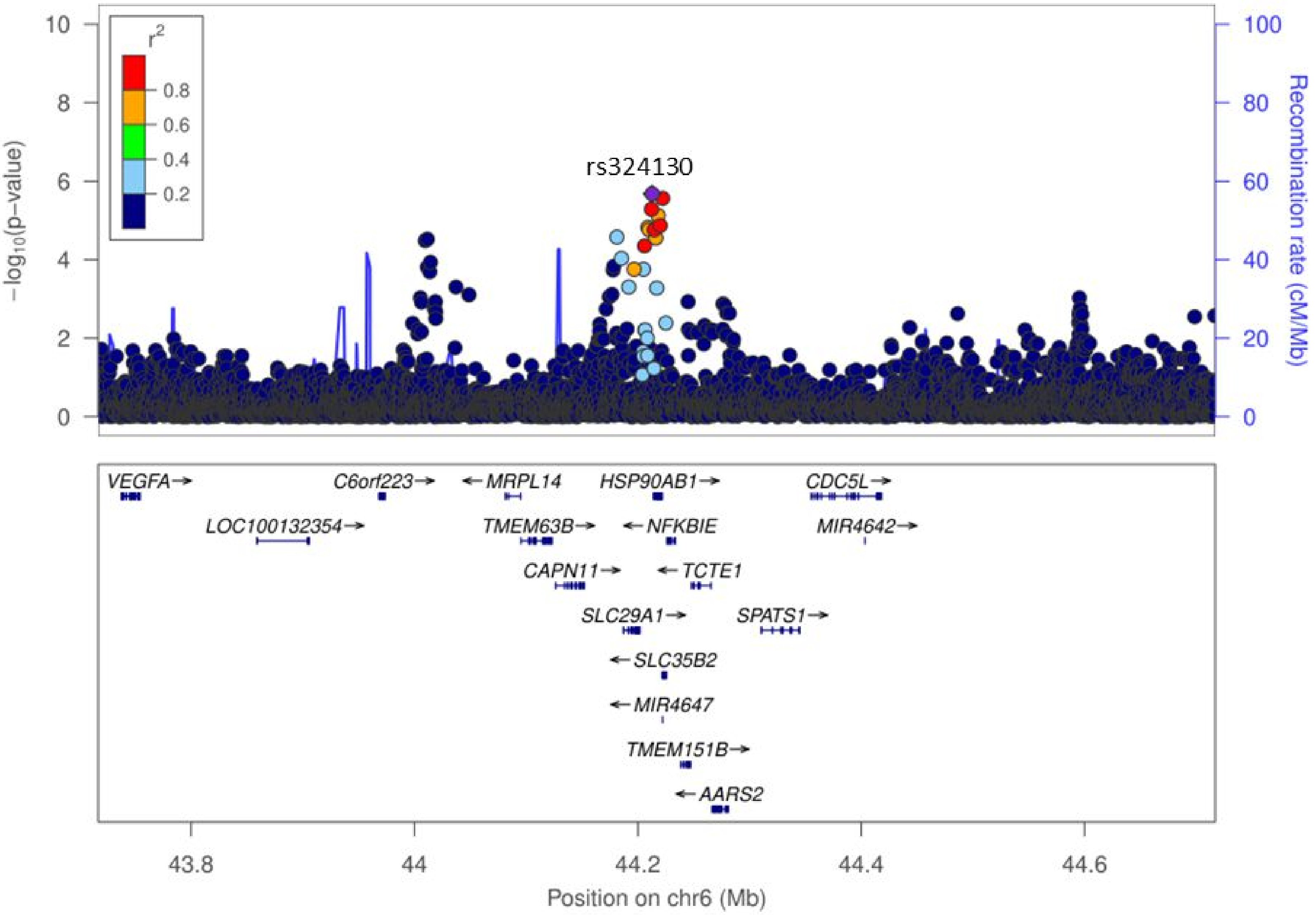
rs324130
r2
0.8
0.6
0.4
0.2
-log10(p-value)
Recombination rate (cM/Mb)
10
8
6
4
2
0
100
80
60
40
20
0
VEGFA→
C6orf223→
←MRPL14
HSP90AB1→
CDC5L→
LOC100132354→
TMEM63B→
←NFKBIE
MIR4642→
CAPN11→
←TCTE1
SLC29A1→
SPATS1→
←SLC35B2
←MIR4647
TMEM151B→
←AARS2
43.8
44
44.2
44.4
44.6
Position on chr6 (Mb)

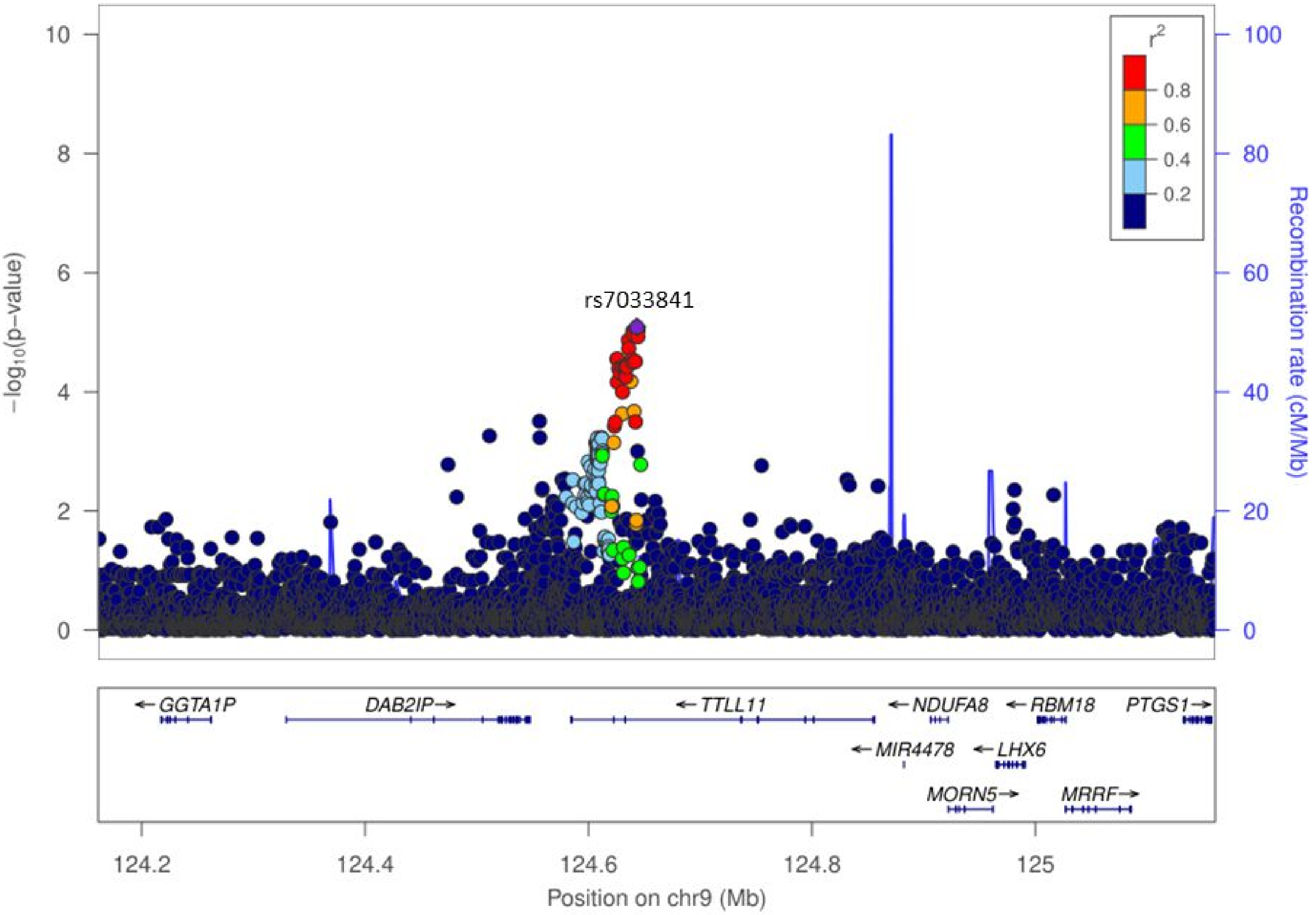
r2
0.8
0.6
0.4
0.2
rs7033841
−log10(p−value)
Recombination rate (cM/Mb)
0
2
4
6
8
10
0
20
40
60
80
100
←GGTA1P
DAB2IP→
←TTLL11
←NDUFA8
←RBM18
PTGS1→
←MIR4478
←LHX6
MORN5→
MRRF→
124.2
124.4
124.6
124.8
125
Position on chr9 (Mb)

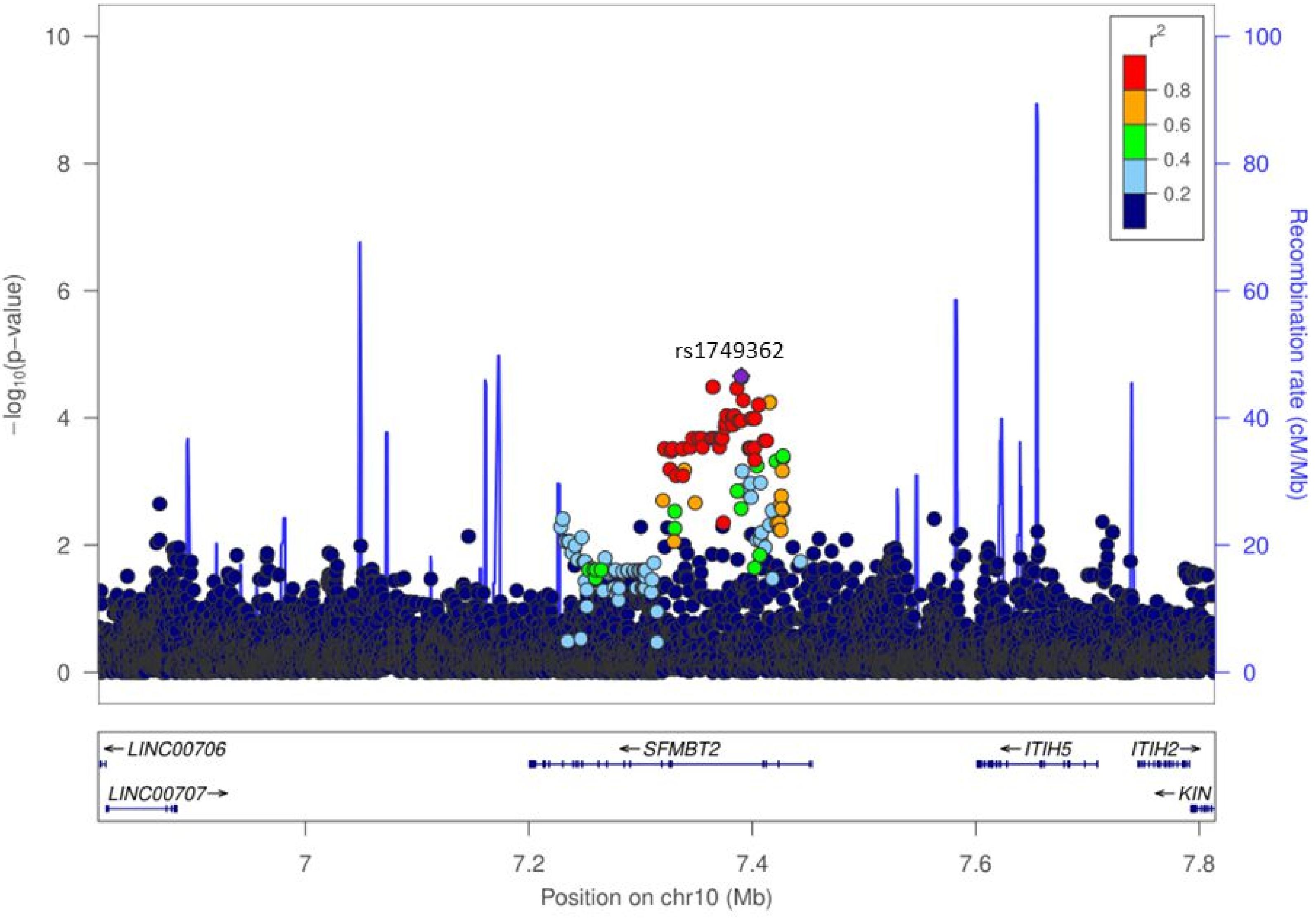

rs1749362
r2
0.8
0.6
0.4
0.2
−log10(p−value)
Recombination rate (cM/Mb)
0
2
4
6
8
10
0
20
40
60
80
100
LINC00706
LINC00707
SFMBT2
ITIH5
ITIH2
KIN
7
7.2
7.4
7.6
7.8
Position on chr10 (Mb)

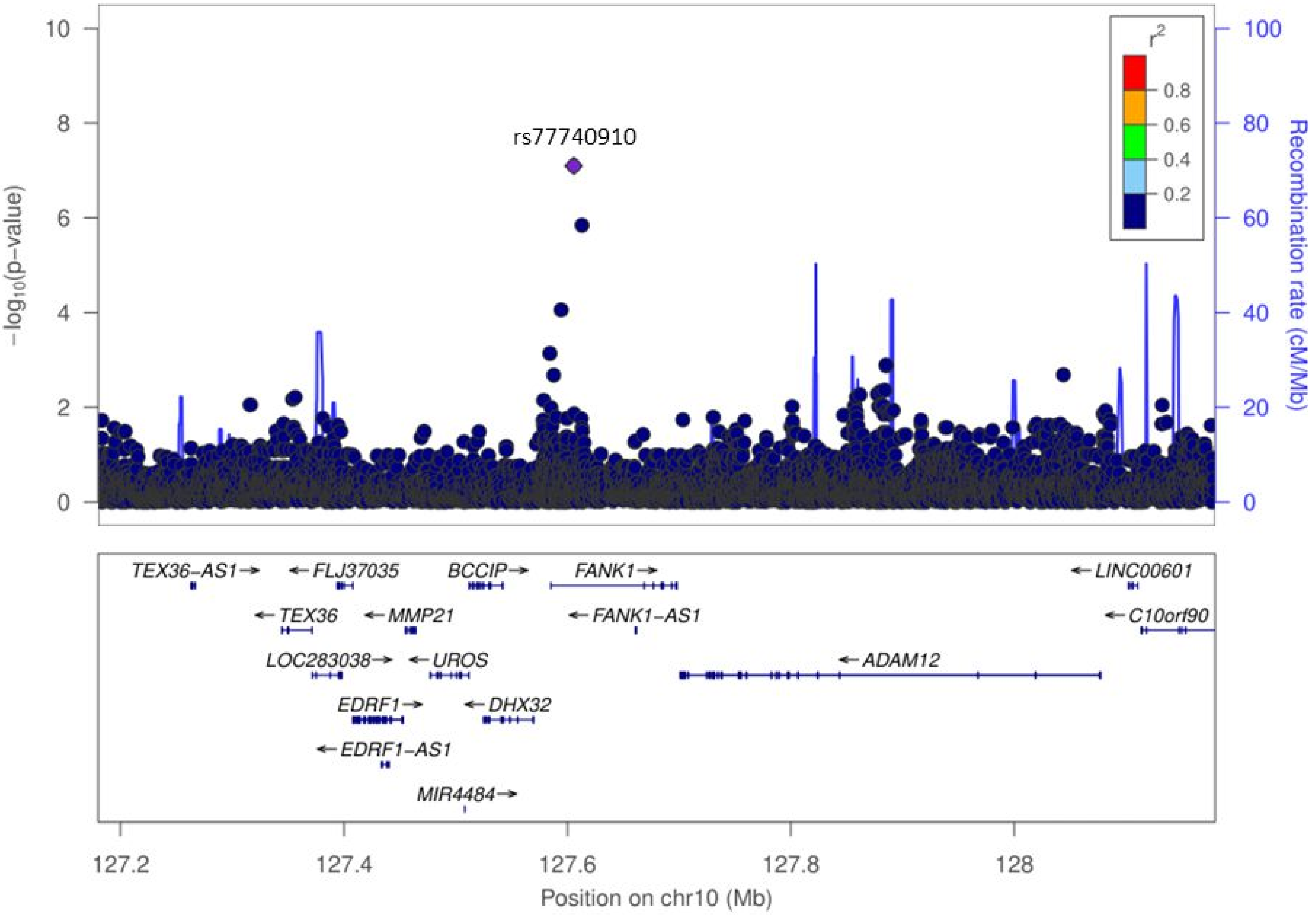

rs77740910
−log10(p−value)
Recombination rate (cM/Mb)
r2
0.8
0.6
0.4
0.2
TEX36−AS1→
←FLJ37035
BCCIP→
FANK1→
←LINC00601
←TEX36
←MMP21
←FANK1−AS1
←C10orf90
LOC283038→
←UROS
←ADAM12
EDRF1→
←DHX32
←EDRF1−AS1
MIR4484→
127.2
127.4
127.6
127.8
128
Position on chr10 (Mb)

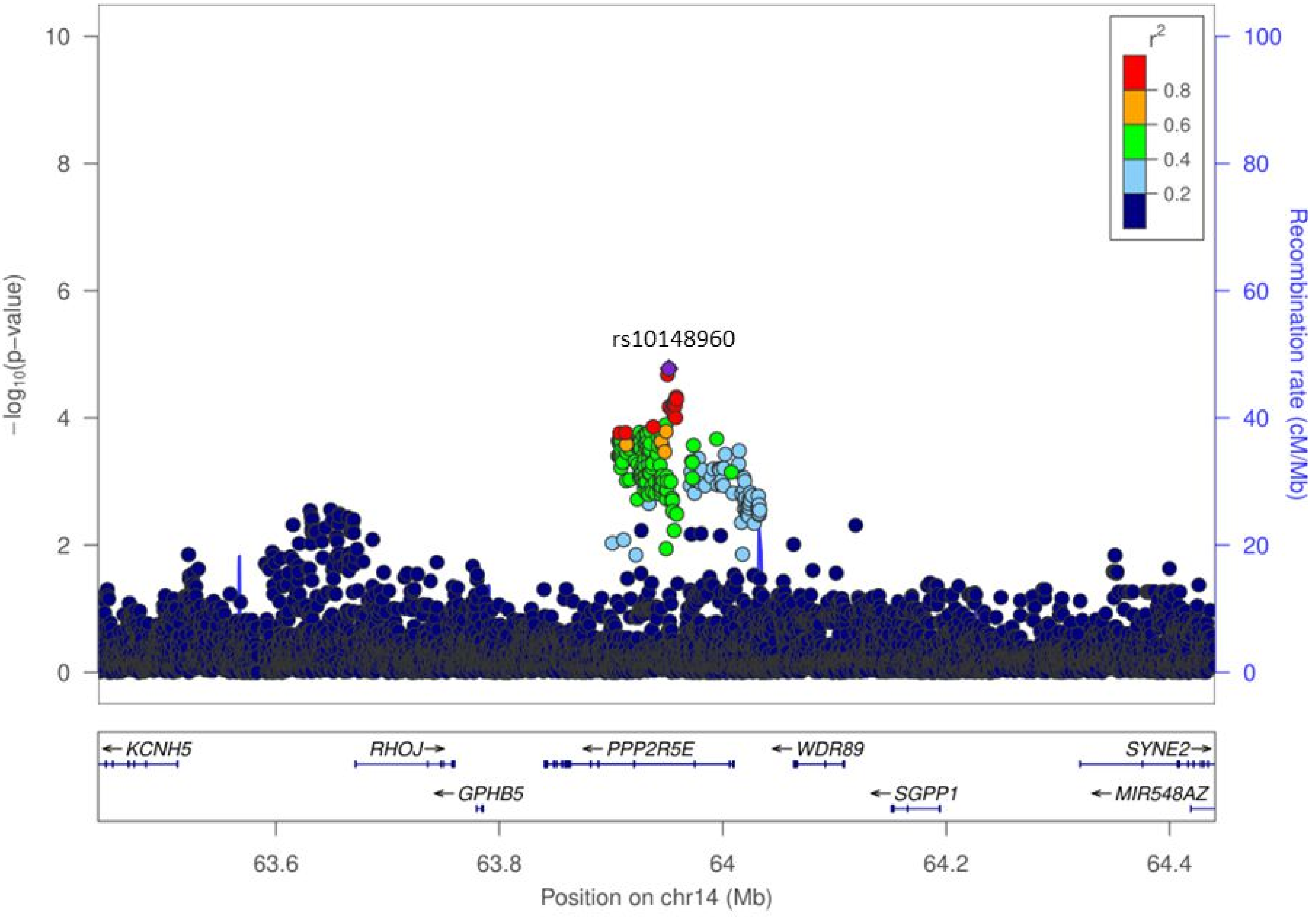
rs10148960
−log10(p−value)
Recombination rate (cM/Mb)
r2
0.8
0.6
0.4
0.2
0
2
4
6
8
10
20
40
60
80
100
KCNH5
RHOJ
PPP2R5E
WDR89
SYNE2
GPHB5
SGPP1
MIR548AZ
63.6
63.8
64
64.2
64.4
Position on chr14 (Mb)

Supplemental Figure 2b

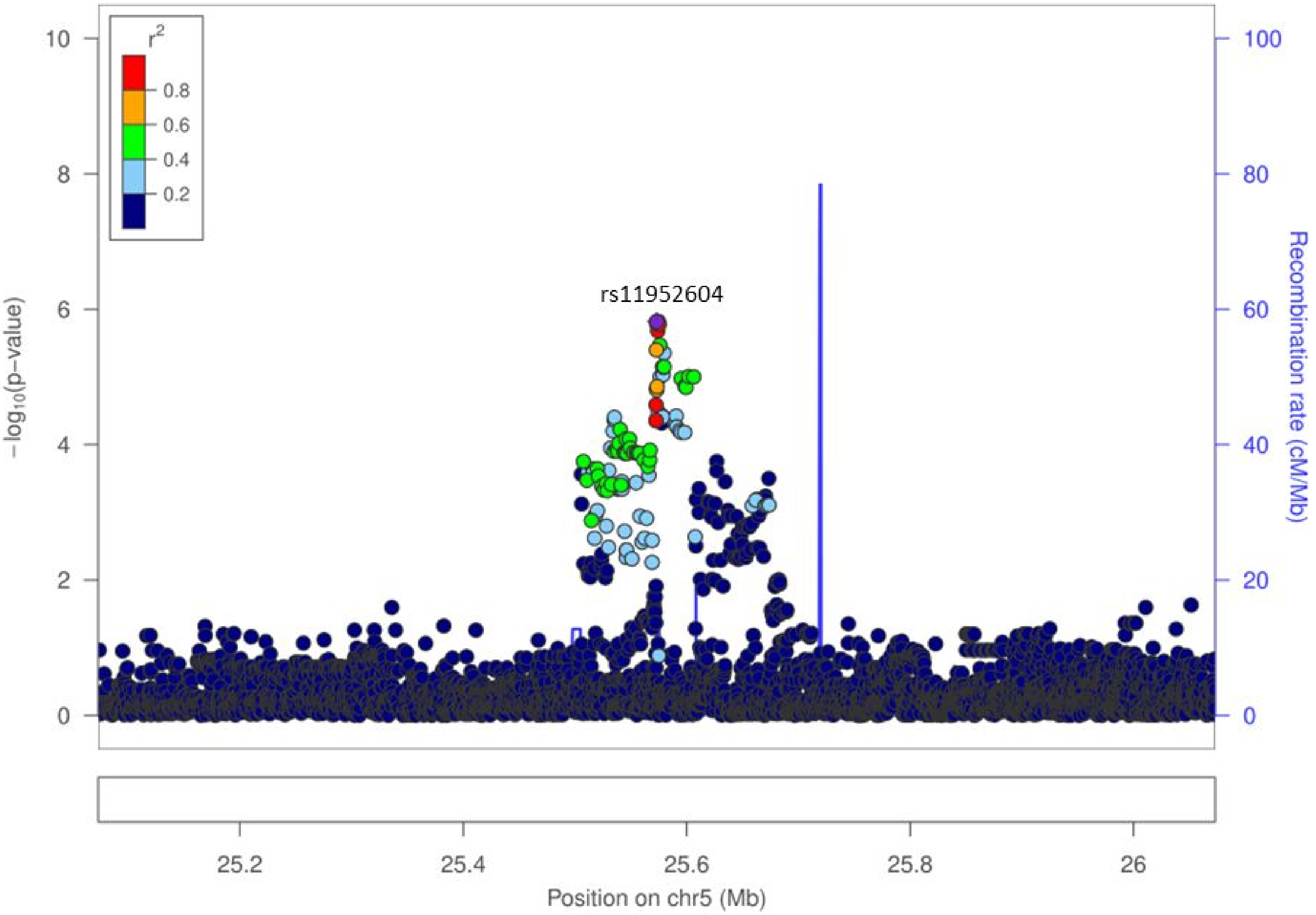

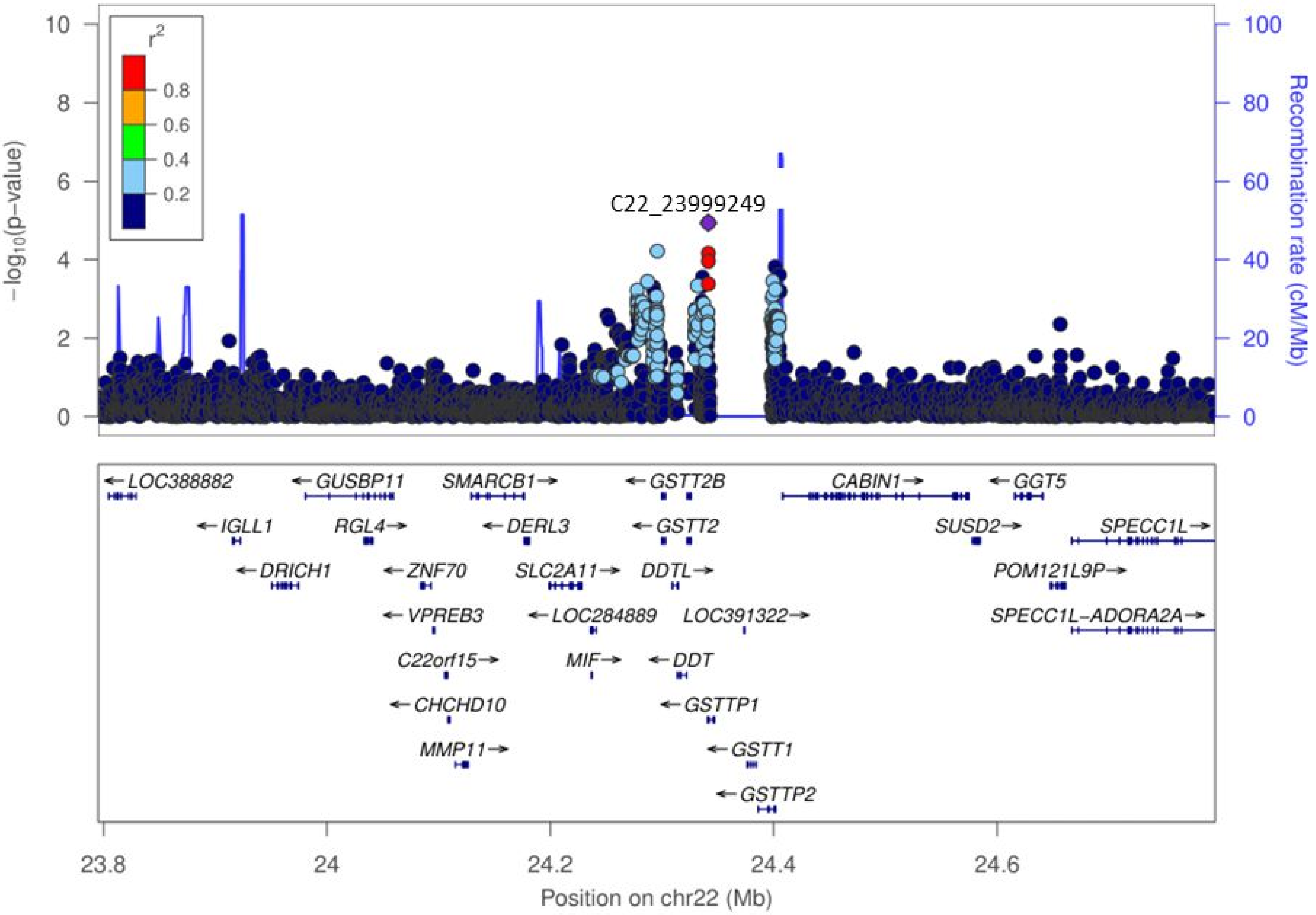

r²
0.8
0.6
0.4
0.2
C22_23999249
−log10(p−value)
Recombination rate (cM/Mb)
0
2
4
6
8
10
0
20
40
60
80
100
← LOC388882
← GUSBP11
SMARCB1 →
← GSTT2B
CABIN1 →
← GGT5
← IGLL1
RGL4 →
← DERL3
← GSTT2
SUSD2 →
SPECC1L →
← DRICH1
← ZNF70
SLC2A11 →
DDTL →
POM121L9P →
← VPREB3
← LOC284889
LOC391322 →
SPECC1L−ADORA2A →
C22orf15 →
MIF →
← DDT
← CHCHD10
← GSTTP1
MMP11 →
← GSTT1
← GSTTP2
23.8
24
24.2
24.4
24.6
Position on chr22 (Mb)

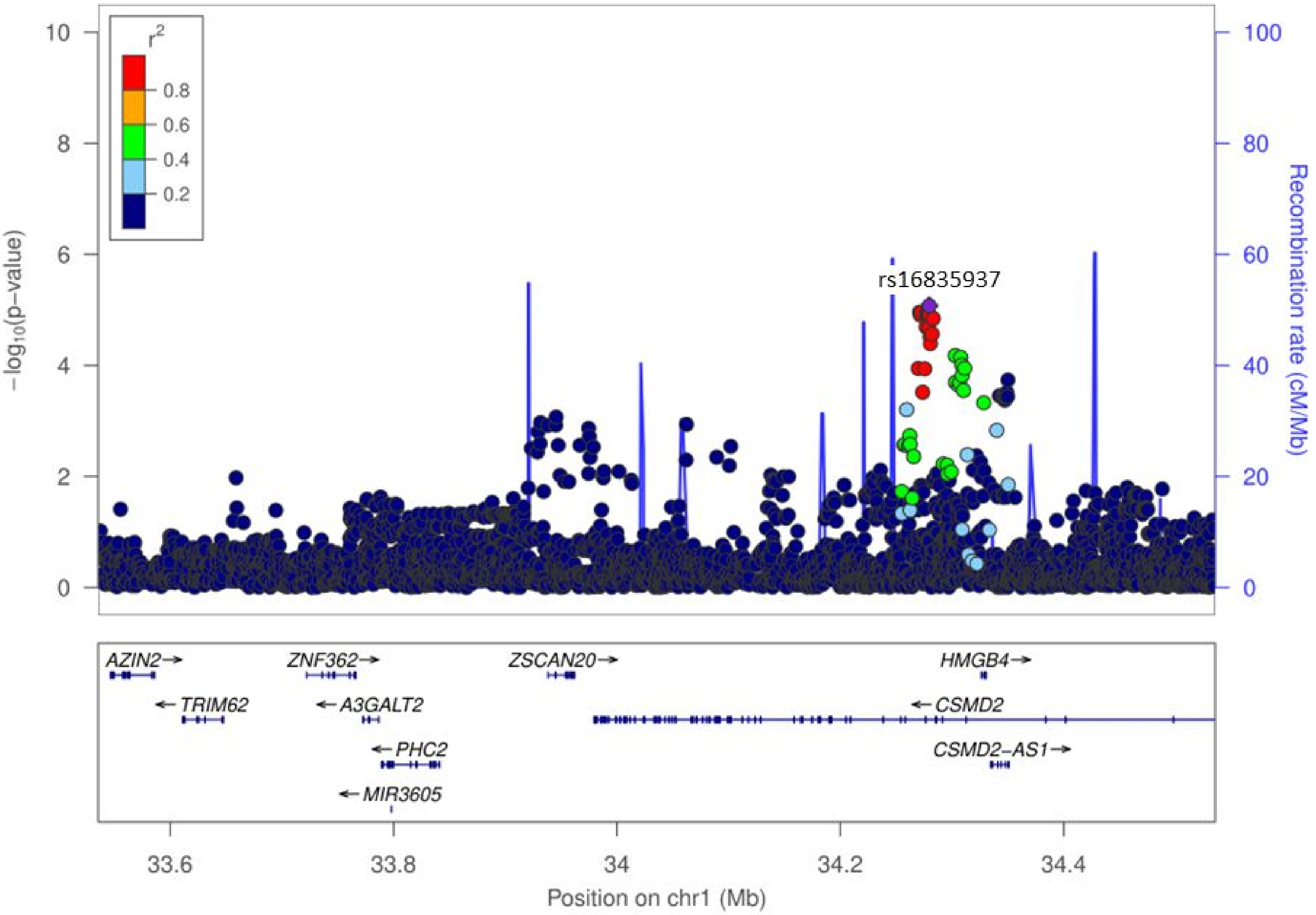

r2
0.8
0.6
0.4
0.2
−log10(p−value)
Recombination rate (cM/Mb)
rs16835937
AZIN2→
TRIM62
ZNF362→
A3GALT2
PHC2
MIR3605
ZSCAN20→
HMGB4→
CSMD2
CSMD2−AS1→
33.6
33.8
34
34.2
34.4
Position on chr1 (Mb)

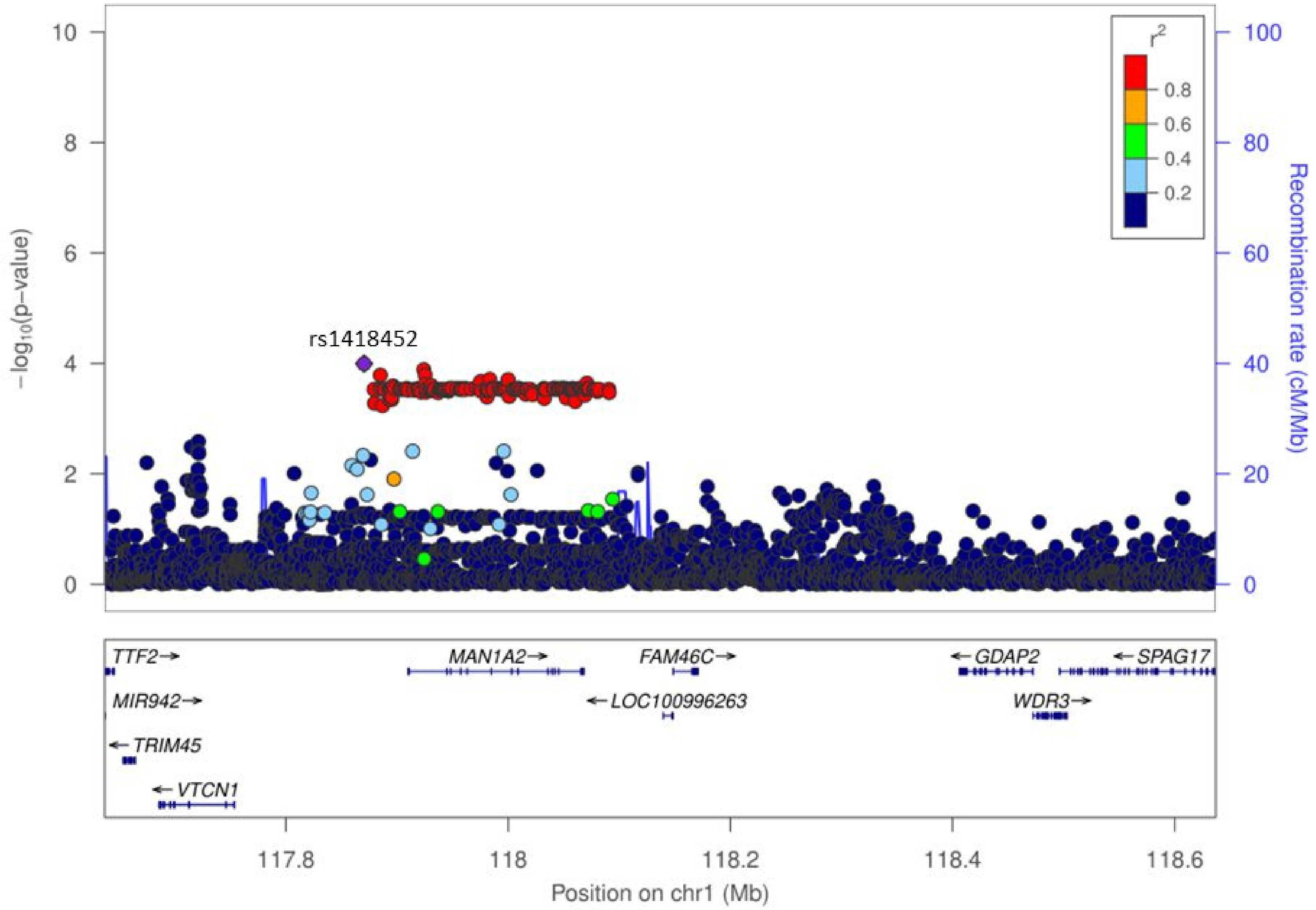

rs1418452
-log10(p-value)
Recombination rate (cM/Mb)
r2
0.8
0.6
0.4
0.2
TTF2→
MIR942→
←TRIM45
←VTCN1
MAN1A2→
FAM46C→
←LOC100996263
←GDAP2
←SPAG17
WDR3→
Position on chr1 (Mb)

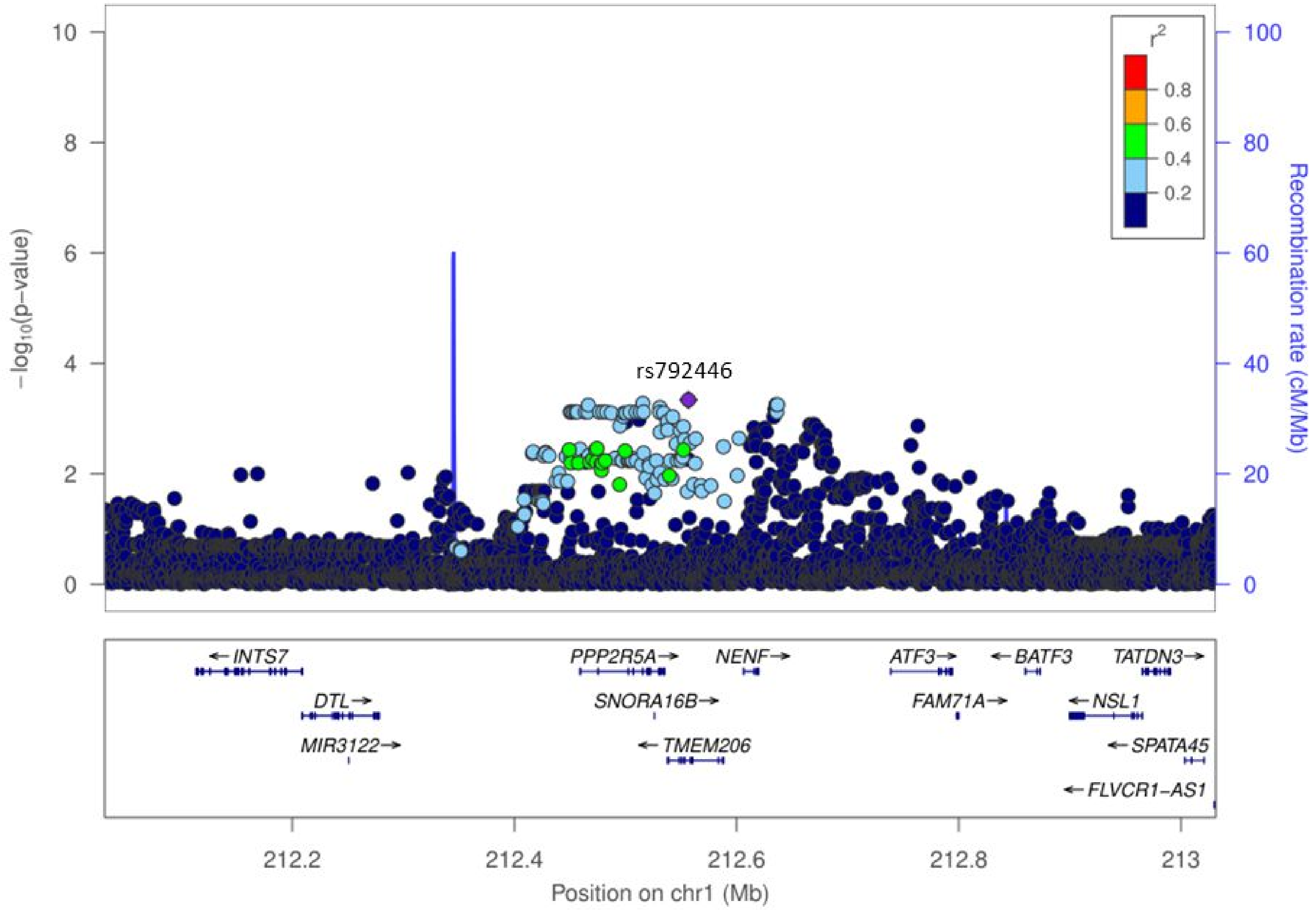
rs792446
−log10(p−value)
Recombination rate (cM/Mb)
r2
0.8
0.6
0.4
0.2
0
2
4
6
8
10
0
20
40
60
80
100
←INTS7
DTL→
MIR3122→
PPP2R5A→
SNORA16B→
←TMEM206
NENF→
ATF3→
FAM71A→
←BATF3
←NSL1
TATDN3→
←SPATA45
←FLVCR1-AS1
212.2
212.4
212.6
212.8
213
Position on chr1 (Mb)

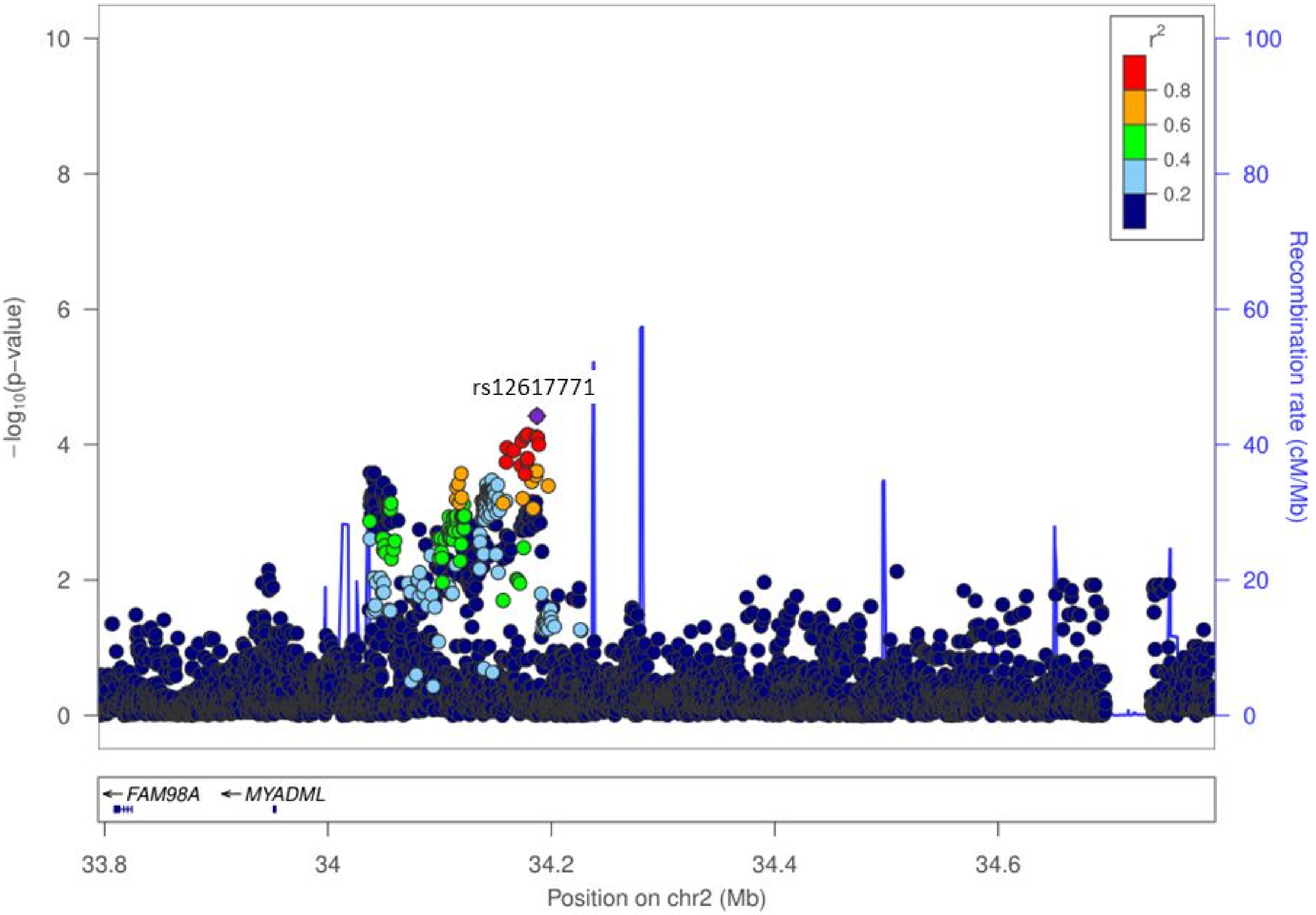
r2
0.8
0.6
0.4
0.2
rs12617771
−log10(p−value)
Recombination rate (cM/Mb)
FAM98A
MYADML
Position on chr2 (Mb)

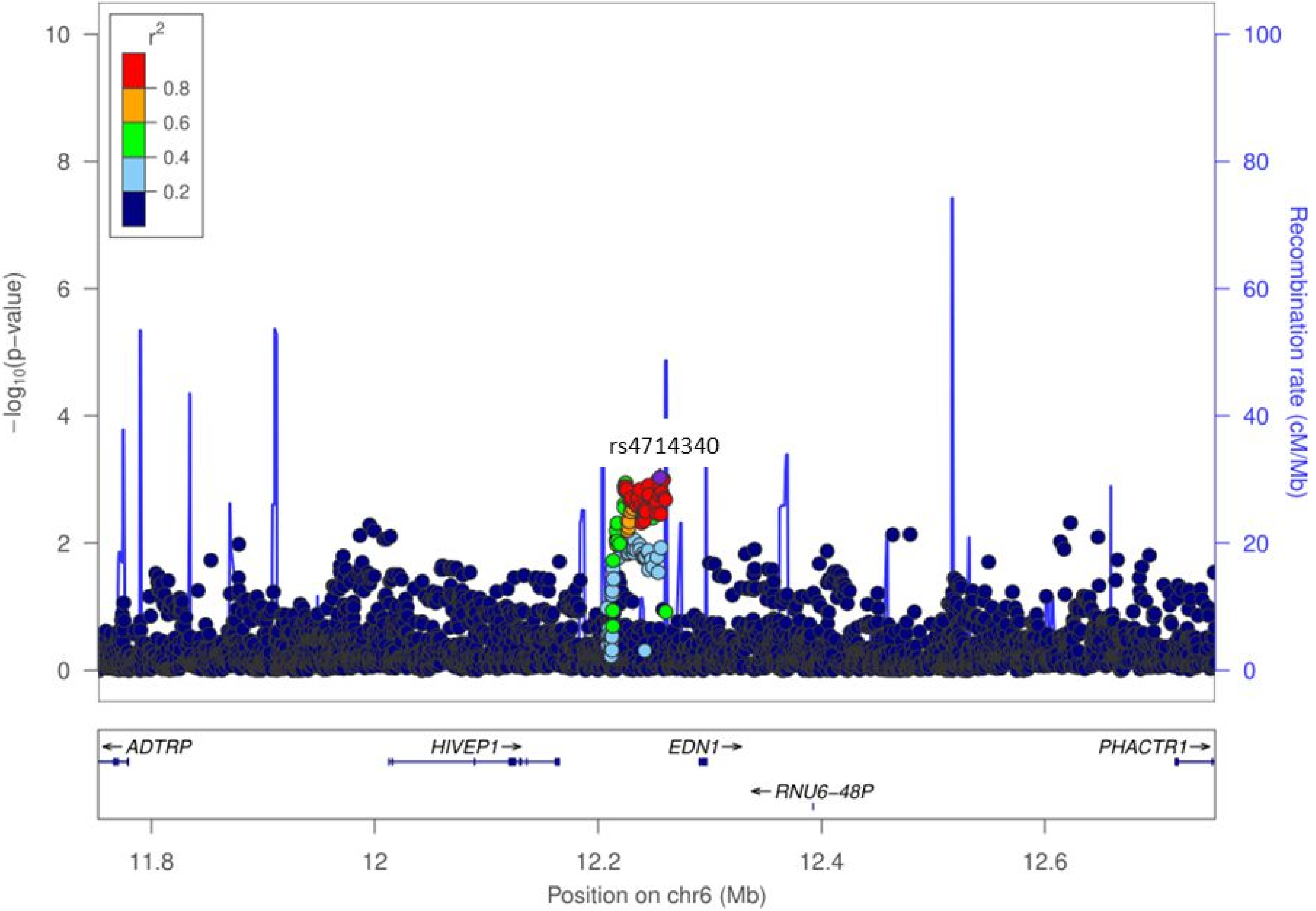
r²
0.8
0.6
0.4
0.2
10
8
6
4
2
0
−log10(p−value)
100
80
60
40
20
0
Recombination rate (cM/Mb)
rs4714340
ADTRP
HIVEP1
EDN1
PHACTR1
RNU6−48P
11.8
12
12.2
12.4
12.6
Position on chr6 (Mb)

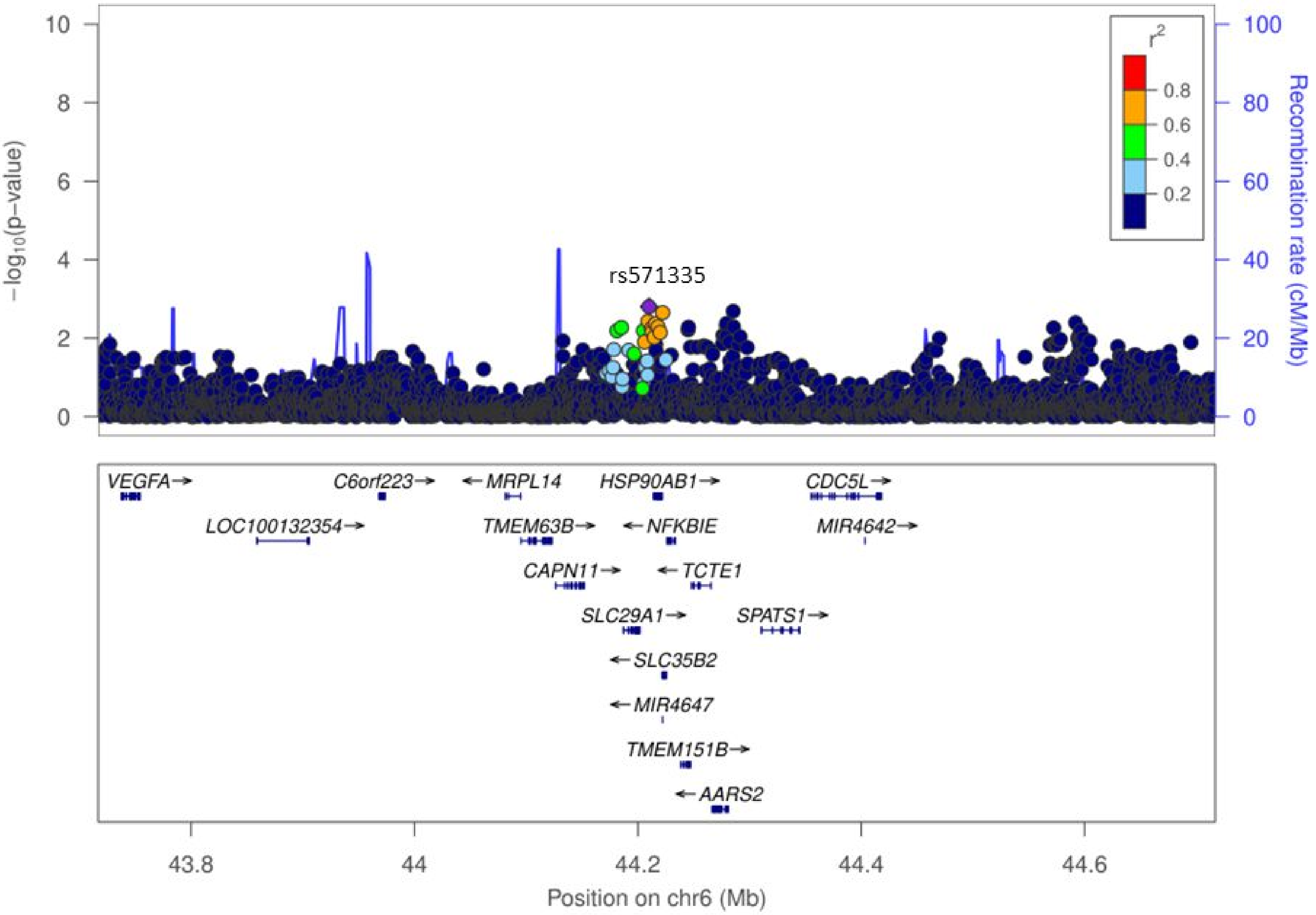

rs571335
r2
0.8
0.6
0.4
0.2
-log10(p-value)
Recombination rate (cM/Mb)
VEGFA
LOC100132354
C6orf223
MRPL14
TMEM63B
CAPN11
HSP90AB1
NFKBIE
TCTE1
SLC29A1
SLC35B2
MIR4647
TMEM151B
AARS2
SPATS1
CDC5L
MIR4642
Position on chr6 (Mb)

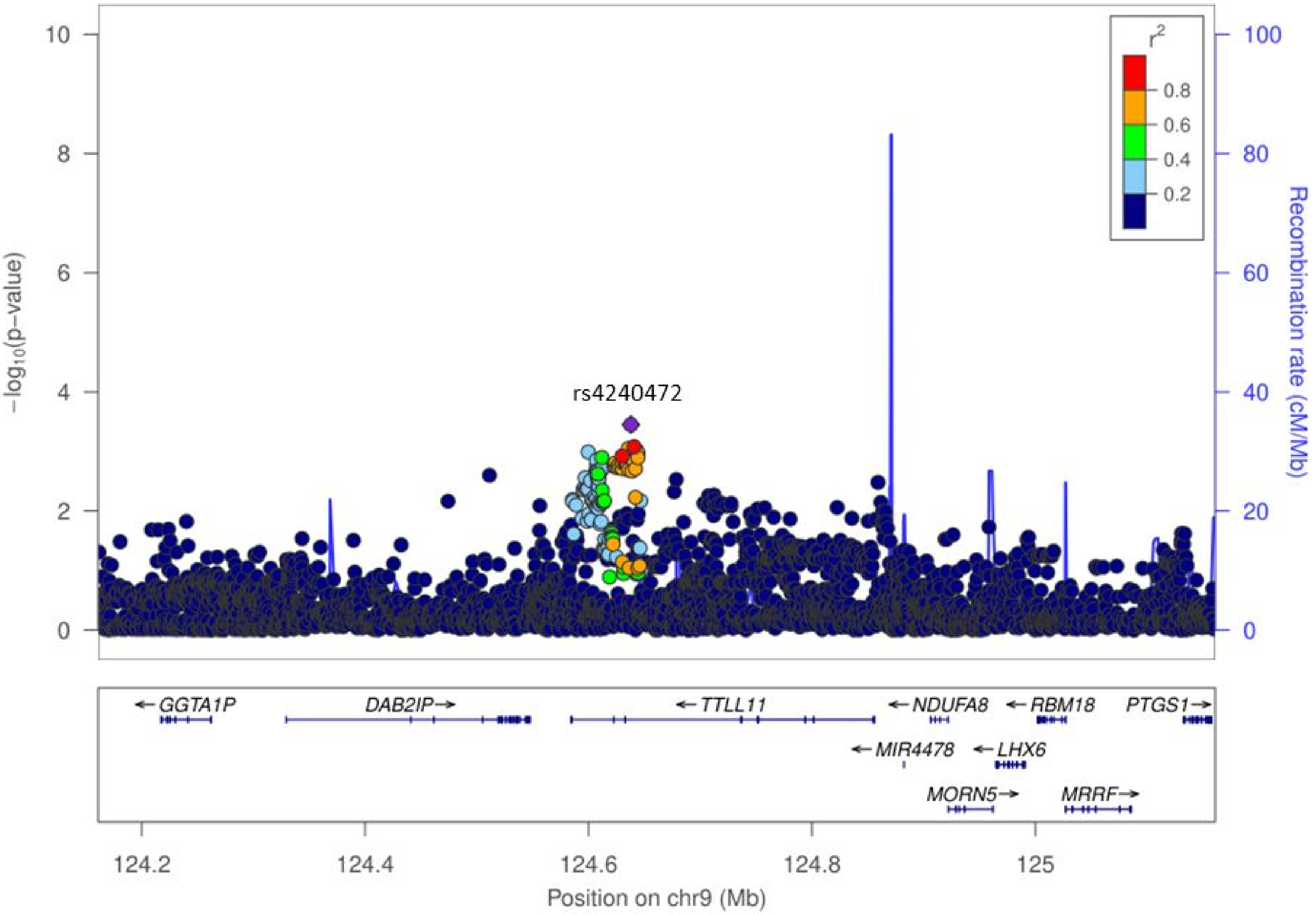

rs4240472
-log10(p-value)
Recombination rate (cM/Mb)
r2
0.8
0.6
0.4
0.2
0
2
4
6
8
10
0
20
40
60
80
100
GGTA1P
DAB2IP
TTLL11
NDUFA8
RBM18
PTGS1
MIR4478
LHX6
MORN5
MRRF
124.2
124.4
124.6
124.8
125
Position on chr9 (Mb)

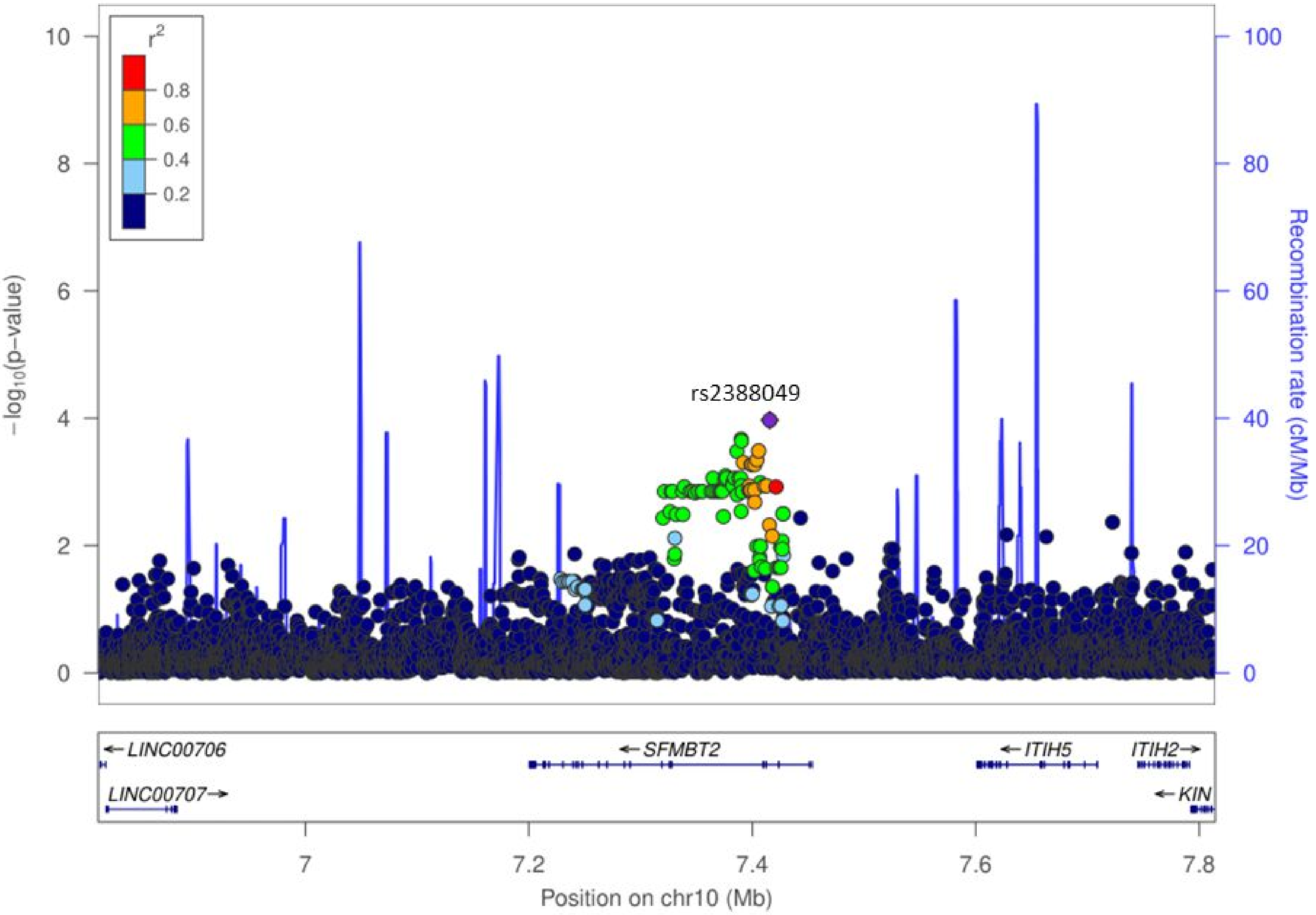

r2
0.8
0.6
0.4
0.2
-log10(p-value)
Recombination rate (cM/Mb)
rs2388049
LINC00706
LINC00707
SFMBT2
ITIH5
ITIH2
KIN
Position on chr10 (Mb)

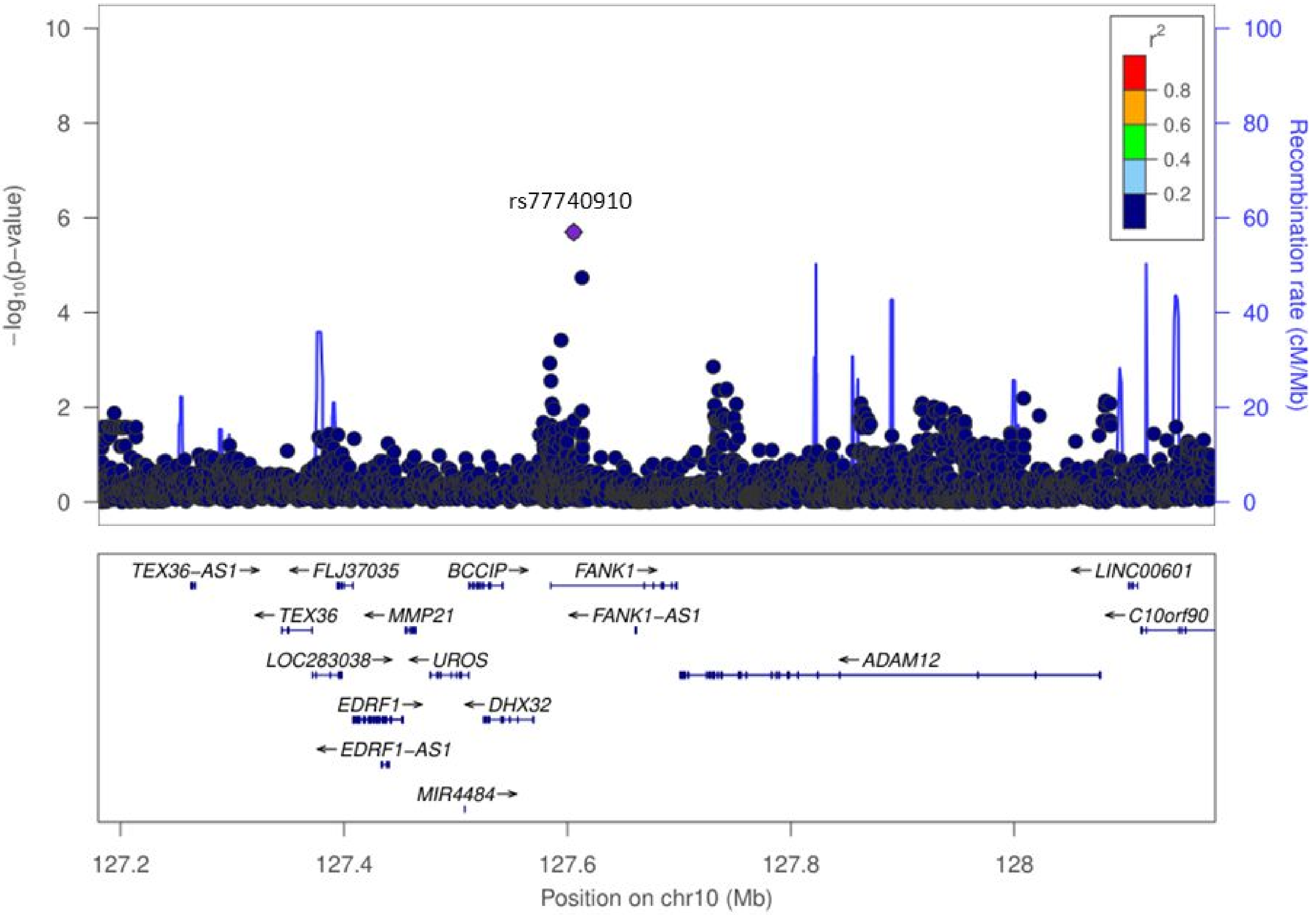

rs77740910
-log10(p-value)
Recombination rate (cM/Mb)
r2
0.8
0.6
0.4
0.2
0
2
4
6
8
10
20
40
60
80
100
TEX36-AS1→
←FLJ37035
BCCIP→
FANK1→
←LINC00601
←TEX36
←MMP21
←FANK1-AS1
←C10orf90
LOC283038→
←UROS
←ADAM12
EDRF1→
←DHX32
←EDRF1-AS1
MIR4484→
127.2
127.4
127.6
127.8
128
Position on chr10 (Mb)

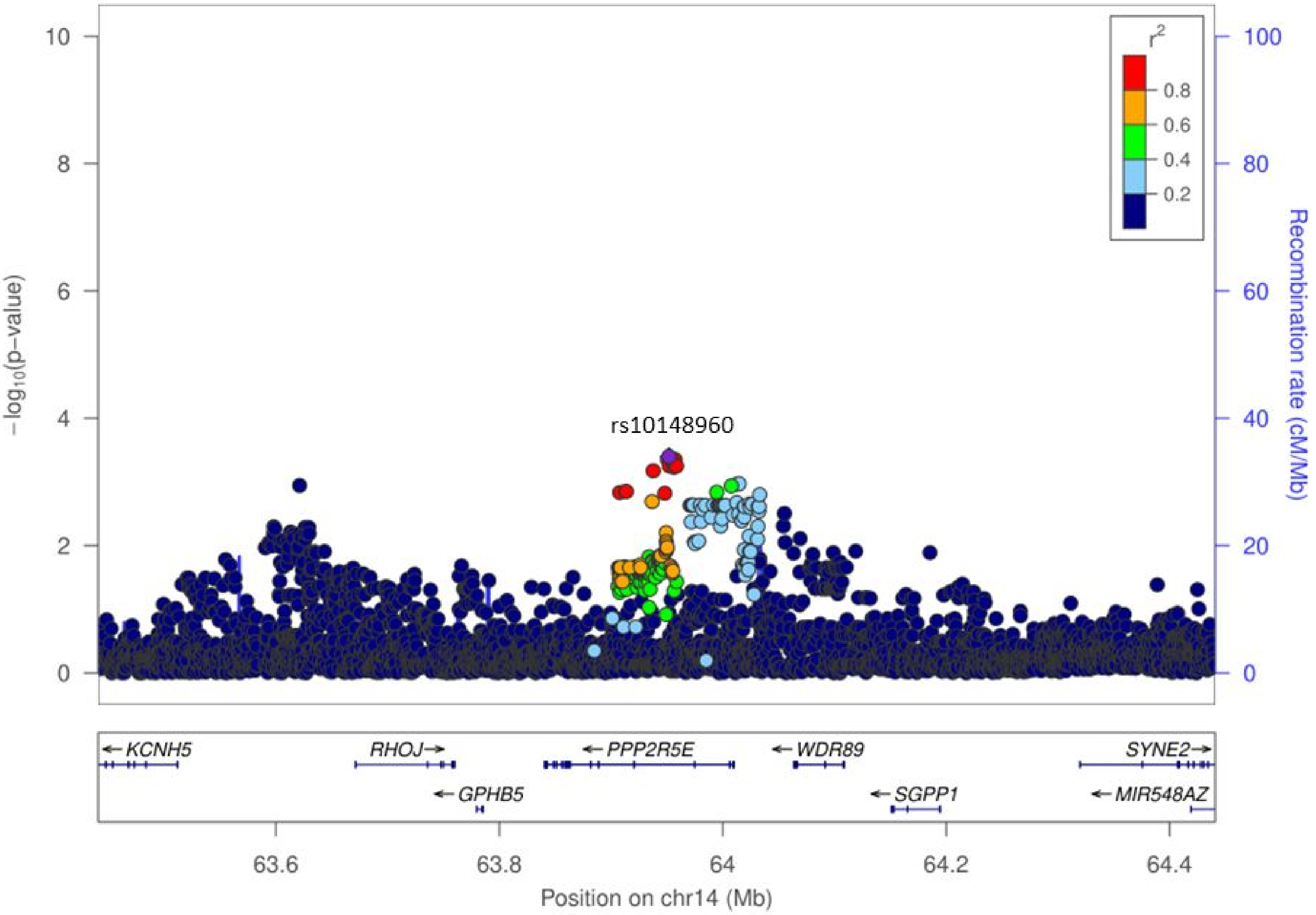

rs10148960
r2
0.8
0.6
0.4
0.2
-log10(p-value)
Recombination rate (cM/Mb)
KCNH5
RHOJ
PPP2R5E
WDR89
SYNE2
GPHB5
SGPP1
MIR548AZ
63.6
63.8
64
64.2
64.4
Position on chr14 (Mb)

Supplemental Figure 2c

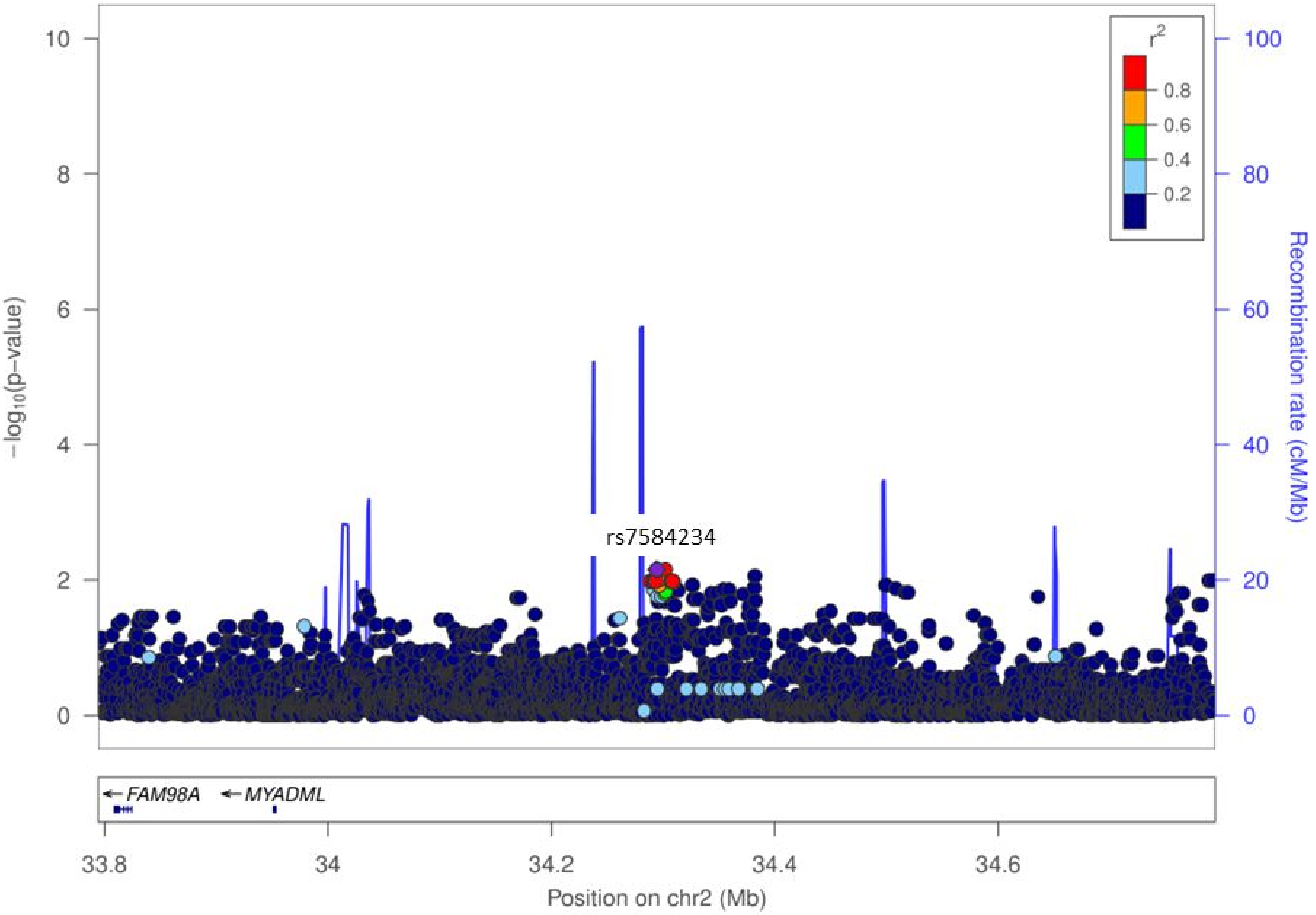

Supplemental Figure 2d

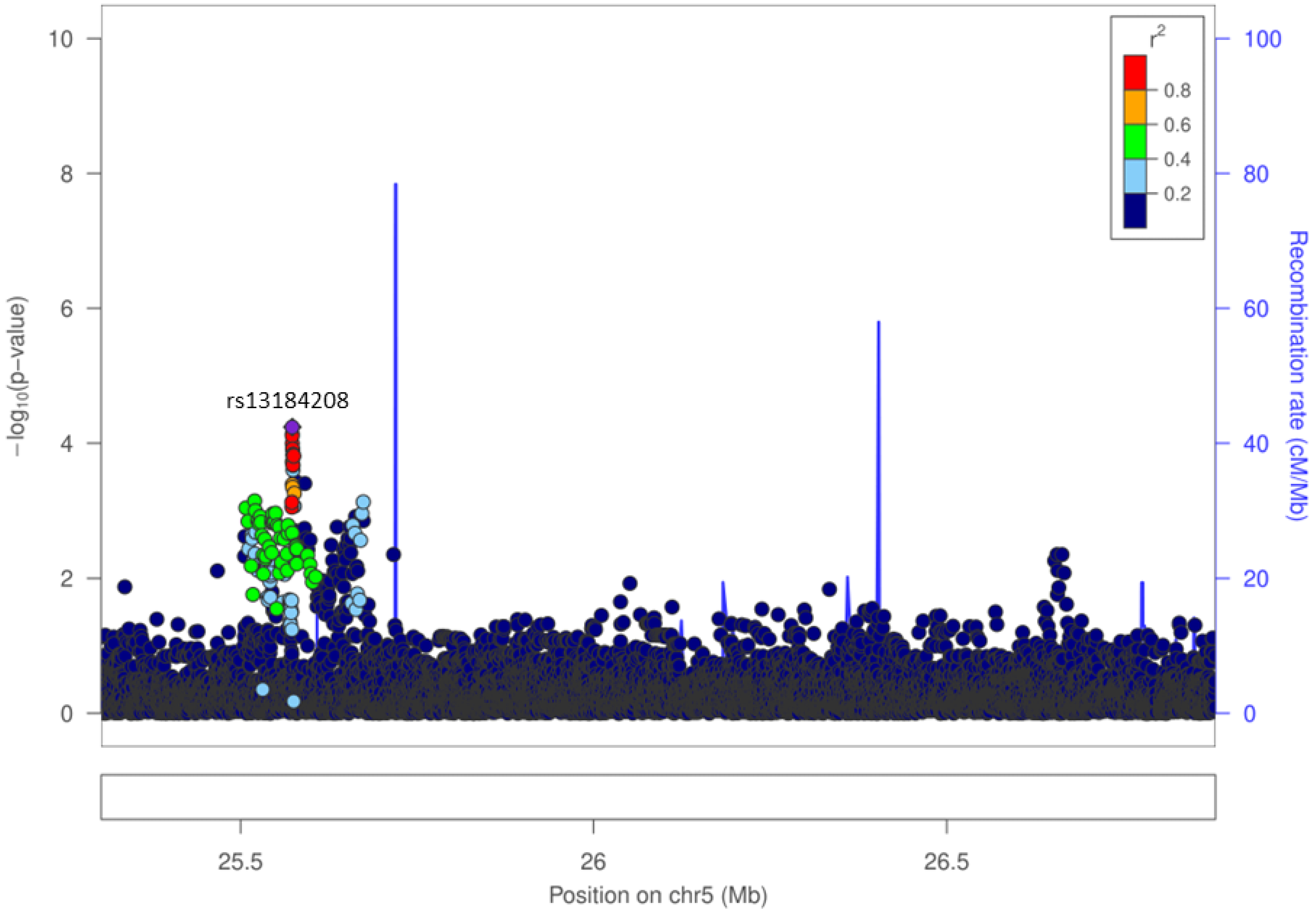

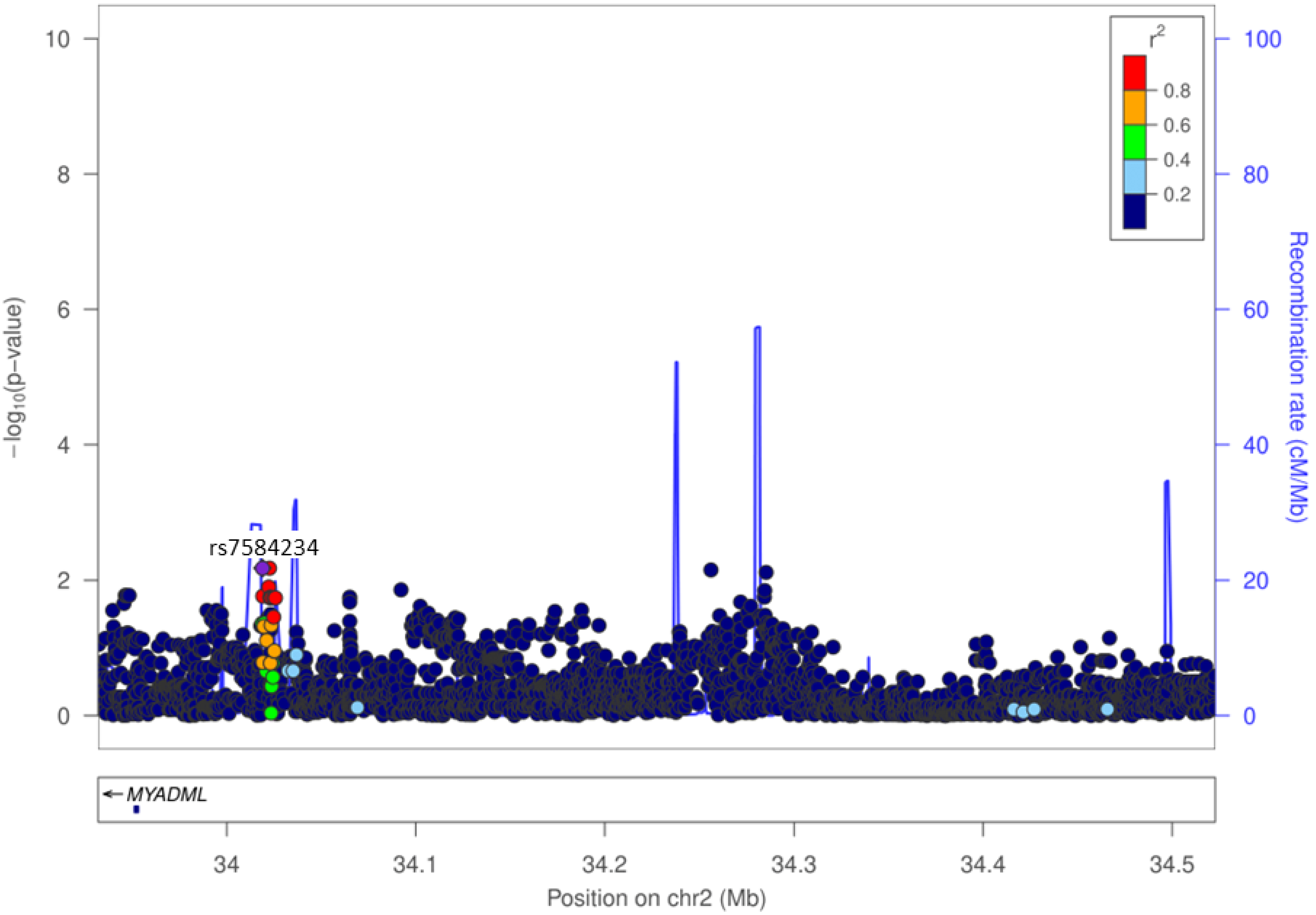
rs7584234
r2
0.8
0.6
0.4
0.2
−log10(p−value)
Recombination rate (cM/Mb)
MYADML
Position on chr2 (Mb)
0
2
4
6
8
10
20
40
60
80
100
34
34.1
34.2
34.3
34.4
34.5

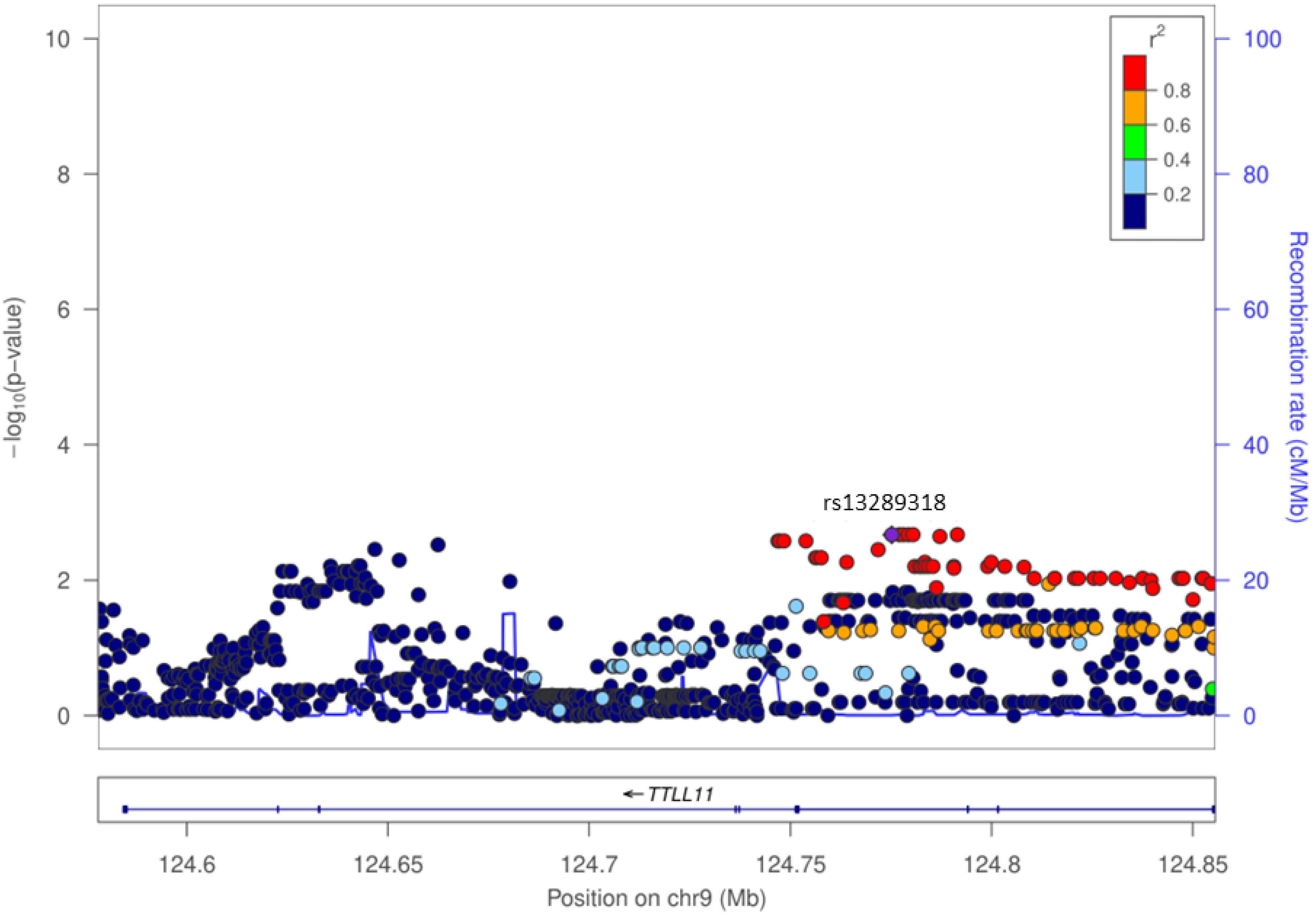

rs13289318
r2
0.8
0.6
0.4
0.2
10
8
6
4
2
0
100
80
60
40
20
0
−log10(p−value)
Recombination rate (cM/Mb)
← TTLL11
124.6
124.65
124.7
124.75
124.8
124.85
Position on chr9 (Mb)

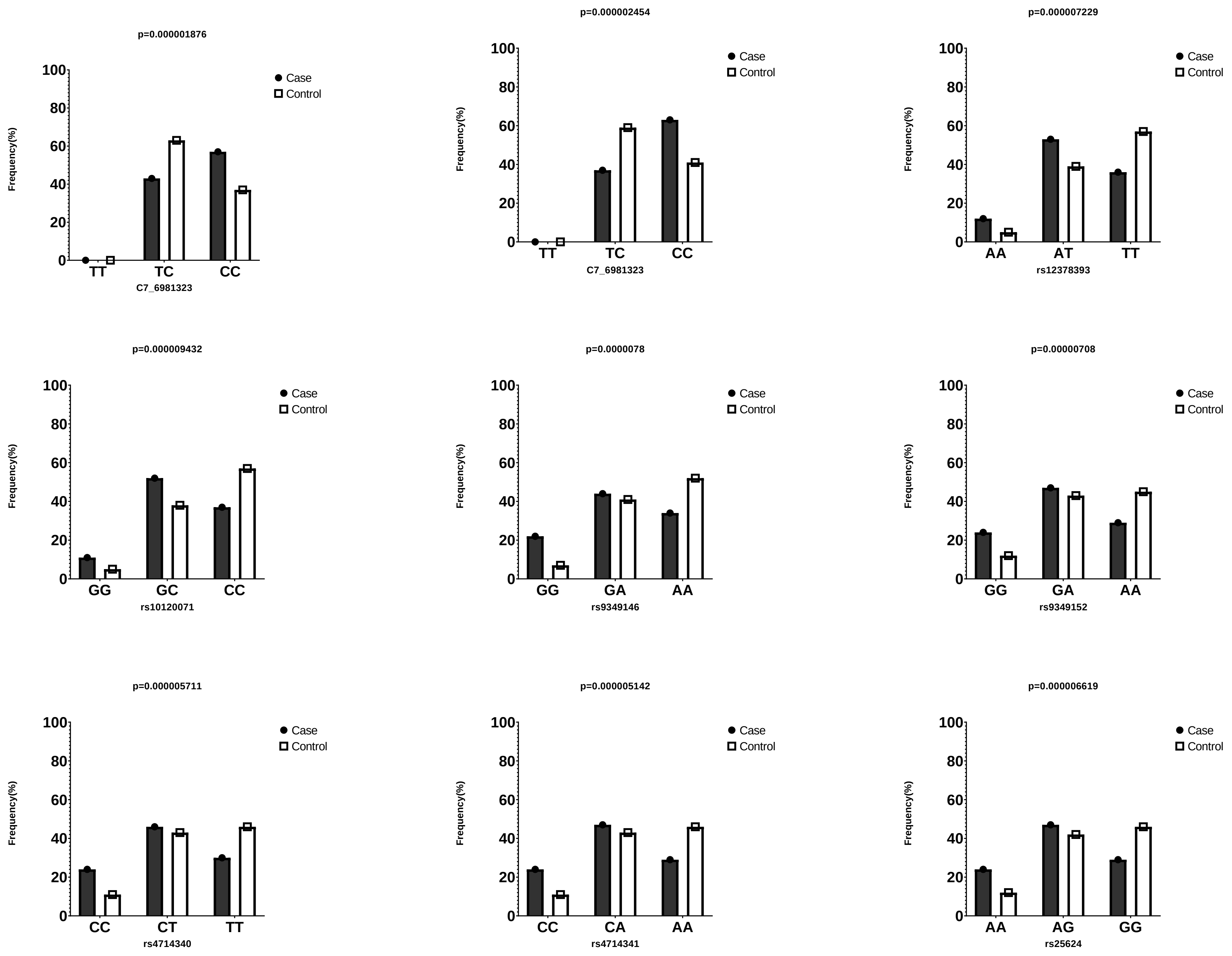
p=0.000001876
Frequency(%)
C7_6981323
TT
TC
CC
Case
Control
p=0.000002454
Frequency(%)
C7_6981323
TT
TC
CC
Case
Control
p=0.000007229
Frequency(%)
rs12378393
AA
AT
TT
Case
Control
p=0.000009432
Frequency(%)
rs10120071
GG
GC
CC
Case
Control
p=0.0000078
Frequency(%)
rs9349146
GG
GA
AA
Case
Control
p=0.00000708
Frequency(%)
rs9349152
GG
GA
AA
Case
Control
p=0.000005711
Frequency(%)
rs4714340
CC
CT
TT
Case
Control
p=0.000005142
Frequency(%)
rs4714341
CC
CA
AA
Case
Control
p=0.000006619
Frequency(%)
rs25624
AA
AG
GG
Case
Control

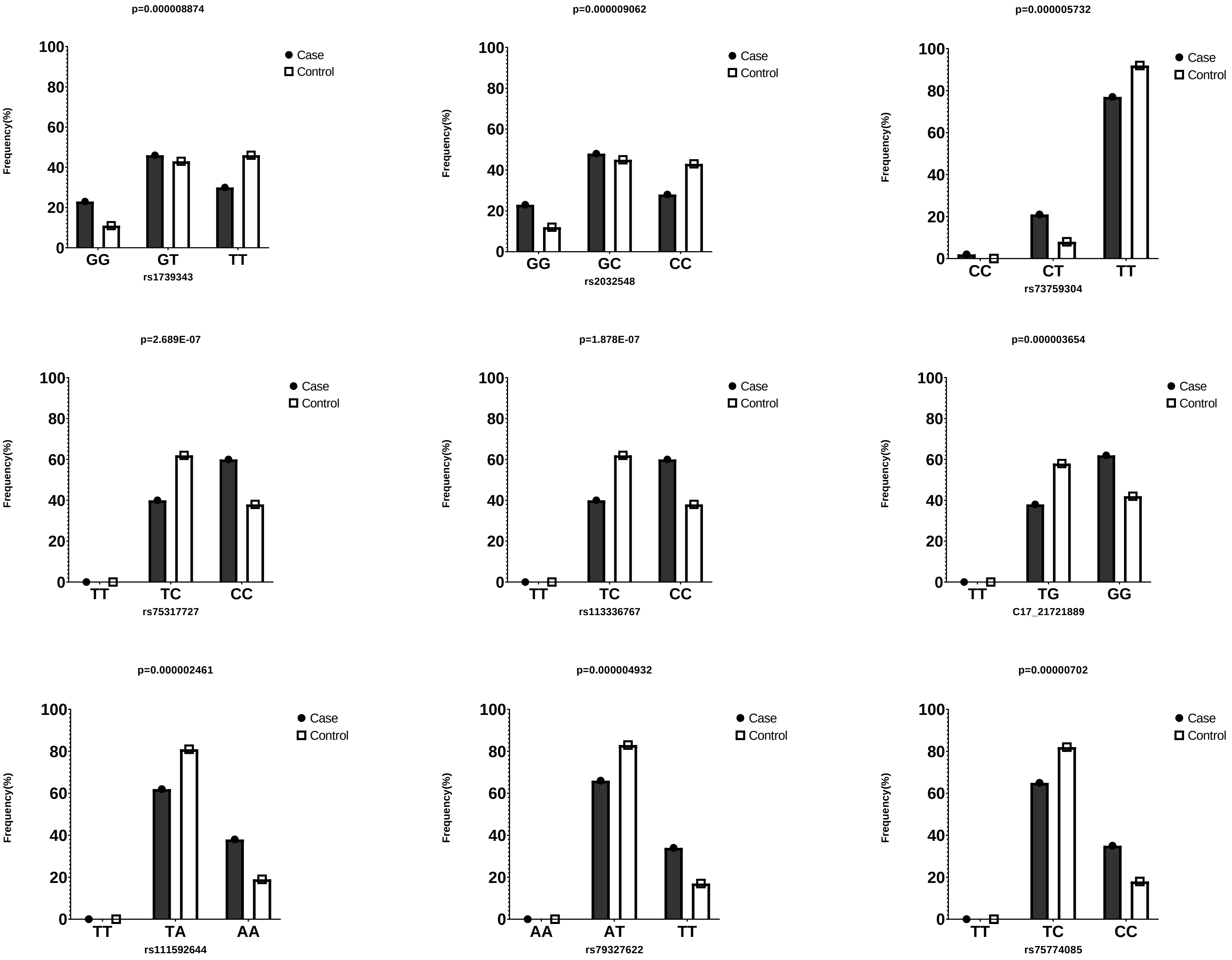
p=0.000008874
Case
Control
Frequency(%)
100
80
60
40
20
0
GG
GT
TT
rs1739343
p=0.000009062
Case
Control
Frequency(%)
GG
GC
CC
rs2032548
p=0.000005732
Case
Control
Frequency(%)
CC
CT
TT
rs73759304
p=2.689E-07
Case
Control
Frequency(%)
TT
TC
CC
rs75317727
p=1.878E-07
Case
Control
Frequency(%)
TT
TC
CC
rs113336767
p=0.000003654
Case
Control
Frequency(%)
TT
TG
GG
C17_21721889
p=0.000002461
Case
Control
Frequency(%)
TT
TA
AA
rs111592644
p=0.000004932
Case
Control
Frequency(%)
AA
AT
TT
rs79327622
p=0.00000702
Case
Control
Frequency(%)
TT
TC
CC
rs75774085

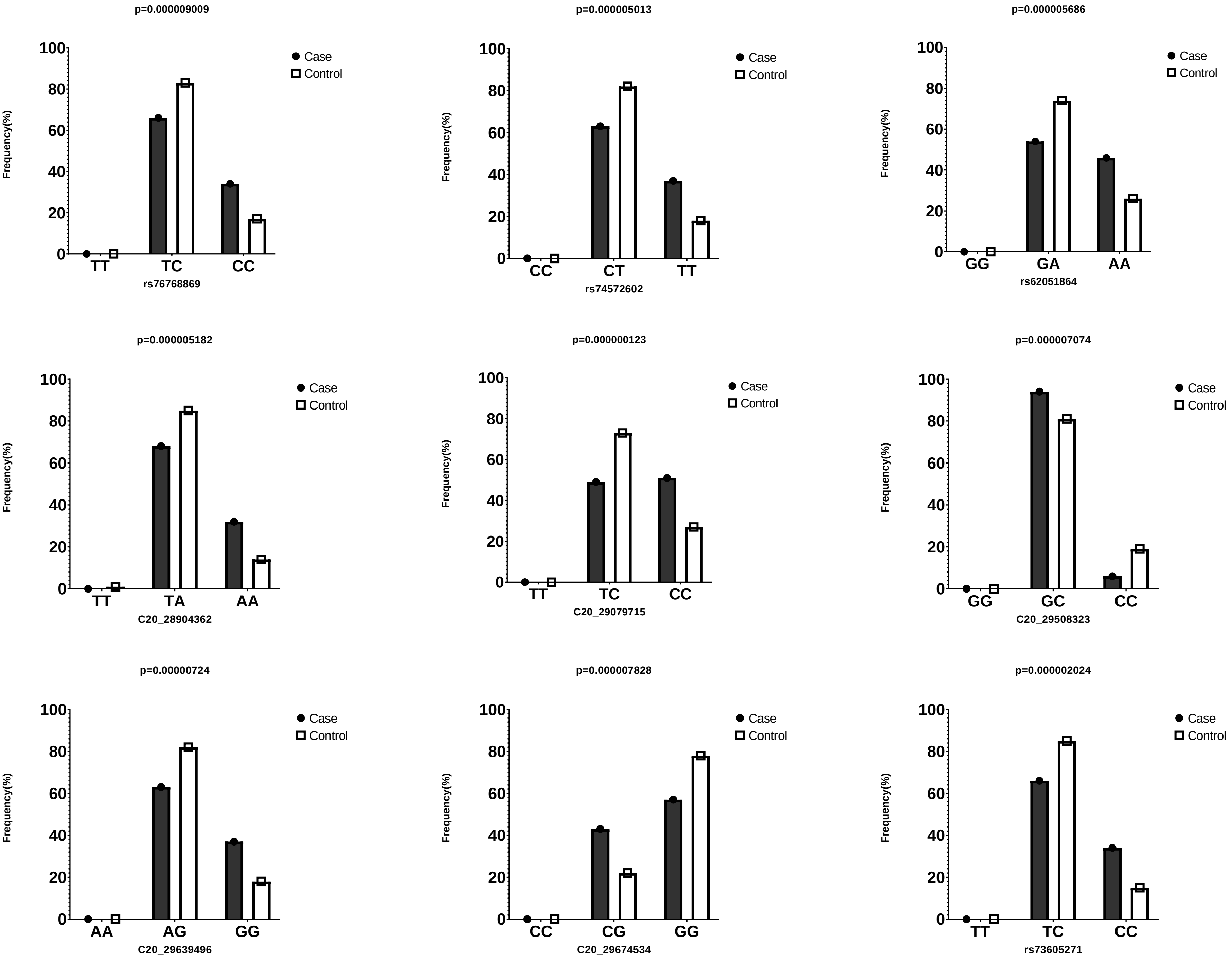
p=0.000009009
Case
Control
Frequency(%)
TT
TC
CC
rs76768869
p=0.000005013
CC
CT
TT
rs74572602
p=0.000005686
GG
GA
AA
rs62051864
p=0.000005182
TT
TA
AA
C20_28904362
p=0.000000123
TT
TC
CC
C20_29079715
p=0.000007074
GG
GC
CC
C20_29508323
p=0.00000724
AA
AG
GG
C20_29639496
p=0.000007828
CC
CG
GG
C20_29674534
p=0.000002024
TT
TC
CC
rs73605271

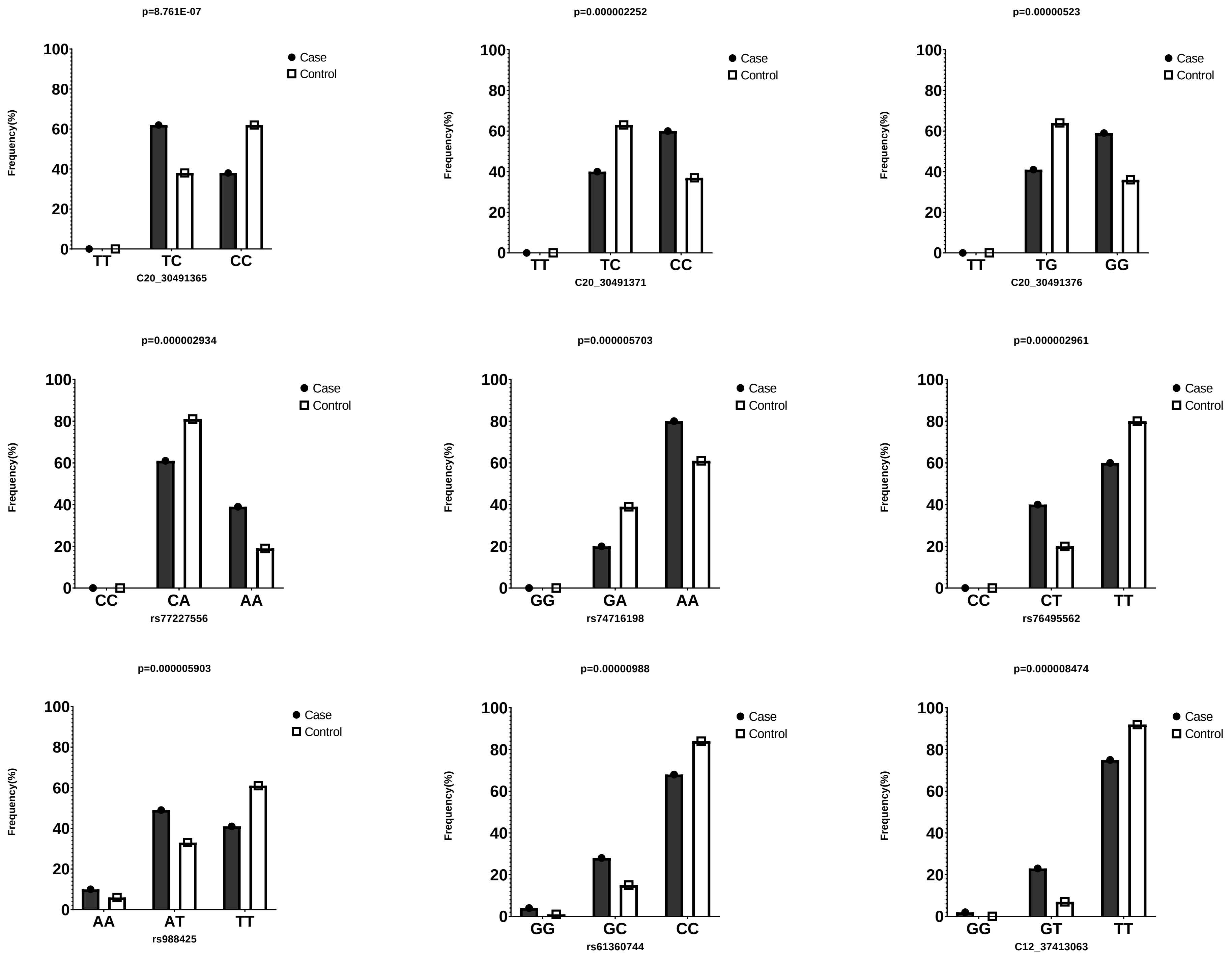
p=8.761E-07
Case
Control
Frequency(%)
100
80
60
40
20
0
TT
TC
CC
C20_30491365
p=0.000002252
Case
Control
Frequency(%)
TT
TC
CC
C20_30491371
p=0.00000523
Case
Control
Frequency(%)
TT
TG
GG
C20_30491376
p=0.000002934
Case
Control
Frequency(%)
CC
CA
AA
rs77227556
p=0.000005703
Case
Control
Frequency(%)
GG
GA
AA
rs74716198
p=0.000002961
Case
Control
Frequency(%)
CC
CT
TT
rs76495562
p=0.000005903
Case
Control
Frequency(%)
AA
AT
TT
rs988425
p=0.00000988
Case
Control
Frequency(%)
GG
GC
CC
rs61360744
p=0.000008474
Case
Control
Frequency(%)
GG
GT
TT
C12_37413063

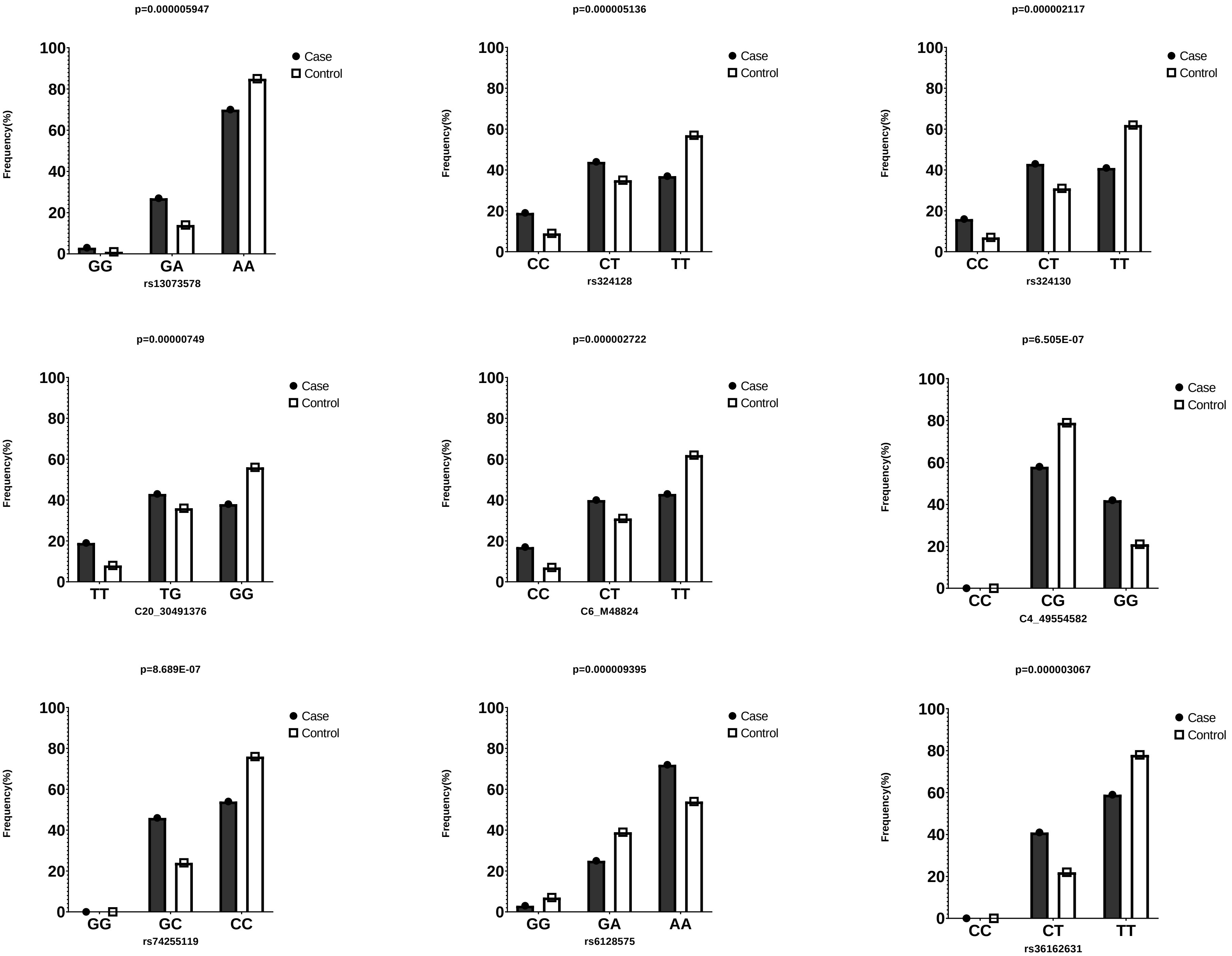

p=0.000005947
Frequency(%)
Case
Control
GG
GA
AA
rs13073578
p=0.000005136
Frequency(%)
Case
Control
CC
CT
TT
rs324128
p=0.000002117
Frequency(%)
Case
Control
CC
CT
TT
rs324130
p=0.00000749
Frequency(%)
Case
Control
TT
TG
GG
C20_30491376
p=0.000002722
Frequency(%)
Case
Control
CC
CT
TT
C6_M48824
p=6.505E-07
Frequency(%)
Case
Control
CC
CG
GG
C4_49554582
p=8.689E-07
Frequency(%)
Case
Control
GG
GC
CC
rs74255119
p=0.000009395
Frequency(%)
Case
Control
GG
GA
AA
rs6128575
p=0.000003067
Frequency(%)
Case
Control
CC
CT
TT
rs36162631

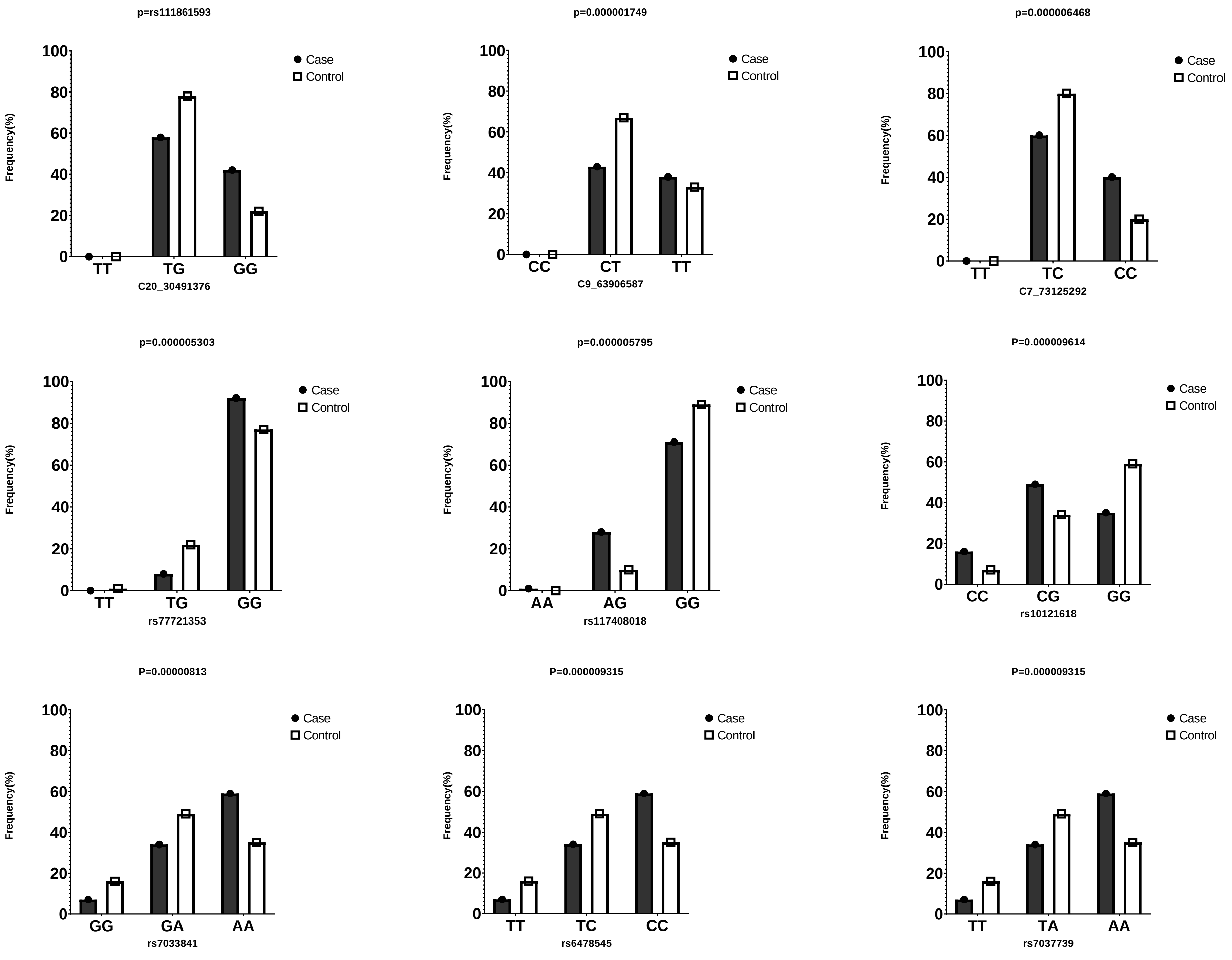
p=rs111861593
Frequency(%)
100
80
60
40
20
0
TT
TG
GG
C20_30491376
Case
Control
p=0.000001749
Frequency(%)
CC
CT
TT
C9_63906587
Case
Control
p=0.000006468
Frequency(%)
TT
TC
CC
C7_73125292
Case
Control
p=0.000005303
Frequency(%)
TT
TG
GG
rs77721353
Case
Control
p=0.000005795
Frequency(%)
AA
AG
GG
rs117408018
Case
Control
P=0.000009614
Frequency(%)
CC
CG
GG
rs10121618
Case
Control
P=0.00000813
Frequency(%)
GG
GA
AA
rs7033841
Case
Control
P=0.000009315
Frequency(%)
TT
TC
CC
rs6478545
Case
Control
P=0.000009315
Frequency(%)
TT
TA
AA
rs7037739
Case
Control

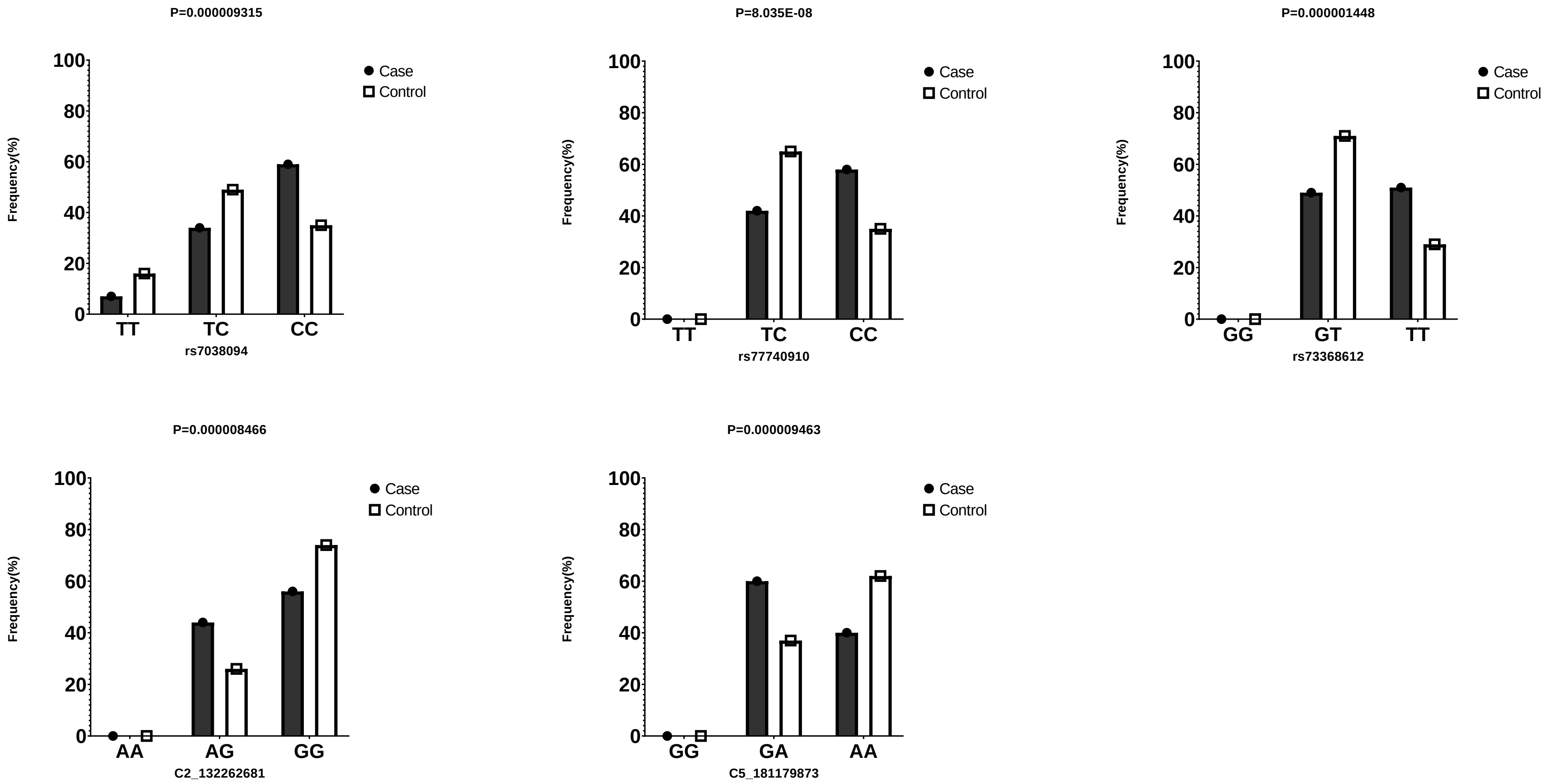
P=0.000009315
Frequency(%)
100
80
60
40
20
0
TT
TC
CC
rs7038094
Case
Control
P=8.035E-08
Frequency(%)
TT
TC
CC
rs77740910
Case
Control
P=0.000001448
Frequency(%)
GG
GT
TT
rs73368612
Case
Control
P=0.000008466
Frequency(%)
AA
AG
GG
C2_132262681
Case
Control
P=0.000009463
Frequency(%)
GG
GA
AA
C5_181179873
Case
Control

Supplemental Figure 4a

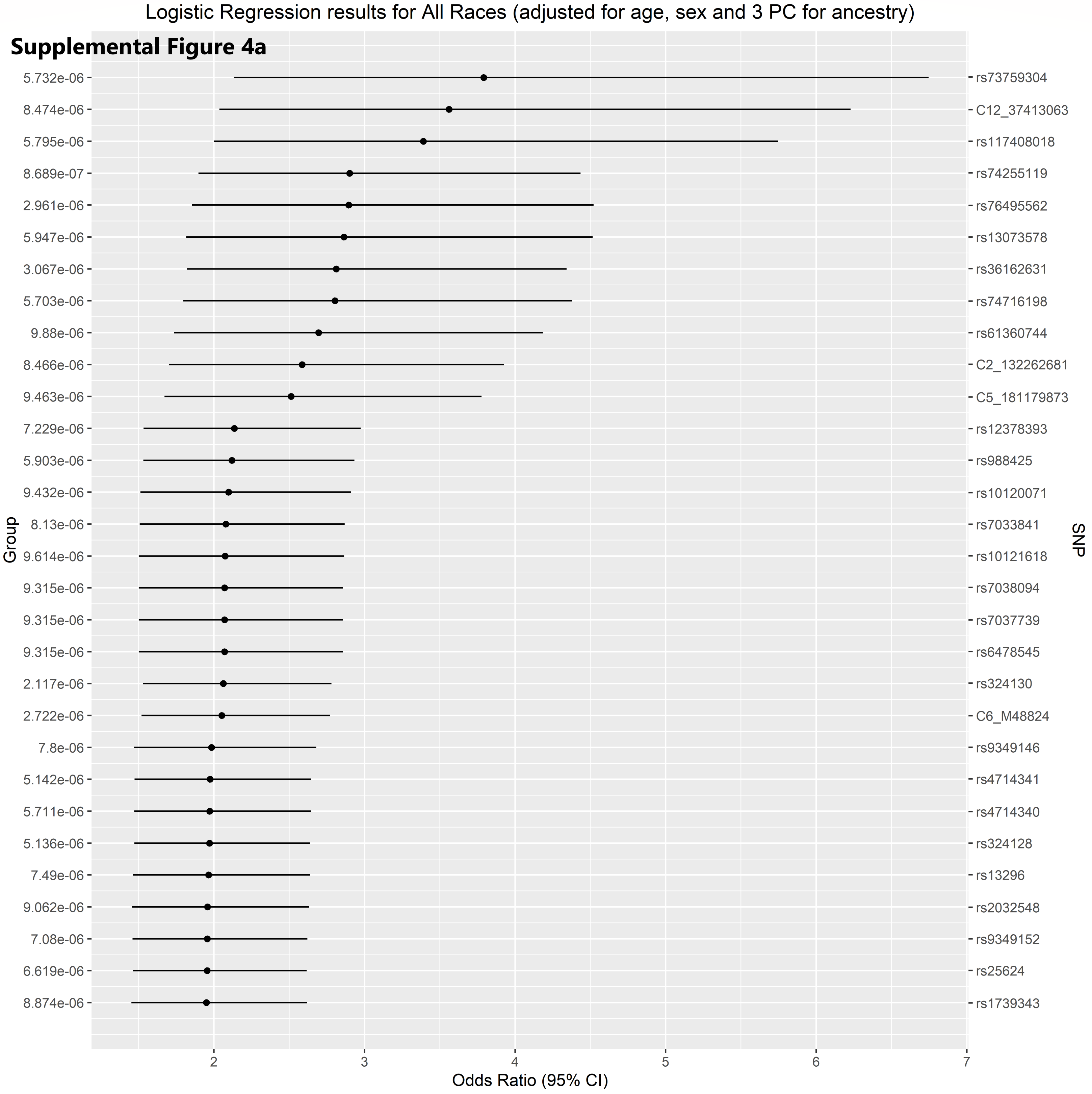

Supplemental Figure 4b

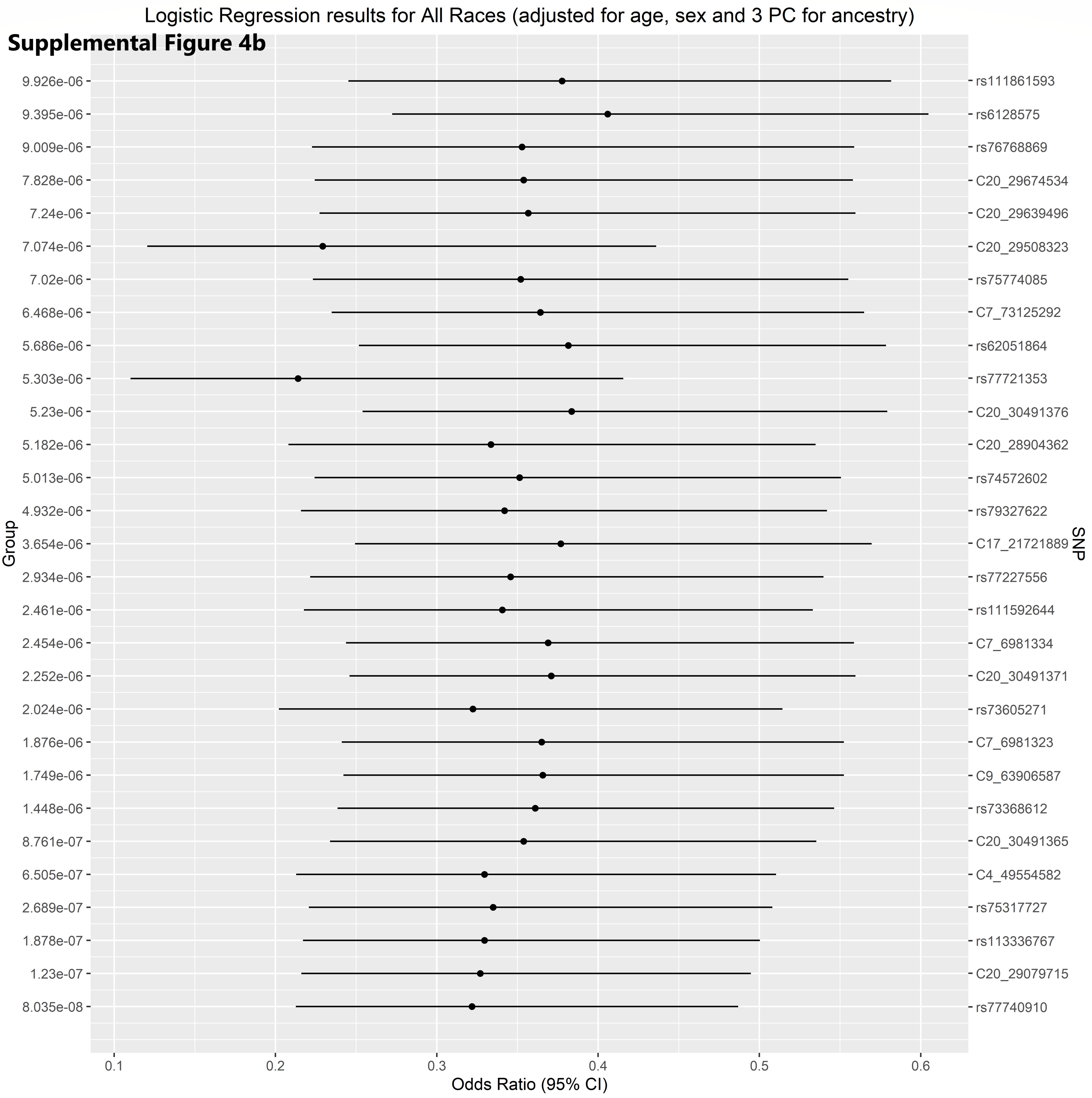

Supplemental Figure 5a

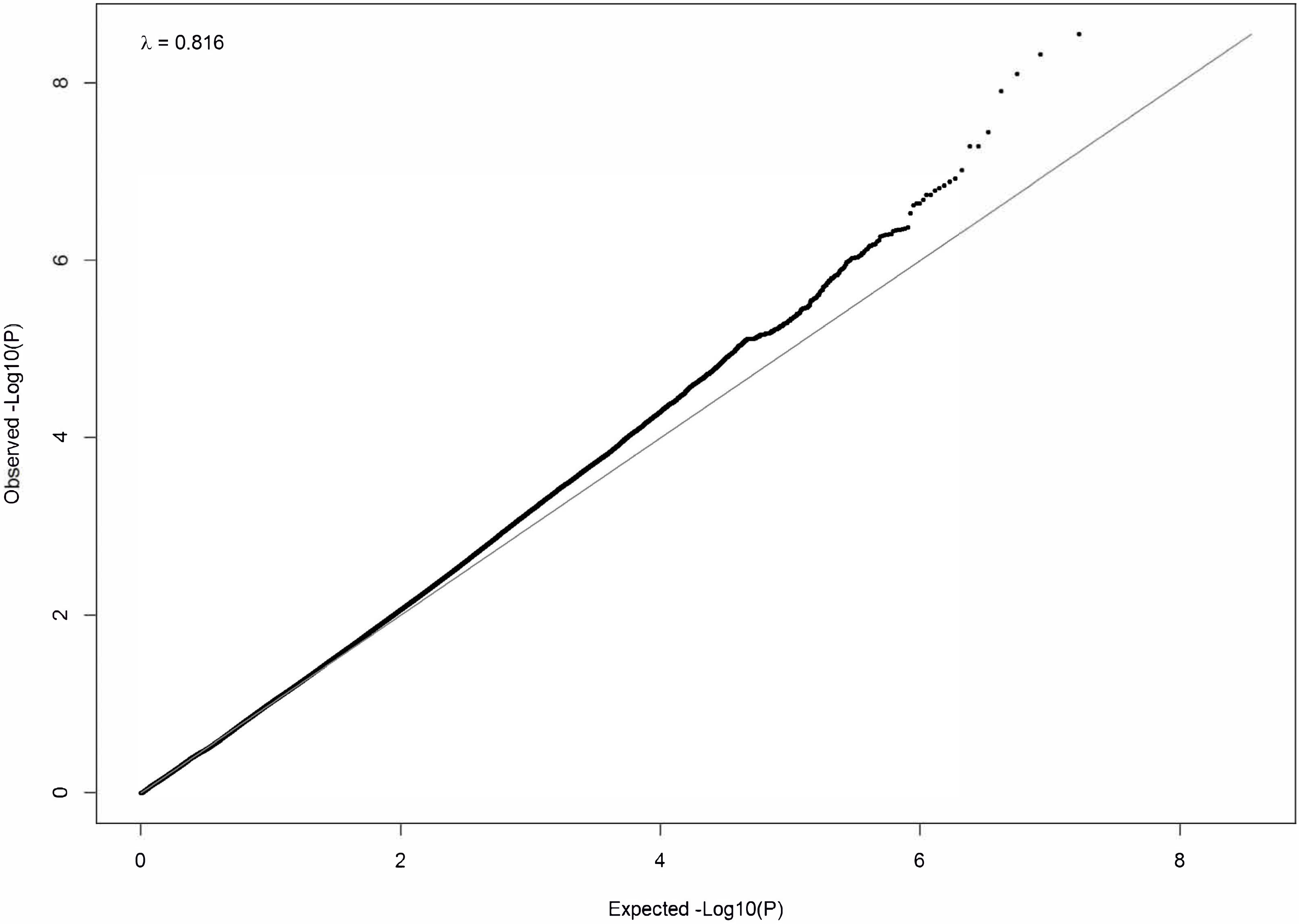

Supplemental Figure 5b

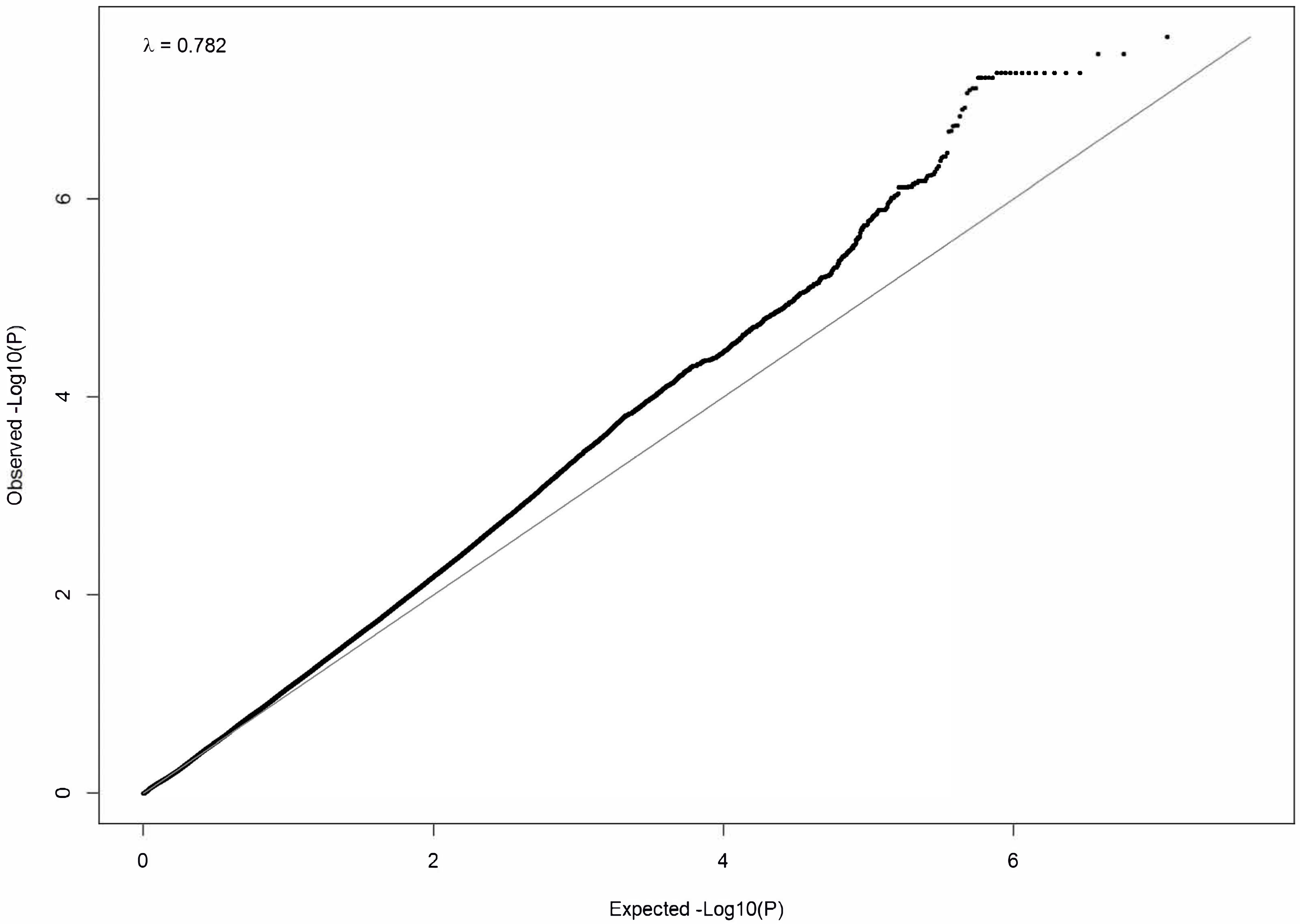

Supplemental Figure 5c

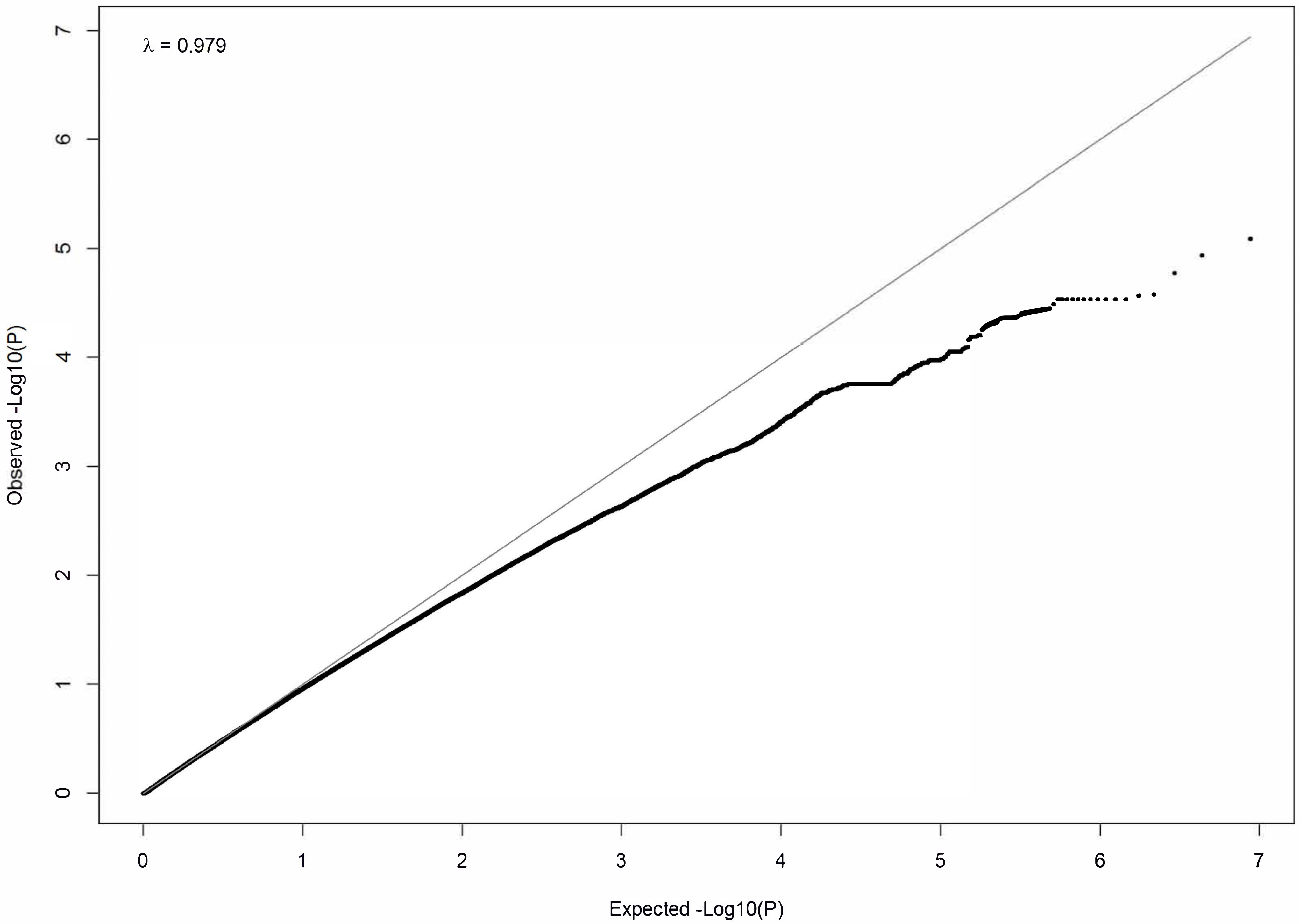

Supplemental Figure 5d

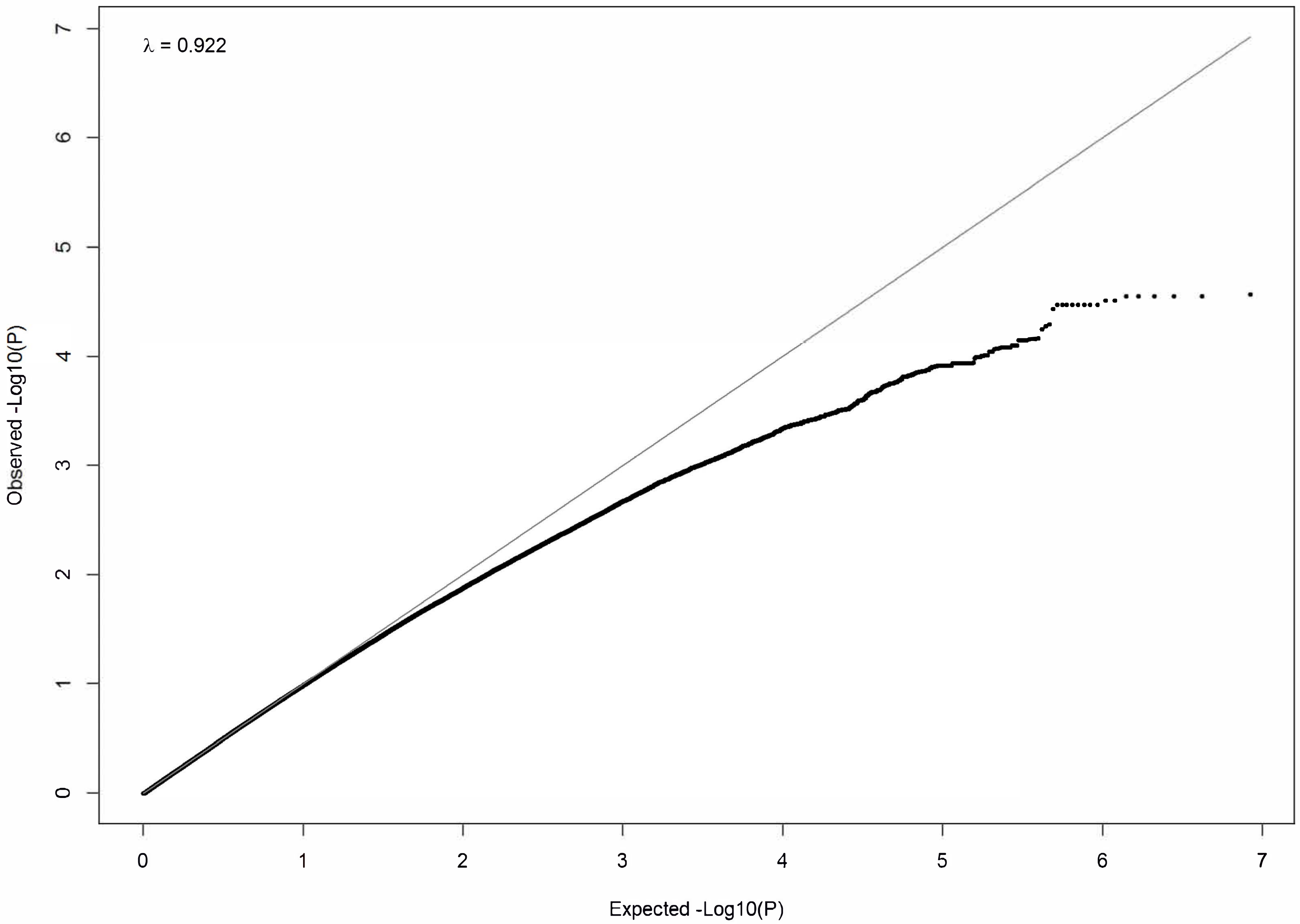

Supplement: Supplementary Figure 1 — Principal Component Analysis Plot (PC1 vs PC2 vs PC3) for ancestry inference. [file DataSheet_3.pdf]
