# Supplementary material for: Pharmacogenomics of intravenous immunoglobulin response in Kawasaki disease
Source: Front Immunol. 2024 Jan 8;14:1287094. doi: 10.3389/fimmu.2023.1287094 (PMC10800400; doi:10.3389/fimmu.2023.1287094)
Supplement: Supplementary Table 1 — (A) Location of all SNPs with both reference and alternate variants observed in KD participants. (B) Functional SNPs observed in KD Participants. [file DataSheet_2.pdf]

Supplementary Table 1a. Location of all SNPS with both reference and alternate variants observed in KD participants

Function	Count
downstream	283322
exonic	390744
exonic;splicing	188
intergenic	23660970
intronic	16764594
ncRNA_exonic	165824
ncRNA_exonic;splicing	58
ncRNA_intronic	2862772
ncRNA_splicing	976
ncRNA_UTR5	6
splicing	2948
upstream	278638
upstream;downstream	11684
UTR3	490400
UTR5	92878
UTR5;UTR3	266
	43288336

Supplementary Table 1b. Functional SNPs observed in KD Participants

Exonic	Function	Count
exonic	.	556
exonic	frameshift	8250
exonic	nonframeshift	8
exonic	nonsynonymous	216966
exonic	stopgain	1312
exonic	stoploss	530
exonic	synonymous	161094
exonic	unknown	2028
exonic;splicing	.	2
exonic;splicing	frameshift	12
exonic;splicing	nonsynonymous	104
exonic;splicing	synonymous	68
exonic;splicing	unknown	2
ncRNA_exonic	.	165824
ncRNA_exonic;splicing	.	58
		556814

Supplementary Table 2. Number of SNPs, Indels, Private SNPs and transversion (Ts/Tv) ratio and mean depth coverage in each KD participant.

ID	SNPs	Indels	Private SNPs	Transitions	Transversions	ts/tv	MEAN_DEPTH
1	3782963	827938	24822	2477257	1206772	2.05	30.6072
2	3794334	834819	35400	2480636	1213044	2.04	29.8954
3	3852594	847918	28367	2518373	1230661	2.05	30.6903
4	3884731	853059	33102	2539939	1240422	2.05	30.6718
5	3798581	836985	42894	2481269	1214028	2.04	31.7613
6	3822082	836985	824	2501245	1220124	2.05	28.919
7	3829217	845128	21946	2504310	1220302	2.05	30.3258
8	3759974	807365	707	2463914	1202523	2.05	30.7497
9	3755340	821863	23458	2459431	1199276	2.05	28.7848
10	3903386	853092	33616	2554362	1246263	2.05	30.7669
11	4346753	933671	67154	2847101	1388820	2.05	30.7492
12	3916713	854577	35948	2564588	1250612	2.05	30.6154
13	3956199	870035	505	2583953	1264582	2.04	31.0457
14	3791705	835332	981	2479676	1208428	2.05	29.2022
15	3815670	835806	637	2493806	1220292	2.04	21.2373
16	3817000	832308	469	2498875	1218125	2.05	28.6416
17	3852447	834343	828	2522428	1229791	2.05	29.5709
18	3759378	828402	23659	2459996	1198349	2.05	32.7981
19	4068958	882617	44346	2666005	1298359	2.05	29.3118
20	3765588	823203	26681	2465882	1204006	2.05	30.3858
21	3714830	798620	35514	2437761	1191227	2.05	29.6793
22	3775411	831594	45062	2467466	1206294	2.05	30.0448
23	3781957	834401	23335	2473449	1207009	2.05	30.5056
24	3797053	839124	28046	2481341	1214097	2.04	21.3166
25	3803505	826843	27628	2492022	1212898	2.05	26.5729
26	4476336	963840	82337	2929534	1429608	2.05	29.3167
27	3886227	852945	31264	2540900	1241542	2.05	30.5168
28	3757991	829487	24734	2457988	1198531	2.05	21.9799
29	3767649	828349	21963	2464064	1202156	2.05	21.8898
30	3816895	839918	42459	2493882	1220250	2.04	15.0851
31	3805517	840432	24892	2489920	1213203	2.05	29.3916
32	3789654	832251	24160	2480510	1208659	2.05	25.3701
33	3800541	826553	45066	2486367	1215979	2.04	38.0589
34	3773193	831272	25403	2467605	1203531	2.05	29.0509
35	3781993	817437	23179	2477620	1208040	2.05	31.0107
36	3901069	858462	36176	2550260	1244402	2.05	30.1436
37	3793913	830775	28940	2482220	1210516	2.05	29.9708
38	3789508	958903	22147	2474000	1206328	2.05	28.7294
39	3753686	823916	22983	2456712	1197840	2.05	29.1673
40	3781927	830669	23152	2473478	1207490	2.05	30.6761
41	3762126	830130	21602	2461921	1198425	2.05	28.6876
42	3811736	841217	22055	2491501	1216292	2.05	30.7859
43	3791373	827205	43558	2478939	1212668	2.04	27.2393

44	3925093	853983	34428	2572892	1252863	2.05	29.8728
45	4237400	909937	94683	2775736	1353273	2.05	28.3748
46	3855436	842263	39060	2522212	1235513	2.04	29.6822
47	3765345	827461	32747	2462443	1201898	2.05	25.3694
48	3754158	826001	22143	2456473	1197600	2.05	28.4024
49	3762739	829085	21534	2460937	1200091	2.05	29.8556
50	3792760	818648	48063	2482488	1212788	2.05	30.3669
51	3762051	823677	24877	2463834	1200825	2.05	18.8809
52	4526000	967617	89366	2964909	1443439	2.05	30.3771
53	3772364	829761	44123	2465916	1204933	2.05	25.3289
54	3826900	840978	545	2503072	1221345	2.05	30.4585
55	3812726	843364	23102	2493359	1216419	2.05	29.7544
56	3873386	840959	48904	2537256	1236505	2.05	30.5549
57	3902300	856583	27812	2550622	1246103	2.05	28.1688
58	3862500	842039	34443	2528448	1232838	2.05	22.2922
59	3766190	834036	20804	2462579	1200900	2.05	30.4148
60	4044717	882233	40398	2645471	1291272	2.05	30.7106
61	3812659	837931	24018	2494250	1216716	2.05	30.4029
62	3807759	840118	23572	2490482	1214121	2.05	31.0613
63	3922933	853663	33563	2567220	1252053	2.05	24.9657
64	3841003	835737	785	2514472	1225966	2.05	29.6303
65	3840874	846884	27064	2510466	1226383	2.05	30.6458
66	3798776	832940	22538	2486147	1212529	2.05	30.8601
67	4274366	915927	133440	2799410	1366459	2.05	30.9292
68	3754493	820585	21481	2457202	1198463	2.05	29.1479
69	3780190	834774	21977	2471424	1205797	2.05	30.3482
70	3808598	839765	47155	2489249	1217539	2.04	27.6075
71	3897027	849526	34253	2550535	1244185	2.05	30.7134
72	3873387	877959	111908	2509205	1260921	1.99	30.5584
73	3785512	952246	24045	2472956	1206184	2.05	24.5066
74	3968756	870605	41269	2596679	1267528	2.05	30.4962
75	3894655	854676	32562	2547052	1243169	2.05	26.1209
76	3789684	831470	24775	2479806	1209084	2.05	34.0585
77	3810892	832069	35206	2494303	1219109	2.05	23.5218
78	4195356	902733	51716	2748957	1339015	2.05	27.6217
79	3878109	846662	40309	2536400	1238171	2.05	28.3916
80	3840215	840665	32539	2513584	1227068	2.05	30.4482
81	3786674	835071	23431	2477547	1207215	2.05	23.7732
82	3806418	841418	25920	2489389	1213629	2.05	25.9903
83	3795641	837503	26442	2481239	1210468	2.05	26.7289
84	4490983	961234	82274	2940836	1434659	2.05	26.3102
85	3768015	831554	20985	2463438	1202075	2.05	30.9719
86	3787054	832562	22764	2477098	1207817	2.05	30.6536
87	3815613	838279	471	2496810	1218854	2.05	30.7168
88	3801520	835854	22106	2484788	1213590	2.05	30.7802
89	3863870	850732	27696	2526112	1233197	2.05	30.676
90	3784667	825325	43461	2475293	1209753	2.05	27.1443

91	3802789	835953	22335	2488783	1213814	2.05	30.9106
92	3788743	834376	44586	2476098	1210788	2.05	28.7468
93	3787073	833253	33330	2476724	1209880	2.05	29.5132
94	3806369	839873	28774	2488100	1213921	2.05	31.2798
95	3803796	839077	21883	2487085	1213688	2.05	29.4031
96	3841748	844216	30305	2512902	1225867	2.05	28.5518
97	3984430	866738	38107	2609470	1272278	2.05	30.7465
98	3786687	828584	21040	2479601	1208988	2.05	27.8824
99	3796295	829838	22502	2484817	1212624	2.05	28.0283
100	3862551	847842	27468	2525964	1232535	2.05	29.0624
101	3870944	849802	75007	2529904	1237219	2.04	27.4591
102	4463982	953798	85588	2924164	1425050	2.05	30.246
103	3862945	839880	34539	2526873	1234659	2.05	30.8052
104	3781739	832383	21892	2474670	1206605	2.05	26.4423
105	4160574	902567	53506	2724013	1328642	2.05	30.1199
106	3893320	855060	31822	2546060	1241606	2.05	27.6389
107	3936831	864677	35073	2573720	1257698	2.05	30.1842
108	4040996	880462	801	2644449	1290447	2.05	32.7357
109	3859562	969354	26641	2522411	1229912	2.05	31.4426
110	3809222	816042	27593	2500403	1216742	2.05	31.13
111	3788164	829793	45564	2478396	1209639	2.05	27.0532
112	3788558	833752	841	2475821	1210597	2.05	21.2364
113	3815864	839234	25557	2495080	1219071	2.05	31.0984
114	3892018	855577	36288	2544384	1242682	2.05	31.2708
115	3943515	864227	36066	2579259	1260129	2.05	28.6538
116	4450735	958709	79375	2911369	1421668	2.05	30.6179
117	3746613	828731	21311	2448135	1195399	2.05	25.9644
118	4531958	962807	98754	2969229	1447814	2.05	32.0077
119	3772404	825115	23133	2469553	1202368	2.05	29.7572
120	4652031	988783	103770	3045344	1487462	2.05	30.1239
121	3792942	826472	46940	2480767	1212037	2.05	31.4139
122	3857618	848101	34118	2522047	1231429	2.05	30.0154
123	4129082	895377	47238	2703280	1319042	2.05	31.2416
124	3898974	851692	36025	2551667	1244979	2.05	31.8996
125	3732859	822218	33790	2439784	1193125	2.04	25.1113
126	3808623	839705	26901	2491645	1214391	2.05	25.7481
127	3799197	835408	22180	2485316	1212845	2.05	29.6546
128	3911795	858698	33222	2559570	1248881	2.05	29.9425
129	3710313	817015	31764	2425406	1186173	2.04	28.568
130	3867768	854955	28362	2527132	1235168	2.05	32.6825
131	3886632	856679	60450	2539418	1240674	2.05	30.5568
132	3749157	808864	21790	2455584	1197189	2.05	28.8307
133	3812015	832214	31960	2496040	1216679	2.05	24.5166
134	3849743	867965	98517	2496561	1249851	2	28.9262
135	3832003	841077	24620	2505714	1222902	2.05	30.9293
136	3817528	840061	21862	2499477	1217346	2.05	29.7844
137	3767130	824010	22083	2466309	1201867	2.05	29.2419

138	3809962	838567	21198	2492734	1215594	2.05	30.0351
139	3798725	833395	42762	2484400	1213956	2.05	30.6486
140	4296510	918048	122172	2815340	1372874	2.05	26.4384
141	3800313	837390	23332	2486889	1211450	2.05	28.1562
142	3916683	855438	35903	2562812	1251524	2.05	30.6658
143	3830702	845994	41082	2504929	1222189	2.05	29.9647
144	3784587	832449	21318	2475072	1207968	2.05	30.5782
145	3787776	835868	30221	2476915	1207515	2.05	28.2409
146	3857959	844910	32145	2524232	1232774	2.05	26.2515
147	3784899	834017	23308	2474064	1207259	2.05	35.9893
148	3816662	839516	22540	2496910	1218521	2.05	27.7106
149	4533266	969534	92540	2969934	1446804	2.05	28.4947
150	3861131	849289	34944	2525202	1231236	2.05	33.4697
151	3816930	839238	48968	2495509	1217921	2.05	32.7744
152	3816819	836601	31307	2496465	1218101	2.05	32.3815
153	3815737	841426	25356	2495868	1218023	2.05	29.4322
154	4078298	885724	43248	2669507	1302063	2.05	30.6117
155	3872780	849365	34470	2533585	1236167	2.05	30.3108
156	3831402	846755	46412	2502295	1224179	2.04	33.1662
157	3885115	850094	33278	2542961	1240896	2.05	21.6221
158	3830000	839945	27966	2507204	1221756	2.05	31.6214
159	3808884	839695	49571	2489776	1216401	2.05	28.5932
160	3856923	847428	32365	2523412	1229694	2.05	32.3701
161	3873409	849280	32554	2532341	1237524	2.05	31.2418
162	4337649	934802	61718	2839545	1384534	2.05	31.0864
163	3786880	835632	21350	2474276	1208485	2.05	33.5248
164	3859138	839998	32242	2523371	1232635	2.05	33.6926
165	3767750	824691	19945	2463427	1203605	2.05	28.9225
166	3786716	826655	21854	2478740	1209041	2.05	31.5992
167	3817148	834385	22302	2496658	1218971	2.05	35.609
168	4482270	967051	81639	2933688	1430682	2.05	35.5956
169	3806066	838277	24499	2490401	1213743	2.05	30.9402
170	3778998	835266	42845	2468347	1207408	2.04	30.6887
171	3934426	861145	32606	2573523	1256606	2.05	30.6119
172	3819961	821657	50197	2501499	1222429	2.05	27.8497
173	4278953	922821	119441	2801933	1365802	2.05	30.389
174	3808926	827163	23805	2494004	1215705	2.05	30.1646
175	3791154	837478	23550	2477440	1209473	2.05	29.3539
176	4469021	956710	87609	2924417	1427638	2.05	30.7006
177	3830009	841991	28695	2506250	1221389	2.05	30.1055
178	3945379	861095	1048	2580591	1262195	2.04	24.595
179	3995044	864051	39275	2618133	1275027	2.05	30.7605
180	4221746	916414	56747	2761054	1348114	2.05	30.7183
181	3786038	830219	673	2477552	1207294	2.05	30.6804
182	3789561	830455	45616	2478177	1211241	2.05	30.8488
183	3816374	837770	682	2494389	1220568	2.04	30.1073
184	3906616	853382	37201	2556362	1247818	2.05	30.6033

185	3815928	834472	22711	2497783	1217548	2.05	26.884
186	3838952	834231	33089	2514207	1226324	2.05	30.8153
187	3821967	836133	561	2501265	1219748	2.05	30.4054
188	3759709	826159	22370	2461272	1198317	2.05	30.5424
189	3791105	828098	22349	2482308	1209505	2.05	4.18523
190	2822487	555306	21392	1873928	896654	2.09	30.3428
191	3801983	832618	22825	2487874	1213757	2.05	30.9753
192	3854018	841402	33868	2523615	1231781	2.05	30.1563
193	3841084	844990	48319	2510293	1228158	2.04	30.2405
194	3818610	841767	30610	2495968	1218898	2.05	30.5569
195	3804181	834753	24947	2489704	1213235	2.05	30.9549
196	3778446	833680	22254	2470638	1205987	2.05	31.1389
197	3796517	833971	46097	2481808	1213484	2.05	34.1986
198	3856507	846545	491	2523812	1230916	2.05	29.5682
199	3937149	863964	33804	2574264	1257514	2.05	29.0602
200	3738119	774057	23629	2454791	1195037	2.05	38.4086
201	3793985	820566	45824	2484048	1212598	2.05	37.3145
202	4543896	973201	92394	2972277	1451323	2.05	31.5825
203	3787916	837968	21821	2474315	1208395	2.05	30.3267
204	3775382	828871	23308	2471293	1203655	2.05	30.3569
205	3930867	854935	32564	2572358	1255331	2.05	30.6542
206	3820634	837884	26952	2500059	1219402	2.05	30.426
207	3814969	837658	44789	2493769	1220439	2.04	29.7665
208	3795626	832543	31835	2482094	1211589	2.05	26.7577
209	3821745	835563	30077	2502630	1220199	2.05	30.8163
210	3861503	835871	33966	2530665	1233739	2.05	30.5667
211	3833282	845194	26173	2506585	1222384	2.05	30.4672
212	3830321	840925	26093	2506216	1222304	2.05	30.344
213	3814987	837087	22405	2496377	1217084	2.05	29.5203
214	4563173	967876	99349	2990131	1457870	2.05	25.0268
215	3870061	851125	28789	2531831	1235059	2.05	26.0437
216	3783157	833246	26282	2475495	1205947	2.05	25.2909
217	3769500	820451	22648	2470444	1202655	2.05	19.5519
218	3948525	853414	40027	2586408	1262057	2.05	18.1575
219	4277789	903522	65696	2806393	1367584	2.05	21.8599
220	5408374	1759030	2093686	3078126	2206207	1.4	25.9772
221	3816745	835350	26005	2498799	1219481	2.05	23.2686
222	3774909	802206	162136	2446552	1246092	1.96	15.0555
223	3747131	803558	23012	2457661	1196969	2.05	19.4605
224	3858075	841709	35457	2526123	1231784	2.05	24.0387
225	4569803	956885	101146	3000515	1460255	2.05	19.3862
226	3763734	814592	48461	2465467	1203853	2.05	19.1073
227	3730677	807928	22632	2445325	1191775	2.05	19.46
228	3774058	828023	24833	2469355	1205184	2.05	25.2162
229	3819534	833366	32210	2502665	1219288	2.05	26.9666
230	3735304	826570	22287	2444972	1190620	2.05	27.4965
231	3893755	847669	34396	2552164	1242950	2.05	27.2854

232	3800671	838743	47998	2483935	1214554	2.05	27.3227
233	3792620	838410	48157	2477962	1211867	2.04	30.9287
234	3817421	830607	55705	2501458	1219111	2.05	27.075
235	3771387	826194	23031	2468765	1203934	2.05	24.3528
236	3800436	882978	30589	2490881	1211696	2.06	24.87
237	3895430	846052	36368	2551472	1244579	2.05	26.6132
238	3844474	845539	30365	2513832	1227626	2.05	26.8568
239	3796030	836062	22855	2484799	1210761	2.05	26.7058
240	3793891	821956	22504	2486729	1209901	2.06	26.0497
241	3741670	790029	24140	2455914	1195130	2.05	26.2225
242	3795567	819937	44331	2483349	1214700	2.04	30.0792
243	3791527	837772	43362	2477488	1211344	2.05	25.9377
244	3803839	840225	32604	2485997	1215181	2.05	30.8294
245	3747318	821807	21714	2452811	1196173	2.05	26.6324
246	3811197	831986	23888	2496930	1217001	2.05	23.3169
247	3772481	828972	24671	2469591	1203109	2.05	20.9649
248	3775435	827936	22487	2471281	1204404	2.05	28.6459
249	3767371	817636	25823	2468543	1202647	2.05	23.8139
250	3854783	846090	34238	2523581	1230707	2.05	26.7013
251	3757941	822821	22276	2462001	1198051	2.06	26.6704
252	3935048	856544	35148	2576562	1256404	2.05	26.4909
253	3799948	828933	25383	2489355	1212645	2.05	26.5216
254	3780883	832746	23627	2472943	1207046	2.05	25.4584
255	3765358	824995	21746	2465571	1202399	2.05	26.7682
256	3856337	846668	36837	2521409	1232776	2.05	26.9057
257	3800803	833531	22754	2488747	1213362	2.05	26.7544
258	3702920	815507	29898	2420496	1183463	2.05	26.5603
259	3823076	840122	28202	2502752	1219425	2.05	26.7134
260	3805584	834821	22504	2491938	1214513	2.05	25.1583
261	3825490	834896	48112	2503766	1221780	2.05	26.3858
262	4210076	912059	60272	2758280	1344691	2.05	26.7156
263	3892602	850911	33986	2548089	1242490	2.05	26.6407
264	3877181	852197	28429	2539090	1237071	2.05	26.7492
265	3756278	823687	23802	2459267	1199002	2.05	23.952
266	3814950	835538	22333	2498457	1217052	2.05	24.8773
267	3776776	797539	44480	2478801	1206949	2.05	26.3189
268	3790373	833759	44857	2481016	1211073	2.05	26.698
269	3764404	817409	24585	2468527	1200872	2.06	26.5658
270	4242150	908282	57456	2780448	1355521	2.05	26.51
271	3808291	837809	28959	2491432	1214734	2.05	28.3236
272	3780066	837453	21350	2470722	1205191	2.05	30.8537
273	3866057	849952	29479	2528351	1233686	2.05	26.8684
274	3759372	830265	21667	2460267	1198484	2.05	26.9221
275	3790039	834701	28153	2479471	1209602	2.05	23.7919
276	4257027	913725	56098	2788771	1360345	2.05	26.5801
277	3753997	821076	31247	2454613	1200617	2.04	26.5412
278	3878429	847351	34957	2541119	1237259	2.05	25.9772

279	3809050	831498	36176	2491783	1217380	2.05	30.1989
280	3776981	822758	22970	2474674	1204342	2.05	29.194
281	3797143	820322	23385	2488621	1211913	2.05	25.3572
282	3801791	837274	23173	2486903	1213481	2.05	26.7639
283	3824747	843488	44127	2500492	1221470	2.05	28.0511
284	3921054	856641	83093	2566327	1252383	2.05	26.5779
285	3776332	827504	47283	2472280	1207428	2.05	24.6943
286	3775483	831722	21812	2469603	1204714	2.05	26.8719
287	3770224	822371	29226	2468981	1205161	2.05	26.5642
288	3792449	835257	22944	2482650	1209964	2.05	25.8788
289	3761827	826387	22080	2462664	1200494	2.05	26.8705
290	3872726	849762	37843	2534019	1238034	2.05	22.5618
291	3749812	818871	43751	2456014	1198912	2.05	25.1015
292	3809825	831732	23536	2496021	1216186	2.05	26.5577
293	3792844	834505	23327	2482998	1209925	2.05	24.6494
294	4007443	876349	45021	2622991	1280020	2.05	26.8367
295	3742076	808695	22298	2453455	1194663	2.05	27.7538
296	3776507	830300	21413	2471486	1204371	2.05	27.8521
297	3913773	857973	37974	2561808	1249523	2.05	26.6571
298	3782255	792132	24890	2481191	1208084	2.05	31.4875
299	3998984	866710	43026	2621043	1275586	2.05	26.5193
300	4160875	903419	47621	2724310	1328522	2.05	26.6728
301	3770611	824904	42940	2467750	1205360	2.05	26.4252
302	3741180	815595	22998	2451442	1194468	2.05	18.9888
303	3772590	833748	21577	2466616	1203266	2.05	26.8553
304	3904541	852756	36075	2555605	1247195	2.05	26.5461
305	3884847	852600	39200	2540986	1241373	2.05	25.45
306	3773567	824916	22391	2470391	1204977	2.05	26.668
307	3813632	841478	43455	2493940	1217574	2.05	26.9849
308	3757441	823064	21766	2461277	1197947	2.05	23.0664
309	3783900	833508	23635	2473559	1209279	2.05	26.6378
310	3789584	823092	24163	2483399	1209300	2.05	26.67
311	3767022	827069	21819	2465371	1202126	2.05	24.945
312	4102695	891244	45295	2687791	1310003	2.05	24.5798
313	3752354	808526	22450	2460707	1198125	2.05	25.779
314	3869654	830753	34005	2537928	1235548	2.05	25.2242
315	3788995	830138	23473	2482160	1209607	2.05	25.0371
316	3759088	823194	23183	2462402	1199133	2.05	24.5509
317	3880720	851291	29658	2540574	1238525	2.05	23.7442
318	3776373	828061	28028	2471728	1205369	2.05	26.7302
319	3777095	831857	43296	2471378	1206892	2.05	26.8511
320	3804796	832755	24470	2491346	1215595	2.05	25.0336
321	3810478	829914	34162	2494578	1217850	2.05	19.8619
322	3889508	851973	33316	2546198	1243261	2.05	24.8688
323	3951172	869726	42528	2581350	1263401	2.04	24.6779
324	3825887	830017	52258	2506974	1222089	2.05	26.5642
325	3791740	828765	22692	2482155	1210792	2.05	26.6811

326	3871126	839980	36202	2533790	1238449	2.05	28.2906
327	3877321	845148	33932	2539911	1238635	2.05	26.6033
328	3917554	859758	34924	2561194	1251197	2.05	23.236
329	4539250	964128	98481	2977045	1450048	2.05	17.7966
330	3772788	827216	21436	2470123	1203205	2.05	29.3825
331	3778313	835724	42803	2468950	1206714	2.05	26.6001
332	3820239	839501	33355	2499744	1220530	2.05	24.9607
333	3774817	828750	44552	2468516	1206588	2.05	25.5758
334	3817375	830836	25171	2501843	1218662	2.05	26.6742
335	3766956	824588	44099	2464803	1204871	2.05	26.4963
336	3808784	836459	22642	2491766	1215976	2.05	26.5824
337	3796601	830401	47700	2484603	1213108	2.05	26.6866
338	3763407	816654	45463	2466378	1203465	2.05	22.5072
339	3973820	867752	43722	2600541	1269174	2.05	26.1058
340	3771201	824443	23680	2470923	1204354	2.05	23.1012
341	3765593	825767	45514	2463153	1204045	2.05	30.3183
342	3775969	821875	46641	2471485	1208615	2.04	29.1087
343	3737684	810718	21878	2449405	1193453	2.05	30.2672
344	3775689	820445	22369	2474465	1204773	2.05	26.4692
345	3848341	834200	38408	2521832	1229427	2.05	26.5435
346	3763350	824275	23338	2463964	1200947	2.05	26.5744
347	3786297	833963	22887	2477413	1208335	2.05	26.9841
348	3801817	834797	27493	2487322	1214187	2.05	30.6139
349	3809180	839441	45794	2489980	1216342	2.05	29.4696
350	3776605	830366	21773	2471112	1204926	2.05	30.2723
351	3808514	832283	25052	2492893	1215897	2.05	27.5913
352	3795489	823135	47408	2483489	1213613	2.05	30.0055
353	4308507	912653	59098	2825162	1375250	2.05	30.0014
354	3997476	868529	33816	2617517	1276291	2.05	26.6965
355	3813507	822765	25492	2497793	1217757	2.05	30.2343
356	3706403	788660	42954	2432961	1186534	2.05	14.8371
357	3780892	818995	22722	2478182	1206155	2.05	30.1945
358	3949747	867354	35670	2583258	1260491	2.05	26.964
359	3866111	831769	35367	2533764	1236649	2.05	30.5716
360	3795162	825892	44811	2485001	1212763	2.05	30.4312
361	3792379	817971	23031	2487101	1210688	2.05	30.5082
362	3967091	855222	50065	2599062	1268690	2.05	26.4462
363	3767007	829007	23047	2464486	1201964	2.05	29.9974
364	3837442	836638	29748	2512980	1225418	2.05	26.0769
365	3859873	849472	30851	2521744	1233657	2.04	30.3182
366	3765853	821386	21441	2465482	1201311	2.05	29.9304
367	3766383	824819	49700	2465758	1203251	2.05	26.7006
368	3772105	894296	35235	2471369	1203388	2.05	25.1928
369	3868841	844902	32885	2532861	1235255	2.05	30.0905
370	3757865	816488	22473	2462684	1198994	2.05	25.885
371	3790870	829980	22901	2483021	1210867	2.05	26.7918
372	3807915	832098	23120	2493171	1214935	2.05	30.0799

373	3822017	839811	26282	2502668	1218571	2.05	26.6119
374	3783956	832932	22190	2477275	1207136	2.05	26.6443
375	3777203	832151	22990	2470924	1205051	2.05	30.6664
376	3853414	846499	34284	2519122	1229798	2.05	30.235
377	3771029	828869	43068	2465378	1206335	2.04	28.5855
378	3794211	829856	22316	2485903	1211464	2.05	25.3206
379	3804417	837286	49666	2490220	1215136	2.05	24.9006
380	3795251	834301	44425	2482644	1212756	2.05	26.8029
381	3787284	823824	26091	2482093	1208065	2.05	26.4029
382	3757136	832827	856	2454884	1198488	2.05	30.7578
383	3931644	863260	38406	2572694	1255006	2.05	26.669
384	3806240	839274	21653	2488095	1214394	2.05	38.4899
385	3812733	832168	468	2496271	1217780	2.05	26.538
386	3756920	827946	23752	2460124	1197426	2.05	26.7554
387	4027352	874358	710	2637695	1286393	2.05	26.6469
388	3774030	833941	23566	2467985	1203719	2.05	25.7134
389	3811329	840709	48724	2489975	1218738	2.04	26.7951
390	3791514	837951	28791	2480775	1208666	2.05	28.7512
391	3778724	819249	24121	2474413	1206872	2.05	28.9385
392	3907217	858969	57157	2557126	1245795	2.05	26.7516
393	3798211	836498	22037	2485616	1211635	2.05	26.4827
394	3841745	842297	35993	2515237	1225660	2.05	26.8033
395	4303434	929466	62139	2817993	1374438	2.05	26.6245
396	3819239	839067	48595	2497829	1220167	2.05	26.9195
397	3790194	836914	22070	2479141	1209349	2.05	26.6164
398	3786392	833826	22968	2478567	1207228	2.05	26.7174
399	3806048	827137	44464	2492011	1217324	2.05	26.2637
400	3818346	820139	26140	2503997	1217727	2.06	30.0048
401	4023514	875116	39579	2634198	1285101	2.05	27.1446
402	3745924	810548	22892	2454845	1195813	2.05	26.3785
403	3903160	828529	33253	2559770	1247232	2.05	29.7434
404	3831250	819883	29164	2512356	1222346	2.06	23.5465
405	3757723	813570	21550	2459553	1199026	2.05	29.4152
406	3924127	858471	38102	2566864	1252579	2.05	26.287
407	3743227	820448	22491	2448659	1196241	2.05	26.5391
408	4560822	970838	97579	2985661	1457011	2.05	28.5093
409	3766284	825479	22223	2464451	1201307	2.05	34.1081
410	3779257	831559	36821	2470422	1208719	2.04	32.9988
411	4793862	987178	85920	3148452	1532603	2.05	26.5337
412	3839136	831408	35044	2516225	1226030	2.05	25.2736
413	3775492	828589	44267	2468601	1206375	2.05	26.049
414	3822167	830072	47026	2504807	1219858	2.05	26.6438
415	3807280	831440	50663	2493676	1215774	2.05	26.6705
416	3821255	843895	21896	2499263	1219975	2.05	26.5521
417	3812699	832622	22858	2496953	1217758	2.05	26.5843
418	3846880	825155	31966	2519710	1227896	2.05	32.0621
419	3890016	844007	35722	2545626	1243289	2.05	28.4298

420	3856714	828241	33462	2527116	1233111	2.05	26.3756
421	3783953	814177	75157	2490910	1197733	2.08	20.5522
422	3828480	843248	30416	2508142	1217301	2.06	28.9824
423	3790554	831714	22601	2480163	1209635	2.05	28.3414
424	3816304	836441	23960	2496732	1217488	2.05	30.2542
425	4273287	917155	124241	2798850	1365659	2.05	22.8351
426	3793876	833261	25457	2482683	1210847	2.05	30.2615
427	3800249	826070	25606	2489507	1214586	2.05	24.5573
428	4579739	972439	101472	3000434	1463559	2.05	30.0962
429	3814557	832340	55092	2499256	1215468	2.06	30.3566
430	3904577	853435	51744	2556028	1245964	2.05	30.0359
431	3809711	835191	45265	2491140	1218138	2.05	30.1371
432	4162130	875543	348500	2736147	1335810	2.05	19.8626
433	3829435	845238	31555	2502440	1222434	2.05	30.7113
434	3797670	837880	21917	2483536	1211559	2.05	29.9922
435	3937946	861031	38127	2576547	1258187	2.05	27.1446
436	3790789	821112	23362	2482985	1209172	2.05	29.9538
437	3770429	815225	22272	2469516	1203447	2.05	28.9018
438	3824185	829249	47102	2505574	1221078	2.05	26.4831
439	4681066	991962	147216	3066037	1496190	2.05	29.7799
440	3905558	852958	74986	2553472	1246558	2.05	32.3582
441	3875229	848288	32206	2534825	1236715	2.05	29.1129
442	3776460	827796	44157	2472394	1206062	2.05	26.4389
443	3778224	832582	22742	2471326	1204993	2.05	29.8302
444	3801279	834840	23837	2487231	1213770	2.05	30.0074
445	3810541	833169	24985	2493776	1214947	2.05	32.1435
446	3798396	820231	22281	2487419	1212786	2.05	29.8567
447	3830540	829019	900	2509964	1223127	2.05	24.1984
448	3758420	816916	24016	2462710	1200227	2.05	23.0356
449	3663632	788144	29169	2402285	1170814	2.05	24.7809
450	3786216	817036	22913	2479698	1208665	2.05	29.8369
451	3905209	858048	33354	2553146	1246715	2.05	34.7706
452	3801135	827920	44458	2488154	1216027	2.05	21.2904
453	3933597	949551	35128	2568240	1253937	2.05	30.3456
454	3818268	840290	47676	2497237	1219786	2.05	26.5561
455	3806039	833602	48780	2488174	1216415	2.05	31.951
456	3781214	808518	45973	2477347	1208012	2.05	31.7225
457	4335992	924123	61938	2841419	1383424	2.05	32.0423
458	3124593	512448	28373	2076064	1010470	2.05	7.16597
459	3819330	973553	21892	2489614	1215206	2.05	31.969
460	3834827	826518	33104	2513115	1225721	2.05	33.2707
461	3755720	797982	43600	2462689	1201600	2.05	28.6195
462	4315693	918753	62254	2830771	1377919	2.05	30.5084
463	3759026	812052	23359	2463909	1200424	2.05	28.9384
464	3919554	861960	82178	2562339	1252687	2.05	28.5728
465	3790795	819309	58700	2482729	1211606	2.05	34.8107
466	3844937	927966	25153	2507843	1227691	2.04	31.5577

467	3741714	823172	1084	2448700	1194071	2.05	25.2243
468	3666485	770127	43125	2406915	1167660	2.06	28.0688
469	3832212	840019	31852	2508362	1222583	2.05	25.5291
470	3784269	883613	24247	2473736	1206093	2.05	28.2187
471	4341220	937273	87489	2844516	1384810	2.05	25.887
472	3757752	798582	44409	2463085	1200788	2.05	25.957
473	3749034	807648	44160	2454523	1197747	2.05	28.9994
474	3757233	799859	30985	2463713	1199178	2.05	30.3811
475	3813771	834797	23901	2496467	1216635	2.05	31.6697
476	4390978	930534	65597	2879029	1403373	2.05	32.826
477	3760214	821381	22592	2463351	1199716	2.05	25.4338
478	3854322	840202	29184	2526075	1230054	2.05	20.3601
479	3878289	841506	31973	2538837	1240004	2.05	28.1083
480	3770057	801530	23261	2471657	1203273	2.05	32.3348
481	3726059	798356	35926	2442047	1192176	2.05	28.7194
482	3826202	912033	96862	2506323	1220538	2.05	21.4337
483	3804642	835713	23713	2489902	1213450	2.05	32.4729
			19531404			2.047971	28.00518075

including 11 duplicates

Supplementary Table 4 SKAT analysis (SKAT, SKATO, RareComm, RareCommoO)[1] of gene prioritized from Whole Genome Sequencing Analysis (Combined race) SKAT for Entire gene and sub-regions defined by Exons (E) and Introns (I) (start and end of genomic locations)

Run	Group	Name	Sub	Chr	Start	End	Group	Gene	Sub	SKAT	SKATO	RareComm	RareCommO	Vars
37	2	CSMD2	E1I1	1	33513997	33519464	2	CSMD2	E1I1	0.482563013	0.710436595	0.675749868	0.675749868	91
21	2	CSMD2	ALL	1	33513998	34165842	2	CSMD2	ALL	0.719767791	0.831095894	0.551673709	0.547775103	10,540
38	2	CSMD2	I1E2	1	33516570	33519677	2	CSMD2	I1E2	0.764102086	0.430233012	0.905516691	0.456618186	51
39	2	CSMD2	E2I2	1	33519464	33519811	2	CSMD2	E2I2	0.306406462	0.346993333	0.435301144	0.428790989	8
40	2	CSMD2	I2E3	1	33519677	33519950	2	CSMD2	I2E3	0.189537107	0.189537921	0.33683945	0.33683945	3
41	2	CSMD2	E3I3	1	33519811	33521462	2	CSMD2	E3I3	0.069909067	0.12650145	0.122197039	0.122197039	22
42	2	CSMD2	I3E4	1	33519950	33521550	2	CSMD2	I3E4	0.069909067	0.12650145	0.121843638	0.121843638	22
43	2	CSMD2	E4I4	1	33521462	33523306	2	CSMD2	E4I4	0.101849521	0.178539183	0.122655554	0.122655554	38
44	2	CSMD2	I4E5	1	33521550	33523419	2	CSMD2	I4E5	0.101849521	0.178539183	0.123115589	0.123115589	38
45	2	CSMD2	E5I5	1	33523306	33524881	2	CSMD2	E5I5	0.37610568	0.573405477	0.487424404	0.487424404	20
46	2	CSMD2	I5E6	1	33523419	33525043	2	CSMD2	I5E6	0.353813117	0.547123589	0.483095738	0.483095738	21
47	2	CSMD2	E6I6	1	33524881	33527195	2	CSMD2	E6I6	0.218059946	0.361601187	0.482746406	0.482746406	23
48	2	CSMD2	I6E7	1	33525043	33527258	2	CSMD2	I6E7	0.217519093	0.361246423	0.476412436	0.476412436	23
49	2	CSMD2	E7I7	1	33527195	33533049	2	CSMD2	E7I7	0.555491147	0.780914173	0.759109019	0.759109019	67
50	2	CSMD2	I7E8	1	33527258	33533229	2	CSMD2	I7E8	0.587178324	0.811535654	0.780862103	0.780862103	67
51	2	CSMD2	E8I8	1	33533049	33533795	2	CSMD2	E8I8	0.646430961	0.821474605	0.818650168	0.818650168	7
52	2	CSMD2	I8E9	1	33533229	33533907	2	CSMD2	I8E9	0.752656017	0.697234443	0.903782628	0.781944101	8
53	2	CSMD2	E9I9	1	33533795	33537021	2	CSMD2	E9I9	0.211597292	0.126141295	0.30879151	0.132831981	64
54	2	CSMD2	I9E10	1	33533907	33537095	2	CSMD2	I9E10	0.192666675	0.131360927	0.292671996	0.149204305	63
55	2	CSMD2	E10I10	1	33537021	33537435	2	CSMD2	E10I10	0.552976494	0.448724595	0.550634478	0.275397897	7
56	2	CSMD2	I10E11	1	33537095	33537609	2	CSMD2	I10E11	0.594876393	0.775536038	0.59271514	0.579629026	8
57	2	CSMD2	E11I11	1	33537435	33540524	2	CSMD2	E11I11	0.528302309	0.485752566	0.587191182	0.547795894	42
58	2	CSMD2	I11E12	1	33537609	33540698	2	CSMD2	I11E12	0.566820178	0.376305956	0.624952553	0.438614782	41
59	2	CSMD2	E12I12	1	33540524	33541129	2	CSMD2	E12I12	0.553976033	0.736005959	0.551292886	0.551292886	5
60	2	CSMD2	I12E13	1	33540698	33541309	2	CSMD2	I12E13	0.281330385	0.409411518	0.284825595	0.284825595	4
61	2	CSMD2	E13I13	1	33541129	33542719	2	CSMD2	E13I13	0.658856575	0.854123812	0.704245262	0.704245262	30
62	2	CSMD2	I13E14	1	33541309	33542896	2	CSMD2	I13E14	0.671125805	0.864746119	0.714506873	0.714506873	34
63	2	CSMD2	E14I14	1	33542719	33546036	2	CSMD2	E14I14	0.532604463	0.451780829	0.733521403	0.446091937	56
64	2	CSMD2	I14E15	1	33542896	33546219	2	CSMD2	I14E15	0.682492857	0.528885218	0.892427998	0.480565112	56
65	2	CSMD2	E15I15	1	33546036	33550176	2	CSMD2	E15I15	0.602005802	0.245986325	0.706430923	0.235044285	62
66	2	CSMD2	I15E16	1	33546219	33550350	2	CSMD2	I15E16	0.478902341	0.198969478	0.603328724	0.196382496	61
67	2	CSMD2	E16I16	1	33550176	33567592	2	CSMD2	E16I16	0.540557121	0.761119728	0.655382612	0.655382612	238
68	2	CSMD2	I16E17	1	33550350	33567766	2	CSMD2	I16E17	0.539333277	0.759736839	0.653572931	0.653572931	236
69	2	CSMD2	E17I17	1	33567592	33569373	2	CSMD2	E17I17	0.457436947	0.457254704	0.35179465	0.334749845	28
70	2	CSMD2	I17E18	1	33567766	33569547	2	CSMD2	I17E18	0.451288061	0.531683014	0.329051559	0.324586204	30
71	2	CSMD2	E18I18	1	33569373	33571531	2	CSMD2	E18I18	0.367095829	0.509924832	0.380000537	0.881222341	34
72	2	CSMD2	I18E19	1	33569547	33571726	2	CSMD2	I18E19	0.323072177	0.512041402	0.362962888	0.362962888	33
73	2	CSMD2	E19I19	1	33571531	33572505	2	CSMD2	E19I19	0.008129557	0.014551304	0.003219061	0.003219061	14
74	2	CSMD2	I19E20	1	33571726	33572691	2	CSMD2	I19E20	0.008129557	0.014551304	0.004075426	0.004075426	13
75	2	CSMD2	E20I20	1	33572505	33577295	2	CSMD2	E20I20	0.456452523	0.085835123	0.604531447	0.167888064	115
76	2	CSMD2	I20E21	1	33572691	33577484	2	CSMD2	I20E21	0.456452523	0.085835122	0.584107906	0.160055098	115
77	2	CSMD2	E21I21	1	33577295	33580752	2	CSMD2	E21I21	0.256341756	0.412456468	0.356520875	0.394929807	60
78	2	CSMD2	I21E22	1	33577484	33580899	2	CSMD2	I21E22	0.256341756	0.412456468	0.337661326	0.37793698	59
79	2	CSMD2	E22I22	1	33580752	33583641	2	CSMD2	E22I22	0.835838086	0.743092608	0.739480128	0.746781037	48
80	2	CSMD2	I22E23	1	33580899	33583830	2	CSMD2	I22E23	0.835838086	0.743092608	0.734389366	0.704940101	49
81	2	CSMD2	E23I23	1	33583641	33586503	2	CSMD2	E23I23	0.422236851	0.625720691	0.609844627	0.609844627	55
82	2	CSMD2	I23E24	1	33583830	33586617	2	CSMD2	I23E24	0.432595077	0.638173955	0.62039416	0.62039416	56
83	2	CSMD2	E24I24	1	33586503	33587087	2	CSMD2	E24I24	0.264400559	0.402161519	0.411829174	0.411829174	12
84	2	CSMD2	I24E25	1	33586617	33587168	2	CSMD2	I24E25	0.319388345	0.37326044	0.487187167	0.721287066	13
85	2	CSMD2	E25I25	1	33587087	33600864	2	CSMD2	E25I25	0.476592245	0.687505289	0.590987124	0.503390479	226
86	2	CSMD2	I25E26	1	33587168	33601010	2	CSMD2	I25E26	0.47110428	0.701484755	0.583396485	0.583396485	228
87	2	CSMD2	E26I26	1	33600864	33602368	2	CSMD2	E26I26	0.921937237	0.9102387	1	0.918791802	29
88	2	CSMD2	I26E27	1	33601010	33602546	2	CSMD2	I26E27	0.942819475	0.882723547	1	0.984554419	26
89	2	CSMD2	E27I27	1	33602368	33605281	2	CSMD2	E27I27	0.966852106	0.855952179	1	0.805082361	37
90	2	CSMD2	I27E28	1	33602546	33605470	2	CSMD2	I27E28	0.964376745	0.785654754	1	0.768023991	36
91	2	CSMD2	E28I28	1	33605281	33605862	2	CSMD2	E28I28	0.714483349	0.433032793	0.922722049	0.52884767	4
92	2	CSMD2	I28E29	1	33605470	33605988	2	CSMD2	I28E29	0.445862507	0.293910166	0.607763995	0.379904796	10
93	2	CSMD2	E29I29	1	33605862	33611040	2	CSMD2	E29I29	0.936179131	1	1	1	87
94	2	CSMD2	I29E30	1	33605988	33611250	2	CSMD2	I29E30	0.944305563	1	1	1	85
95	2	CSMD2	E30I30	1	33611040	33614503	2	CSMD2	E30I30	0.414413842	0.61851807	0.629540366	0.629540366	42
96	2	CSMD2	I30E31	1	33611250	33614620	2	CSMD2	I30E31	0.416956846	0.623726668	0.634852619	0.634852619	40
97	2	CSMD2	E31I31	1	33614503	33616905	2	CSMD2	E31I31	0.288499713	0.457507985	0.395859293	0.395859293	43
98	2	CSMD2	I31E32	1	33614620	33616975	2	CSMD2	I31E32	0.274764408	0.43786043	0.384609977	0.384609977	40
99	2	CSMD2	E32I32	1	33616905	33617498	2	CSMD2	E32I32	0.725102162	0.878700313	0.76320443	0.76320443	5
100	2	CSMD2	I32E33	1	33616975	33617617	2	CSMD2	I32E33	0.73151325	0.677764271	0.849469155	0.681021211	12
101	2	CSMD2	E33I33	1	33617498	33622166	2	CSMD2	E33I33	0.965023711	0.916935564	1	0.952396467	71
102	2	CSMD2	I33E34	1	33617617	33622271	2	CSMD2	I33E34	0.960374963	0.640117576	1	0.736121693	64

103	2	CSMD2	E34I34	1	33622166	33623369	2	CSMD2	E34I34	0.311981251	0.489873822	0.278318722	0.278318722	20
104	2	CSMD2	I34E35	1	33622271	33623466	2	CSMD2	I34E35	0.311981251	0.489873822	0.27546922	0.27546922	20
105	2	CSMD2	E35I35	1	33623369	33624518	2	CSMD2	E35I35	0.665961406	0.853537915	0.56023715	0.56023715	10
106	2	CSMD2	I35E36	1	33623466	33624643	2	CSMD2	I35E36	0.769271491	0.922858284	0.640347339	0.640347339	11
107	2	CSMD2	E36I36	1	33624518	33625050	2	CSMD2	E36I36	0.371982213	0.532238416	0.332806557	0.332806557	6
108	2	CSMD2	I36E37	1	33624643	33625254	2	CSMD2	I36E37	0.57403692	0.779816168	0.735081416	0.735081416	9
109	2	CSMD2	E37I37	1	33625050	33626485	2	CSMD2	E37I37	0.539800675	0.457277267	0.714050419	0.337109417	18
110	2	CSMD2	I37E38	1	33625254	33626581	2	CSMD2	I37E38	0.404096164	0.313408785	0.600517446	0.253834968	15
111	2	CSMD2	E38I38	1	33626485	33633421	2	CSMD2	E38I38	0.636989938	0.242553175	0.712382938	0.321100766	80
112	2	CSMD2	I38E39	1	33626581	33633535	2	CSMD2	I38E39	0.547779891	0.183349328	0.622829601	0.245440379	80
113	2	CSMD2	E39I39	1	33633421	33635213	2	CSMD2	E39I39	0.296588588	0.377330353	0.288375418	0.29924971	20
114	2	CSMD2	I39E40	1	33633535	33635330	2	CSMD2	I39E40	0.480434789	0.69432631	0.433026187	0.433026187	21
115	2	CSMD2	E40I40	1	33635213	33636359	2	CSMD2	E40I40	0.100337093	0.163875966	0.147608675	0.147608675	11
116	2	CSMD2	I40E41	1	33635330	33636554	2	CSMD2	I40E41	0.127507538	0.217291022	0.174112925	0.174112925	17
117	2	CSMD2	E41I41	1	33636359	33646647	2	CSMD2	E41I41	0.477098556	0.274210543	0.66963935	0.345823951	138
118	2	CSMD2	I41E42	1	33636554	33646835	2	CSMD2	I41E42	0.499999252	0.284212817	0.684143738	0.339872675	133
119	2	CSMD2	E42I42	1	33646647	33652322	2	CSMD2	E42I42	0.924941927	1	1	1	78
120	2	CSMD2	I42E43	1	33646835	33652461	2	CSMD2	I42E43	0.910843195	1	0.982332222	0.932990086	76
121	2	CSMD2	E43I43	1	33652322	33657945	2	CSMD2	E43I43	0.665916878	0.865231706	0.843164772	0.843164772	66
122	2	CSMD2	I43E44	1	33652461	33658137	2	CSMD2	I43E44	0.683070029	0.877966041	0.857161106	0.857161106	67
123	2	CSMD2	E44I44	1	33657945	33662889	2	CSMD2	E44I44	0.842457969	0.529567738	0.865519449	0.33568188	47
124	2	CSMD2	I44E45	1	33658137	33663092	2	CSMD2	I44E45	0.82007393	0.349337424	0.860850124	0.218765854	46
125	2	CSMD2	E45I45	1	33662889	33692929	2	CSMD2	E45I45	0.948620154	0.633882025	0.992088363	0.560043954	424
126	2	CSMD2	I45E46	1	33663092	33693056	2	CSMD2	I45E46	0.950588729	0.662642951	0.999636579	0.589925352	424
127	2	CSMD2	E46I46	1	33692929	33698752	2	CSMD2	E46I46	0.944808204	0.766131675	1	0.754302076	74
128	2	CSMD2	I46E47	1	33693056	33698944	2	CSMD2	I46E47	0.945490045	0.808137913	0.990244641	0.817145066	76
129	2	CSMD2	E47I47	1	33698752	33700516	2	CSMD2	E47I47	0.609508899	0.339994853	0.531043885	0.400221263	23
130	2	CSMD2	I47E48	1	33698944	33700673	2	CSMD2	I47E48	0.580062077	0.558874792	0.509863345	0.635628155	22
131	2	CSMD2	E48I48	1	33700516	33709088	2	CSMD2	E48I48	0.917827185	0.611128332	0.994317669	0.730567407	128
132	2	CSMD2	I48E49	1	33700673	33709258	2	CSMD2	I48E49	0.927301231	0.671113349	1	0.766845538	128
133	2	CSMD2	E49I49	1	33709088	33714586	2	CSMD2	E49I49	0.653859647	0.814085762	0.867177286	0.867177286	86
134	2	CSMD2	I49E50	1	33709258	33714775	2	CSMD2	I49E50	0.640413944	0.811207892	0.860565935	0.860565935	85
135	2	CSMD2	E50I50	1	33714586	33716285	2	CSMD2	E50I50	0.926803096	1	0.742592329	0.542874103	27
136	2	CSMD2	I50E51	1	33714775	33716501	2	CSMD2	I50E51	0.927376661	1	0.747239095	0.540339206	27
137	2	CSMD2	E51I51	1	33716285	33724196	2	CSMD2	E51I51	0.566692853	0.788855785	0.498222231	0.498222231	96
138	2	CSMD2	I51E52	1	33716501	33724313	2	CSMD2	I51E52	0.626799464	0.841644998	0.536726572	0.536726572	101
139	2	CSMD2	E52I52	1	33724196	33724515	2	CSMD2	E52I52	0.534354745	0.157919791	0.075038523	0.092727136	11
140	2	CSMD2	I52E53	1	33724313	33724704								6
141	2	CSMD2	E53I53	1	33724515	33725348	2	CSMD2	E53I53	0.376856973	0.282595864	0.376649501	0.270837731	12
142	2	CSMD2	I53E54	1	33724704	33725536	2	CSMD2	I53E54	0.377549653	0.121073738	0.431943194	0.135624348	15
143	2	CSMD2	E54I54	1	33725348	33726546	2	CSMD2	E54I54	0.506618204	0.716968082	0.602530808	0.602530808	20
144	2	CSMD2	I54E55	1	33725536	33726685	2	CSMD2	I54E55	0.548450092	0.760486229	0.639439045	0.639439045	17
145	2	CSMD2	E55I55	1	33726546	33739139	2	CSMD2	E55I55	0.153351186	0.268199322	0.251273778	0.251273778	206
146	2	CSMD2	I55E56	1	33726685	33739334	2	CSMD2	I55E56	0.149756159	0.262510019	0.261896071	0.261896071	206
147	2	CSMD2	E56I56	1	33739139	33743279	2	CSMD2	E56I56	0.728461258	0.124777321	0.727404136	0.086351059	80
148	2	CSMD2	I56E57	1	33739334	33743606	2	CSMD2	I56E57	0.8148852	0.196602879	0.830986127	0.162028943	81
149	2	CSMD2	E57I57	1	33743279	33772568	2	CSMD2	E57I57	0.7611047	0.838064852	0.893707159	0.714453065	515
150	2	CSMD2	I57E58	1	33743606	33772751	2	CSMD2	I57E58	0.745552508	0.83721543	0.924398797	0.713559381	514
151	2	CSMD2	E58I58	1	33772568	33788599	2	CSMD2	E58I58	0.100470375	0.163395283	0.194169299	0.194169299	254
152	2	CSMD2	I58E59	1	33772751	33788712	2	CSMD2	I58E59	0.105323885	0.171238542	0.196836901	0.196836901	254
153	2	CSMD2	E59I59	1	33788599	33792422	2	CSMD2	E59I59	0.043735321	0.063639179	0.030791067	0.030791067	58
154	2	CSMD2	I59E60	1	33788712	33792526	2	CSMD2	I59E60	0.040873975	0.059113063	0.029371459	0.029371459	58
155	2	CSMD2	E60I60	1	33792422	33810742	2	CSMD2	E60I60	0.298311982	0.466680451	0.283429644	0.283429644	312
156	2	CSMD2	I60E61	1	33792526	33810864	2	CSMD2	I60E61	0.297562844	0.465788546	0.282547422	0.282547422	315
157	2	CSMD2	E61I61	1	33810742	33819712	2	CSMD2	E61I61	0.90918373	0.805575468	0.349353717	0.604586064	172
158	2	CSMD2	I61E62	1	33810864	33819837	2	CSMD2	I61E62	0.910152281	0.800792334	0.399458686	0.623522486	171
159	2	CSMD2	E62I62	1	33819712	33820468	2	CSMD2	E62I62	0.634716011	0.540367221	0.802589516	0.670724093	12
160	2	CSMD2	I62E63	1	33819837	33820556	2	CSMD2	I62E63	0.60888035	0.369367801	0.717110189	0.490203445	9
161	2	CSMD2	E63I63	1	33820468	33825696	2	CSMD2	E63I63	0.201102729	0.338871637	0.326969346	0.326969346	109
162	2	CSMD2	I63E64	1	33820556	33825774	2	CSMD2	I63E64	0.201102729	0.338871637	0.332996704	0.332996704	110
163	2	CSMD2	E64I64	1	33825696	33846883	2	CSMD2	E64I64	0.805164428	1	0.279118932	0.279118932	426
164	2	CSMD2	I64E65	1	33825774	33846996	2	CSMD2	I64E65	0.806635266	1	0.282628706	0.282628706	426
165	2	CSMD2	E65I65	1	33846883	33918093	2	CSMD2	E65I65	0.19237192	0.317450359	0.070829258	0.070829258	1,119
166	2	CSMD2	I65E66	1	33846996	33918301	2	CSMD2	I65E66	0.192272012	0.317316961	0.070612812	0.070612812	1,119
167	2	CSMD2	E66I66	1	33918093	33935759	2	CSMD2	E66I66	0.251309832	0.401212676	0.334593386	0.334593386	237
168	2	CSMD2	I66E67	1	33918301	33935954	2	CSMD2	I66E67	0.275956387	0.433601913	0.348368994	0.348368994	239
169	2	CSMD2	E67I67	1	33935759	34032593	2	CSMD2	E67I67	0.710177973	0.851319534	0.533784019	0.379306347	1,694
170	2	CSMD2	I67E68	1	33935954	34032706	2	CSMD2	I67E68	0.705438965	0.854858304	0.51973764	0.372521757	1,692
171	2	CSMD2	E68I68	1	34032593	34088976	2	CSMD2	E68I68	0.430021468	0.638233989	0.393860572	0.393860572	919
172	2	CSMD2	I68E69	1	34032706	34089193	2	CSMD2	I68E69	0.434543775	0.642510107	0.39481	0.39481	919
173	2	CSMD2	E69I69	1	34088976	34165746	2	CSMD2	E69I69	0.845684389	0.855792016	0.853559177	0.764414122	1,340
174	2	CSMD2	I69E70	1	34089193	34165842	2	CSMD2	I69E70	0.835917933	0.86881159	0.841818204	0.775582428	1,341

175	2	MAN1A2	E1I1	1	117367448	117402185	2	MAN1A2	E1I1	0.091934904	0.031470637	0.065330138	0.038096368	527
22	2	MAN1A2	ALL	1	117367449	117528872	2	MAN1A2	ALL	0.10114757	0.030342501	0.053443415	0.020697287	2,383
176	2	MAN1A2	I1E2	1	117368485	117402441	2	MAN1A2	I1E2	0.095093805	0.038693651	0.064700971	0.044855713	526
177	2	MAN1A2	E2I2	1	117402185	117405548	2	MAN1A2	E2I2	0.418475543	0.23059296	0.048781807	0.027145883	60
178	2	MAN1A2	I2E3	1	117402441	117405645	2	MAN1A2	I2E3	0.444533707	0.121614746	0.05582377	0.027128507	53
179	2	MAN1A2	E3I3	1	117405548	117414712	2	MAN1A2	E3I3	0.105238296	0.030707998	0.060173254	0.046540901	138
180	2	MAN1A2	I3E4	1	117405645	117414831	2	MAN1A2	I3E4	0.096014588	0.027143211	0.05749665	0.061603785	139
181	2	MAN1A2	E4I4	1	117414712	117420568	2	MAN1A2	E4I4	0.194192829	0.32844244	0.066394428	0.066394428	80
182	2	MAN1A2	I4E5	1	117414831	117420649	2	MAN1A2	I4E5	0.228424774	0.379090865	0.077601392	0.077601392	79
183	2	MAN1A2	E5I5	1	117420568	117442230	2	MAN1A2	E5I5	0.300380976	0.193676609	0.133600663	0.079176541	264
184	2	MAN1A2	I5E6	1	117420649	117442325	2	MAN1A2	I5E6	0.300380976	0.193676609	0.133451166	0.078921246	264
185	2	MAN1A2	E6I6	1	117442230	117460488	2	MAN1A2	E6I6	0.121296994	0.051985325	0.050273866	0.034987619	281
186	2	MAN1A2	I6E7	1	117442325	117460612	2	MAN1A2	I6E7	0.121296994	0.051985325	0.049138086	0.035481139	281
187	2	MAN1A2	E7I7	1	117460488	117466333	2	MAN1A2	E7I7	0.165529972	0.203017918	0.031653836	0.040181657	83
188	2	MAN1A2	I7E8	1	117460612	117466427	2	MAN1A2	I7E8	0.161228247	0.167302408	0.03136132	0.039808638	86
189	2	MAN1A2	E8I8	1	117466333	117493146	2	MAN1A2	E8I8	0.237708848	0.18043708	0.116946937	0.072569159	409
190	2	MAN1A2	I8E9	1	117466427	117493262	2	MAN1A2	I8E9	0.239276131	0.188996012	0.118695736	0.073358034	406
191	2	MAN1A2	E9I9	1	117493146	117496762	2	MAN1A2	E9I9	0.332256694	0.226263187	0.456883501	0.031286281	43
192	2	MAN1A2	I9E10	1	117493262	117496982	2	MAN1A2	I9E10	0.337727972	0.277481472	0.468670944	0.10423232	45
193	2	MAN1A2	E10I10	1	117496762	117499381	2	MAN1A2	E10I10	0.183001278	0.177877356	0.062343606	0.049027097	42
194	2	MAN1A2	I10E11	1	117496982	117499554	2	MAN1A2	I10E11	0.211090163	0.321519471	0.067259957	0.053422976	48
195	2	MAN1A2	E11I11	1	117499381	117502854	2	MAN1A2	E11I11	0.200165282	0.333269607	0.024109392	0.024109392	55
196	2	MAN1A2	I11E12	1	117499554	117502970	2	MAN1A2	I11E12	0.160237122	0.270391019	0.02138294	0.02138294	47
197	2	MAN1A2	E12I12	1	117502854	117522824	2	MAN1A2	E12I12	0.07280996	0.013314733	0.030840995	0.011488214	300
198	2	MAN1A2	I12E13	1	117502970	117528872	2	MAN1A2	I12E13	0.042054781	0.052232485	0.021974162	0.021499824	401
199	2	PPP2R5A	E1I1	1	212285409	212329134	2	PPP2R5A	E1I1	0.829999693	1	0.072050498	0.072050498	654
23	2	PPP2R5A	ALL	1	212285410	212361853	2	PPP2R5A	ALL	0.906137757	0.721604409	0.057100491	0.070046701	1,112
200	2	PPP2R5A	I1E2	1	212286291	212329331	2	PPP2R5A	I1E2	0.806072733	1	0.069676877	0.069676877	639
201	2	PPP2R5A	E2I2	1	212329134	212333496	2	PPP2R5A	E2I2	0.906239305	0.126159186	0.014778943	0.002365249	64
202	2	PPP2R5A	I2E3	1	212329331	212333598	2	PPP2R5A	I2E3	0.906239305	0.126159186	0.014326787	0.002533346	64
203	2	PPP2R5A	E3I3	1	212333496	212342187	2	PPP2R5A	E3I3	0.986414863	1	0.165556804	0.884617323	103
204	2	PPP2R5A	I3E4	1	212333598	212342280	2	PPP2R5A	I3E4	0.986985271	1	0.128217998	1	105
205	2	PPP2R5A	E4I4	1	212342187	212345802	2	PPP2R5A	E4I4	0.962945298	0.481916956	0.220760217	0.562458986	61
206	2	PPP2R5A	I4E5	1	212342280	212345933	2	PPP2R5A	I4E5	0.951715997	0.613068935	0.314433934	0.382370317	60
207	2	PPP2R5A	E5I5	1	212345802	212347346	2	PPP2R5A	E5I5	0.848696724	0.807002573	1	0.796772548	15
208	2	PPP2R5A	I5E6	1	212345933	212347406	2	PPP2R5A	I5E6	0.92555981	0.435075117	1	0.488136356	16
209	2	PPP2R5A	E6I6	1	212347346	212348388	2	PPP2R5A	E6I6	0.25062687	0.404347065	0.007013594	0.007013594	23
210	2	PPP2R5A	I6E7	1	212347406	212348497	2	PPP2R5A	I6E7	0.217869684	0.361750564	0.005649375	0.005649375	21
211	2	PPP2R5A	E7I7	1	212348388	212349188	2	PPP2R5A	E7I7	0.689156761	0.854088816	0.567020471	0.567020471	12
212	2	PPP2R5A	I7E8	1	212348497	212349242	2	PPP2R5A	I7E8	0.689156761	0.854088816	0.562793182	0.562793182	12
213	2	PPP2R5A	E8I8	1	212349188	212356625	2	PPP2R5A	E8I8	0.635982889	0.306286874	0.065680053	0.004961745	109
214	2	PPP2R5A	I8E9	1	212349242	212356676	2	PPP2R5A	I8E9	0.635982889	0.306286874	0.065794558	0.005219306	109
215	2	PPP2R5A	E9I9	1	212356625	212356949	2	PPP2R5A	E9I9	0.346810176	0.435703444	0.347604105	0.260708572	4
216	2	PPP2R5A	I9E10	1	212356676	212357069	2	PPP2R5A	I9E10	0.296626417	0.420746403	0.297309855	0.297309855	5
217	2	PPP2R5A	E10I10	1	212356949	212357156								1
218	2	PPP2R5A	I10E11	1	212357069	212357284								0
219	2	PPP2R5A	E11I11	1	212357156	212358685	2	PPP2R5A	E11I11	0.568886543	0.129910286	0.017322855	0.001816236	35
220	2	PPP2R5A	I11E12	1	212357284	212358787	2	PPP2R5A	I11E12	0.568886543	0.129910286	0.015227137	0.001298991	35
221	2	PPP2R5A	E12I12	1	212358685	212360637	2	PPP2R5A	E12I12	0.780295151	1	0.700378747	0.700378747	21
222	2	PPP2R5A	I12E13	1	212358787	212361853	2	PPP2R5A	I12E13	0.795090619	1	0.238087215	0.238087215	31
237	2	LINC01317	E1I1	2	33706885	33855687	2	LINC01317	E1I1	0.229572253	0.354982397	0.086398725	0.086398725	3,701
24	2	LINC01317	ALL	2	33706886	34297746	2	LINC01317	ALL	0.613848336	0.813885637	0.062152001	0.062152001	14,118
238	2	LINC01317	I1E2	2	33706953	33855753	2	LINC01317	I1E2	0.22989305	0.355497095	0.086466941	0.086466941	3,702
239	2	LINC01317	E2I2	2	33855687	33926839	2	LINC01317	E2I2	0.53197349	0.729680495	0.008009171	0.008009171	1,812
240	2	LINC01317	I2E3	2	33855753	33926968	2	LINC01317	I2E3	0.532074318	0.72970763	0.008059437	0.008059437	1,812
241	2	LINC01317	E3I3	2	33926839	33944427	2	LINC01317	E3I3	0.183380358	0.281412143	0.02709306	0.02709306	366
242	2	LINC01317	I3E4	2	33926968	33944553	2	LINC01317	I3E4	0.182919352	0.280690872	0.026398448	0.026398448	366
243	2	LINC01317	E4I4	2	33944427	34286642	2	LINC01317	E4I4	0.796026391	1	0.486938694	0.486938694	7,997
244	2	LINC01317	I4E5	2	33944553	34286746	2	LINC01317	I4E5	0.79754967	1	0.481351633	0.481351633	7,994
245	2	LINC01317	E5I5	2	34286642	34289260	2	LINC01317	E5I5	0.911797585	1	1	0.994602852	51
246	2	LINC01317	I5E6	2	34286746	34289266	2	LINC01317	I5E6	0.917738326	1	1	1	52
247	2	LINC01317	E6I6	2	34289260	34297473	2	LINC01317	E6I6	0.940543002	0.893681575	0.233300583	0.334922065	181
248	2	LINC01317	I6E7	2	34289266	34297746	2	LINC01317	I6E7	0.937664814	1	0.249264581	0.315280747	192
291	2	LINC02211	E1I1	5	25190952	25297702	2	LINC02211	E1I1	0.712111781	0.36531859	0.576779802	0.500178387	1,757
25	2	LINC02211	ALL	5	25190953	25302280	2	LINC02211	ALL	0.734733599	0.366814836	0.600481095	0.517628471	1,838
27	2	LINC02211;CDH9	ALL	5	25190953	27038586	2	LINC02211;CDH9	ALL	0.815259959	0.231042767	0.841536277	0.087713623	35,613
292	2	LINC02211	I1E2	5	25191033	25297803	2	LINC02211	I1E2	0.722555573	0.360272865	0.578891178	0.49085121	1,759
293	2	LINC02211	E2I2	5	25297702	25298978	2	LINC02211	E2I2	0.435650248	0.641349804	0.564891862	0.564891862	23
294	2	LINC02211	I2E3	5	25297803	25299060	2	LINC02211	I2E3	0.573240366	0.781560093	0.71016032	0.71016032	23
295	2	LINC02211	E3I3	5	25298978	25302217	2	LINC02211	E3I3	0.918976028	0.564279011	0.874712462	0.677507557	52
296	2	LINC02211	I3E4	5	25299060	25302280	2	LINC02211	I3E4	0.901322472	0.644180563	0.820429255	0.73903405	55
297	2	CDH9	E1I1	5	26880596	26885613	2	CDH9	E1I1	0.44621924	0.148589303	0.485615921	0.077545774	87

26	2	CDH9	ALL	5	26880597	27038586	2	CDH9	ALL	0.715549138	0.74051048	0.792758426	0.683681578	2,883
298	2	CDH9	I1E2	5	26881623	26885865	2	CDH9	I1E2	0.462235846	0.123861183	0.47345806	0.051156591	87
299	2	CDH9	E2I2	5	26885613	26885965	2	CDH9	E2I2	0.392370605	0.559276729	0.395882316	0.395882316	9
300	2	CDH9	I2E3	5	26885865	26886083	2	CDH9	I2E3	1	1	1	1	3
301	2	CDH9	E3I3	5	26885965	26889835	2	CDH9	E3I3	0.380907046	0.588113918	0.391698852	0.391698852	63
302	2	CDH9	I3E4	5	26886083	26889957	2	CDH9	I3E4	0.39763436	0.608789525	0.397135224	0.397135224	64
303	2	CDH9	E4I4	5	26889835	26890427	2	CDH9	E4I4	0.52375341	0.72158732	0.587213488	0.587213488	6
304	2	CDH9	I4E5	5	26889957	26890564	2	CDH9	I4E5	0.428479557	0.472821005	0.504165337	0.332569081	7
305	2	CDH9	E5I5	5	26890427	26902475	2	CDH9	E5I5	0.657434934	0.85554806	0.307437638	0.307437638	206
306	2	CDH9	I5E6	5	26890564	26902729	2	CDH9	I5E6	0.635702442	0.839370378	0.293141738	0.293141738	206
307	2	CDH9	E6I6	5	26902475	26903636	2	CDH9	E6I6	0.282553753	0.447917944	0.0761331	0.212103476	18
308	2	CDH9	I6E7	5	26902729	26903824	2	CDH9	I6E7	0.471520148	0.670555315	0.111711505	0.111711505	25
309	2	CDH9	E7I7	5	26903636	26905958	2	CDH9	E7I7	0.862983979	0.656749237	0.934629713	0.633183499	32
310	2	CDH9	I7E8	5	26903824	26906126	2	CDH9	I7E8	0.865858094	0.680057386	0.939207225	0.630990755	27
311	2	CDH9	E8I8	5	26905958	26906718	2	CDH9	E8I8	0.53904801	0.710188302	0.400273562	0.731999612	15
312	2	CDH9	I8E9	5	26906126	26906838	2	CDH9	I8E9	0.456361573	0.633458912	0.395957426	0.395957426	12
313	2	CDH9	E9I9	5	26906718	26915629	2	CDH9	E9I9	0.298120405	0.485611339	0.199877971	0.199877971	146
314	2	CDH9	I9E10	5	26906838	26915924	2	CDH9	I9E10	0.303001007	0.492508775	0.198916608	0.198916608	147
315	2	CDH9	E10I10	5	26915629	26988105	2	CDH9	E10I10	0.750983293	0.72309295	0.906953119	0.564377982	1,473
316	2	CDH9	I10E11	5	26915924	26988382	2	CDH9	I10E11	0.750303205	0.727619191	0.89508718	0.55621148	1,473
317	2	CDH9	E11I11	5	26988105	27038462	2	CDH9	E11I11	0.600709825	0.817069751	0.579562697	0.579562697	826
318	2	CDH9	I11E12	5	26988382	27038586	2	CDH9	I11E12	0.600442633	0.816887067	0.590640354	0.590640354	826
405	2	HIVEP1	E1I1	6	12007692	12015525	2	HIVEP1	E1I1	0.668955483	0.826419834	0.610652872	0.752424614	171
28	2	HIVEP1	ALL	6	12007693	12212048	2	HIVEP1	ALL	0.134307132	0.230948026	0.054108982	0.054108982	3,593
30	2	HIVEP1;EDN1	ALL	6	12007693	12297194	2	HIVEP1;EDN1	ALL	0.19218684	0.321951584	0.01895504	0.01895504	5,038
406	2	HIVEP1	I1E2	6	12007989	12015668	2	HIVEP1	I1E2	0.576195348	0.746002591	0.542370353	0.685833993	167
407	2	HIVEP1	E2I2	6	12015525	12020247	2	HIVEP1	E2I2	0.085292333	0.142898434	0.021839329	0.021839329	121
408	2	HIVEP1	I2E3	6	12015668	12020418	2	HIVEP1	I2E3	0.084573002	0.141824045	0.020847706	0.020847706	120
409	2	HIVEP1	E3I3	6	12020247	12089183	2	HIVEP1	E3I3	0.304939392	0.48100225	0.159923284	0.159923284	1,232
410	2	HIVEP1	I3E4	6	12020418	12089237	2	HIVEP1	I3E4	0.304939392	0.48100225	0.159574864	0.159574864	1,232
411	2	HIVEP1	E4I4	6	12089183	12119889	2	HIVEP1	E4I4	0.157346152	0.255511958	0.042647024	0.042647024	488
412	2	HIVEP1	I4E5	6	12089237	12125870	2	HIVEP1	I4E5	0.120456882	0.197857723	0.031663655	0.031663655	578
413	2	HIVEP1	E5I5	6	12119889	12129758	2	HIVEP1	E5I5	0.138315971	0.231550681	0.032135407	0.032135407	164
414	2	HIVEP1	I5E6	6	12125870	12129892	2	HIVEP1	I5E6	0.348907055	0.52867566	0.094137361	0.094137361	75
415	2	HIVEP1	E6I6	6	12129758	12130766	2	HIVEP1	E6I6	0.186481438	0.296784127	0.090456859	0.090456859	16
416	2	HIVEP1	I6E7	6	12129892	12130942	2	HIVEP1	I6E7	0.181180846	0.290010399	0.087363499	0.087363499	18
417	2	HIVEP1	E7I7	6	12130766	12135790	2	HIVEP1	E7I7	0.215820902	0.365738933	0.171006979	0.171006979	70
418	2	HIVEP1	I7E8	6	12130942	12135892	2	HIVEP1	I7E8	0.217747574	0.368219089	0.175406708	0.175406708	67
419	2	HIVEP1	E8I8	6	12135790	12161438	2	HIVEP1	E8I8	0.191609939	0.324070628	0.248081097	0.248081097	438
420	2	HIVEP1	I8E9	6	12135892	12161929	2	HIVEP1	I8E9	0.192131824	0.324896582	0.258283504	0.258283504	443
421	2	HIVEP1	E9I9	6	12161438	12163282	2	HIVEP1	E9I9	0.081305315	0.016128048	0.057106281	0.013348951	20
422	2	HIVEP1	I9E10	6	12161929	12164999	2	HIVEP1	I9E10	0.046005636	0.002220631	0.031132262	0.001993223	41
29	2	EDN1	ALL	6	12230516	12297194	2	EDN1	ALL	0.407594993	0.608685545	0.039745527	0.039745527	1,049
423	2	EDN1	E1I1	6	12288401	12290628	2	EDN1	E1I1	0.121732813	0.187240835	0.09125094	0.09125094	32
424	2	EDN1	I1E2	6	12288495	12290693	2	EDN1	I1E2	0.118825039	0.181626251	0.089985497	0.089985497	31
425	2	EDN1	E2I2	6	12290628	12292343	2	EDN1	E2I2	0.438721671	0.648955104	0.549024171	0.549024171	27
426	2	EDN1	I2E3	6	12290693	12292509	2	EDN1	I2E3	0.515684158	0.736976616	0.626787896	0.626787896	31
427	2	EDN1	E3I3	6	12292343	12293940	2	EDN1	E3I3	0.508953863	0.727010836	0.423787824	0.423787824	26
428	2	EDN1	I3E4	6	12292509	12294096	2	EDN1	I3E4	0.462358195	0.671503768	0.460356676	0.460356676	24
429	2	EDN1	E4I4	6	12293940	12294260	2	EDN1	E4I4	0.75467631	0.484029467	1	0.429002838	4
430	2	EDN1	I4E5	6	12294096	12294404	2	EDN1	I4E5	0.642943567	0.767425112	0.7783107	0.632646491	5
431	2	EDN1	E5I5	6	12294260	12295961	2	EDN1	E5I5	0.2926598	0.451606131	0.17626496	0.17626496	25
432	2	EDN1	I5E6	6	12294404	12297194	2	EDN1	I5E6	0.612714659	0.81242819	0.470647863	0.470647863	38
433	2	HSP90AB1	E1I1	6	44246193	44248629	2	HSP90AB1	E1I1	0.416624092	0.481114965	0.178504148	0.569937354	78
31	2	HSP90AB1	ALL	6	44246194	44253883	2	HSP90AB1	ALL	0.35734941	0.252038438	0.09470644	0.19360093	161
434	2	HSP90AB1	I1E2	6	44246344	44248776	2	HSP90AB1	I1E2	0.352523612	0.55874679	0.156920351	0.156920351	78
435	2	HSP90AB1	E2I2	6	44248629	44249376	2	HSP90AB1	E2I2	0.204873207	0.329535329	0.211929148	0.211929148	8
436	2	HSP90AB1	I2E3	6	44248776	44249583	2	HSP90AB1	I2E3	0.214487965	0.321568961	0.214702238	0.214702238	6
437	2	HSP90AB1	E3I3	6	44249376	44249674								2
438	2	HSP90AB1	I3E4	6	44249583	44249834	2	HSP90AB1	I3E4	0.609345358	0.661773394	0.802081642	0.802081642	2
439	2	HSP90AB1	E4I4	6	44249674	44250020	2	HSP90AB1	E4I4	0.795662412	0.597389134	0.379492274	0.335996708	6
440	2	HSP90AB1	I4E5	6	44249834	44250154	2	HSP90AB1	I4E5	0.74277239	0.663023213	0.362132036	0.359327328	5
441	2	HSP90AB1	E5I5	6	44250020	44250290	2	HSP90AB1	E5I5	0.91071813	0.74106249	1	0.674264746	2
442	2	HSP90AB1	I5E6	6	44250154	44250599	2	HSP90AB1	I5E6	0.148414503	0.04072162	0.027802216	0.007579383	5
443	2	HSP90AB1	E6I6	6	44250290	44251047	2	HSP90AB1	E6I6	0.040642541	0.07007956	0.01024722	0.01024722	14
444	2	HSP90AB1	I6E7	6	44250599	44251213	2	HSP90AB1	I6E7	0.245962637	0.269913611	0.177986544	0.144216829	13
445	2	HSP90AB1	E7I7	6	44251047	44251417	2	HSP90AB1	E7I7	0.451389315	0.463812595	0.373220878	0.276087891	5
446	2	HSP90AB1	I7E8	6	44251213	44251608	2	HSP90AB1	I7E8	0.282313723	0.393250438	0.244016447	0.234426926	3
447	2	HSP90AB1	E8I8	6	44251417	44251736	2	HSP90AB1	E8I8	1	1	1	1	1
448	2	HSP90AB1	I8E9	6	44251608	44251884	2	HSP90AB1	I8E9	0.731090258	0.418158535	0.815002093	0.261737707	4
449	2	HSP90AB1	E9I9	6	44251736	44251998	2	HSP90AB1	E9I9	0.435499597	0.620816129	0.491353154	0.491353154	6
450	2	HSP90AB1	I9E10	6	44251884	44252267	2	HSP90AB1	I9E10	0.41077094	0.392497583	0.476875646	0.400278325	7

451	2	HSP90AB1	E10I10	6	44251998	44253044	2	HSP90AB1	E10I10	0.307995994	0.021791646	0.03971747	0.049951305	26
452	2	HSP90AB1	I10E11	6	44252267	44253378	2	HSP90AB1	I10E11	0.349573398	0.091409723	0.042771774	0.156541303	23
453	2	HSP90AB1	E11I11	6	44253044	44253488	2	HSP90AB1	E11I11	0.608677833	0.776608636	0.11811521	0.11811521	5
454	2	HSP90AB1	I11E12	6	44253378	44253883	2	HSP90AB1	I11E12	0.555617032	0.760677975	0.100735485	0.100735485	12
487	2	TTLL11	E1I1	9	121815673	121860336	2	TTLL11	E1I1	0.895601767	0.594760207	0.021330647	0.026568468	885
32	2	TTLL11	ALL	9	121815674	122093308	2	TTLL11	ALL	0.899143409	0.727173652	0.189951462	0.778176119	4,770
488	2	TTLL11	I1E2	9	121822879	121860443	2	TTLL11	I1E2	0.921874723	0.648663359	0.020616774	0.025149927	753
489	2	TTLL11	E2I2	9	121860336	121870496	2	TTLL11	E2I2	0.27743165	0.454352208	0.001290198	0.001290198	163
490	2	TTLL11	I2E3	9	121860443	121870748	2	TTLL11	I2E3	0.300195737	0.484209712	0.001030083	0.001030083	164
491	2	TTLL11	E3I3	9	121870496	121974008	2	TTLL11	E3I3	0.790306416	0.548799622	0.241134538	0.414807734	1,796
492	2	TTLL11	I3E4	9	121870748	121974124	2	TTLL11	I3E4	0.789782463	0.554576015	0.228690625	0.411804889	1,794
493	2	TTLL11	E4I4	9	121974008	121974883	2	TTLL11	E4I4	0.814288052	0.584816102	0.788954563	0.33277078	8
494	2	TTLL11	I4E5	9	121974124	121974979	2	TTLL11	I4E5	0.814288052	0.584816102	0.795161756	0.322066525	8
495	2	TTLL11	E5I5	9	121974883	121989194	2	TTLL11	E5I5	0.610176079	0.817228266	0.322790131	0.322790131	218
496	2	TTLL11	I5E6	9	121974979	121989770	2	TTLL11	I5E6	0.640812794	0.842162452	0.342727657	0.342727657	223
497	2	TTLL11	E6I6	9	121989194	122031722	2	TTLL11	E6I6	0.737143283	0.869544119	0.733475888	0.733475888	770
498	2	TTLL11	I6E7	9	121989770	122031856	2	TTLL11	I6E7	0.720504915	0.85834461	0.723866981	0.723866981	768
499	2	TTLL11	E7I7	9	122031722	122039271	2	TTLL11	E7I7	0.886935396	0.62511703	1	0.676875843	124
500	2	TTLL11	I7E8	9	122031856	122039368	2	TTLL11	I7E8	0.949268504	0.709569003	1	0.744929032	122
501	2	TTLL11	E8I8	9	122039271	122092686	2	TTLL11	E8I8	0.684125346	0.873387601	0.934801957	0.934801957	798
502	2	TTLL11	I8E9	9	122039368	122093308	2	TTLL11	I8E9	0.685106054	0.874162837	0.930734354	0.930734354	805
509	2	SFMBT2	E1I1	10	7158623	7170927	2	SFMBT2	E1I1	0.76876827	0.108958529	0.819024324	0.12896327	202
33	2	SFMBT2	ALL	10	7158624	7411490	2	SFMBT2	ALL	0.104665066	0.030691073	0.026645199	0.011137512	4,818
510	2	SFMBT2	I1E2	10	7163910	7171056	2	SFMBT2	I1E2	0.745163671	0.110320542	0.792597096	0.106796182	115
511	2	SFMBT2	E2I2	10	7170927	7171894	2	SFMBT2	E2I2	0.47467379	0.069841467	0.4519706	0.03925855	11
512	2	SFMBT2	I2E3	10	7171056	7172158	2	SFMBT2	I2E3	0.427378518	0.034192716	0.423155095	0.021030283	19
513	2	SFMBT2	E3I3	10	7171894	7172494	2	SFMBT2	E3I3	0.788497546	0.885638778	0.80195337	0.781615856	17
514	2	SFMBT2	I3E4	10	7172158	7172661	2	SFMBT2	I3E4	0.764417767	0.725226951	0.783945653	0.544368361	11
515	2	SFMBT2	E4I4	10	7172494	7175989	2	SFMBT2	E4I4	0.044628964	0.076208671	0.066703524	0.047433136	50
516	2	SFMBT2	I4E5	10	7172661	7176165	2	SFMBT2	I4E5	0.037788664	0.049436463	0.058583472	0.018696298	47
517	2	SFMBT2	E5I5	10	7175989	7188623	2	SFMBT2	E5I5	0.613995829	0.478092573	0.480778093	0.261792501	234
518	2	SFMBT2	I5E6	10	7176165	7188733	2	SFMBT2	I5E6	0.605902272	0.528699603	0.474898165	0.291523029	235
519	2	SFMBT2	E6I6	10	7188623	7197547	2	SFMBT2	E6I6	0.376439314	0.570647242	0.495164658	0.495164658	209
520	2	SFMBT2	I6E7	10	7188733	7197687	2	SFMBT2	I6E7	0.37607311	0.569843059	0.495134642	0.495134642	209
521	2	SFMBT2	E7I7	10	7197547	7200413	2	SFMBT2	E7I7	0.411703401	0.075805	0.424476233	0.144016618	51
522	2	SFMBT2	I7E8	10	7197687	7200484	2	SFMBT2	I7E8	0.352256141	0.22722098	0.366832549	0.329483485	51
523	2	SFMBT2	E8I8	10	7200413	7202479	2	SFMBT2	E8I8	0.395056557	0.606860262	0.432009657	0.432009657	49
524	2	SFMBT2	I8E9	10	7200484	7202522	2	SFMBT2	I8E9	0.548751048	0.771389578	0.567056373	0.567056373	49
525	2	SFMBT2	E9I9	10	7202479	7205814	2	SFMBT2	E9I9	0.163615117	0.232268356	0.168467204	0.112742726	68
526	2	SFMBT2	I9E10	10	7202522	7205928	2	SFMBT2	I9E10	0.161613271	0.221306446	0.17038787	0.110312637	67
527	2	SFMBT2	E10I10	10	7205814	7220410	2	SFMBT2	E10I10	0.021909399	0.000819344	0.059238068	0.00161471	329
528	2	SFMBT2	I10E11	10	7205928	7220537	2	SFMBT2	I10E11	0.021909399	0.000819344	0.059421981	0.001357089	329
529	2	SFMBT2	E11I11	10	7220410	7227854	2	SFMBT2	E11I11	0.091299755	0.062198678	0.139893121	0.067431718	141
530	2	SFMBT2	I11E12	10	7220537	7227937	2	SFMBT2	I11E12	0.091299755	0.062198678	0.142953498	0.067269068	141
531	2	SFMBT2	E12I12	10	7227854	7243557	2	SFMBT2	E12I12	0.108547207	0.102981346	0.190125831	0.148025597	278
532	2	SFMBT2	I12E13	10	7227937	7243705	2	SFMBT2	I12E13	0.109353151	0.110660796	0.187468111	0.154398731	280
533	2	SFMBT2	E13I13	10	7243557	7248547	2	SFMBT2	E13I13	0.006636902	0.002283625	0.022065175	0.003795401	103
534	2	SFMBT2	I13E14	10	7243705	7248649	2	SFMBT2	I13E14	0.006715092	0.002516375	0.024130786	0.005738323	105
535	2	SFMBT2	E14I14	10	7248547	7276891	2	SFMBT2	E14I14	0.051798733	0.024297549	0.022749066	0.008859522	613
536	2	SFMBT2	I14E15	10	7248649	7276989	2	SFMBT2	I14E15	0.051116764	0.022044856	0.020756867	0.006542449	609
537	2	SFMBT2	E15I15	10	7276891	7283903	2	SFMBT2	E15I15	0.048963257	0.083208236	0.024187104	0.024187104	127
538	2	SFMBT2	I15E16	10	7276989	7284150	2	SFMBT2	I15E16	0.051382217	0.087256017	0.024997615	0.024997615	131
539	2	SFMBT2	E16I16	10	7283903	7285865	2	SFMBT2	E16I16	0.217287053	0.347765626	0.021693231	0.021693231	31
540	2	SFMBT2	I16E17	10	7284150	7285954	2	SFMBT2	I16E17	0.18601392	0.302802296	0.02268509	0.02268509	27
541	2	SFMBT2	E17I17	10	7285865	7367648	2	SFMBT2	E17I17	0.222376831	0.320908434	0.013487198	0.035653174	1,437
542	2	SFMBT2	I17E18	10	7285954	7367889	2	SFMBT2	I17E18	0.222438808	0.319829137	0.013271527	0.078679116	1,444
543	2	SFMBT2	E18I18	10	7367648	7370280	2	SFMBT2	E18I18	0.171665571	0.286718471	0.092778448	0.092778448	37
544	2	SFMBT2	I18E19	10	7367889	7370375	2	SFMBT2	I18E19	0.133298048	0.226227808	0.077010244	0.077010244	33
545	2	SFMBT2	E19I19	10	7370280	7381798	2	SFMBT2	E19I19	0.304265195	0.484179803	0.159515227	0.159515227	202
546	2	SFMBT2	I19E20	10	7370375	7381949	2	SFMBT2	I19E20	0.337279386	0.527964015	0.175272778	0.175272778	201
547	2	SFMBT2	E20I20	10	7381798	7411344	2	SFMBT2	E20I20	0.758155185	0.765095481	0.349796484	0.192869833	629
548	2	SFMBT2	I20E21	10	7381949	7411490	2	SFMBT2	I20E21	0.757569867	0.762032545	0.346062388	0.18221414	627
549	2	FANK1	E1I1	10	125896563	125980160	2	FANK1	E1I1	0.386510658	0.582282131	0.514511881	0.587810007	1,562
34	2	FANK1	ALL	10	125896564	126009592	2	FANK1	ALL	0.577490208	0.776953902	0.705593621	0.705593621	2,149
550	2	FANK1	I1E2	10	125896655	125980338	2	FANK1	I1E2	0.454888518	0.56508305	0.5475246	0.600435487	1,550
551	2	FANK1	E2I2	10	125980160	125988550	2	FANK1	E2I2	0.806352788	0.751980279	0.942070656	0.845281781	183
552	2	FANK1	I2E3	10	125980338	125988675	2	FANK1	I2E3	0.754812375	0.766752167	0.920546757	0.89432522	180
553	2	FANK1	E3I3	10	125988550	125989279	2	FANK1	E3I3	0.672462653	0.775116243	0.910421533	0.910421533	20
554	2	FANK1	I3E4	10	125988675	125989357	2	FANK1	I3E4	0.801965751	1	0.982309827	0.949432322	21
555	2	FANK1	E4I4	10	125989279	125995416	2	FANK1	E4I4	0.820600476	1	0.81192914	0.941603972	136
556	2	FANK1	I4E5	10	125989357	125995498	2	FANK1	I4E5	0.810107832	1	0.800754138	0.800754138	134
557	2	FANK1	E5I5	10	125995416	125996549	2	FANK1	E5I5	0.633355119	0.762795682	0.562157625	0.552664888	13

558	2	FANK1	I5E6	10	125995498	125996624	2	FANK1	I5E6	0.633355119	0.762795682	0.564082264	0.558639915	13
559	2	FANK1	E6I6	10	125996549	125997419	2	FANK1	E6I6	0.27221746	0.419226258	0.552939154	0.552939154	13
560	2	FANK1	I6E7	10	125996624	125997485	2	FANK1	I6E7	0.253603976	0.401208995	0.480990859	0.480990859	14
561	2	FANK1	E7I7	10	125997419	126004883	2	FANK1	E7I7	0.652953934	0.833134184	0.630503788	0.630503788	145
562	2	FANK1	I7E8	10	125997485	126005049	2	FANK1	I7E8	0.733099089	0.867533372	0.687606672	0.635140462	148
563	2	FANK1	E8I8	10	126004883	126008406	2	FANK1	E8I8	0.630924256	0.818818566	0.596943389	0.596943389	57
564	2	FANK1	I8E9	10	126005049	126008550	2	FANK1	I8E9	0.527114053	0.725983935	0.506625764	0.506625764	54
565	2	FANK1	E9I9	10	126008406	126009053	2	FANK1	E9I9	0.129132375	0.1777252	0.169289683	0.229447909	12
566	2	FANK1	I9E10	10	126008550	126009131	2	FANK1	I9E10	0.129132375	0.1777252	0.174221263	0.234776297	11
567	2	FANK1	E10I10	10	126009053	126009221								2
568	2	FANK1	I10E11	10	126009131	126009266								2
569	2	FANK1	E11I11	10	126009221	126009372								NA
570	2	FANK1	I11E12	10	126009266	126009592	2	FANK1	I11E12	0.120202379	0.141387496	0.078371506	0.06111318	7
657	2	PPP2R5E	E1I1	14	63371363	63382055	2	PPP2R5E	E1I1	0.421826548	0.625987118	0.336650534	0.336650534	188
35	2	PPP2R5E	ALL	14	63371364	63543377	2	PPP2R5E	ALL	0.83616388	0.840358639	0.014750934	0.066396699	3,199
658	2	PPP2R5E	I1E2	14	63376108	63382157	2	PPP2R5E	I1E2	0.35137433	0.535427689	0.336495691	0.336495691	113
659	2	PPP2R5E	E2I2	14	63382055	63384443	2	PPP2R5E	E2I2	0.397013842	0.245263384	0.42632313	0.240920573	29
660	2	PPP2R5E	I2E3	14	63382157	63384571	2	PPP2R5E	I2E3	0.394784802	0.258860871	0.418238679	0.260392144	30
661	2	PPP2R5E	E3I3	14	63384443	63389611	2	PPP2R5E	E3I3	0.101514085	0.172313808	0.121315648	0.121315648	112
662	2	PPP2R5E	I3E4	14	63384571	63389731	2	PPP2R5E	I3E4	0.095237144	0.161858391	0.11786325	0.11786325	111
663	2	PPP2R5E	E4I4	14	63389611	63391816	2	PPP2R5E	E4I4	0.01546655	0.029427092	0.041811588	0.041811588	36
664	2	PPP2R5E	I4E5	14	63389731	63391867	2	PPP2R5E	I4E5	0.017296843	0.032694996	0.04439383	0.04439383	35
665	2	PPP2R5E	E5I5	14	63391816	63391971	2	PPP2R5E	E5I5	0.63145329	0.452709749	0.815196759	0.408729016	3
666	2	PPP2R5E	I5E6	14	63391867	63392025	2	PPP2R5E	I5E6	0.711726312	0.353718792	0.863145457	0.242150842	4
667	2	PPP2R5E	E6I6	14	63391971	63393819	2	PPP2R5E	E6I6	0.369292165	0.573272322	0.453442007	0.453442007	31
668	2	PPP2R5E	I6E7	14	63392025	63393928	2	PPP2R5E	I6E7	0.383551533	0.592911928	0.477385177	0.477385177	33
669	2	PPP2R5E	E7I7	14	63393819	63395225	2	PPP2R5E	E7I7	0.827002337	1	0.932377302	0.932377302	33
670	2	PPP2R5E	I7E8	14	63393928	63395285	2	PPP2R5E	I7E8	0.815149328	1	0.930923462	0.930923462	31
671	2	PPP2R5E	E8I8	14	63395225	63396585	2	PPP2R5E	E8I8	1	1	0.914354987	1	21
672	2	PPP2R5E	I8E9	14	63395285	63396716	2	PPP2R5E	I8E9	0.776529125	0.555764103	0.76082067	0.465674794	21
673	2	PPP2R5E	E9I9	14	63396585	63415139	2	PPP2R5E	E9I9	0.734345666	0.639230137	0.714252621	0.717408399	303
674	2	PPP2R5E	I9E10	14	63396716	63415232	2	PPP2R5E	I9E10	0.738177345	0.64802718	0.715274093	0.739840516	303
675	2	PPP2R5E	E10I10	14	63415139	63421992	2	PPP2R5E	E10I10	0.864150833	1	0.916224871	0.916224871	117
676	2	PPP2R5E	I10E11	14	63415232	63422094	2	PPP2R5E	I10E11	0.861089389	1	0.868441722	0.921948149	116
677	2	PPP2R5E	E11I11	14	63421992	63453688	2	PPP2R5E	E11I11	0.803328387	1	0.017499905	0.017499905	544
678	2	PPP2R5E	I11E12	14	63422094	63453885	2	PPP2R5E	I11E12	0.804235447	1	0.015358004	0.015358004	545
679	2	PPP2R5E	E12I12	14	63453688	63539528	2	PPP2R5E	E12I12	0.889361586	0.671881208	0.007993295	0.02456488	1,722
680	2	PPP2R5E	I12E13	14	63453885	63539689	2	PPP2R5E	I12E13	0.889277895	0.671169492	0.009117851	0.025417297	1,722
681	2	PPP2R5E	E13I13	14	63539528	63542778	2	PPP2R5E	E13I13	0.933310012	0.132891769	0.26848691	0.071087747	51
682	2	PPP2R5E	I13E14	14	63539689	63543377	2	PPP2R5E	I13E14	0.863870788	0.047777178	0.21609812	0.022744427	59
36	2	GSTTP2	ALL	22	24451096	24467071	2	GSTTP2	ALL	0.917721891	1	0.989577505	0.734956079	237

SKAT = using rare SNPS (MAF<0.02) in the region
SKAT = Optimal Unified Test (SKAT-O) using rare SNPs in the region
RareComm = SKAT using both rare and common SNPS in the region
RareCommO = Optimal Unified Test (SKAT-O) using rare and common SNPs in the region

green = p-values <0.01
yellow = 0.05 < p-value < 0.01

Supplementary Table 5a SNPs from WGS association analysis for IVIG refractory used in positional and functional (eQTL and chromatin interactions) mapping

uniqID : Unique ID of SNPs consists of chr:position:allele1:allele2 where alleles are alphabetically ordered.
rsID : rsID of SNPs as provided in the input GWAS, otherwise extracted from the specified reference panel.
chr : chromosome
pos : position on hg19
effect_allele : Effect/risk allele if it is provided in the input GWAS summary statistics file. If not, this is the alternative (minor) allele in the reference panel.
non_effect_allele : Non-effect/non-risk allele if it is provided in the input GWAS summary statistics file. If not, this is the reference (major) allele in the reference panel.
MAF : Minor allele frequency computed based on the reference panel.
gwasP : P-value provided in the input GWAS summary statistics file. Non-GWAS tagged SNPs (which do not exist in input file but are extracted from the reference panel) have "NA" instead.
or : Odds ratio provided in the input GWAS summary statistics file if available. Non-GWAS tagged SNPs (which do not exist in input file but are extracted from the reference panel) have "NA" instead.
beta : Beta provided in the input GWAS summary statistics file if available. Non-GWAS tagged SNPs (which do not exist in input file but are extracted from the reference panel) have "NA" instead.
se : Standard error provided in the input GWAS summary statistics file if available. Non-GWAS tagged SNPs (which do not exist in input file but are extracted from the reference panel) have "NA" instead.
r2 : The maximum r2 of the SNP with one of the independent significant SNP (this doesn't have to be top lead SNPs in the genomic loci).
IndSigSNP : rsID of a independent significant SNP which has the maximum r2 of the SNP.
Genomic locus : Index of the genomic risk loci matching with "GenomicRiskLoci.txt".
nearestGene : The nearest Gene of the SNP based on ANNOVAR annotations.
Note that ANNOVAR annotates "consequence" function by prioritising the most deleterious annotation for SNPs which are locating a genomic region where multiple genes are overlapped.
Genes are encoded in symbol, if it is available, otherwise Ensembl ID.
dist : Distance to the nearest gene. SNPs which are locating in the gene body or 1kb up- or down-stream of TSS or TES have 0.
func : Functional consequence of the SNP on the gene obtained from ANNOVAR. For exonic SNPs, detail annotation (e.g. non-synonymous, stop gain and so on) is available in ANNOVAR table (annov.txt).
CADD : CADD score which is computed based on 63 annotations. 'NA' if not available.
RDB : RegulomeDB score which is the categorical score (from 1a to 7). 1a is the highest score that the SNP has the most biological evidence to be regulatory element.
minChrState : The minimum 15-core chromatin state across 127 tissue/cell type.
commonChrState : The most common 15-core chromatin state across 127 tissue/cell types.
posMapFilt : Whether the SNP was used for positional mapping or not. 1 is used, otherwise 0. When positional mapping is not performed, all SNPs have 0.
eqtlMapFilt : Whether the SNP was used for eQTL mapping or not. 1 is used, otherwise 0. When eQTL mapping is not performed, all SNPs have 0.
ciMapFilt : Whether the SNP was used for chromatin interaction mapping or not. 1 is used, otherwise 0. When chromatin interaction mapping is not performed, all SNPs have 0.

uniqID	rsID	chr	pos	non_effect	effect_allel	MAF	gwasP	beta	r2	IndSigSNP	GenomicLc	nearestGer	dist	func	CADD	RDB	minChrStat	commonCh	posMapFilt	eqtlMapFil	ciMapFilt
9:7594660	rs1237839	9	7594660	T	A	0.247	7.23E-06	0.7586	1	rs1237839	1	RP11-366I	3162	intergenic	3.361	6	9	15	0	0	1
9:7595510	rs2171008	9	7595510	C	G	0.2518	1.67E-05	0.7226	0.921147	rs1237839	1	RP11-366I	2312	intergenic	2.514	6	9	15	0	0	1
9:7598529	rs1012007	9	7598529	C	G	0.251	9.43E-06	0.7409	0.888726	rs1237839	1	RP11-366I	151	downstrea	1.445	7	9	15	0	0	1
17:260167	rs5661406	17	26016701	G	C	0.0002	NA	NA	1	rs7932762	2	RP11-19P2	5530	intergenic	0.825	NA	9	15	0	0	1
17:264971	rs5313018	17	26497109	ATGCTGG	A	0.000998	NA	NA	1	rs7932762	2	NLK	0	intronic	NA	NA	4	5	1	0	1
17:264971	rs5379119	17	26497109	A	T	0.0002	NA	NA	1	rs7932762	2	NLK	0	NA	2.408	NA	4	5	1	0	1
17:266596	rs1431492	17	26659645	G	A	0.0002	NA	NA	1	rs7932762	2	IFT20	0	intronic	4.857	6	4	5	1	0	1
17:266984	rs5529925	17	26698400	G	A	0.0002	NA	NA	1	rs7932762	2	TMEM199:	0:0:0:0	ncRNA_int	3.378	NA	1	2	1	0	1
17:269141	rs1411133	17	26914116	A	G	0.0002	NA	NA	1	rs7932762	2	RP11-192H	0:00	intronic	7.7	6	4	5	1	0	1
17:269494	rs7932762	17	26949490	T	A	0.0002	4.93E-06	-1.073	1	rs7932762	2	KIAA0100	0	intronic	4.371	5	4	4	1	0	1
17:270471	rs1421097	17	27047194	G	A	0.0002	NA	NA	1	rs7932762	2	RPL23A	0	UTR5	6.422	4	1	1	1	0	1
17:272796	rs1848845	17	27279679	T	C	0.0002	NA	NA	1	rs7932762	2	PIPOX	0	upstream	7.352	4	1	1	1	0	1
17:273644	rs5618125	17	27364414	G	T	0.0002	NA	NA	1	rs7932762	2	PIPOX	0	intergenic	4.763	NA	2	14	1	0	1
17:274297	rs1472400	17	27429704	G	A	0.0002	NA	NA	1	rs7932762	2	MYO18A	0	intronic	4.51	5	4	4	1	0	1
17:274543	rs5287594	17	27454312	C	T	0.0002	NA	NA	1	rs7932762	2	MYO18A	0	intronic	0.636	NA	4	5	1	0	1
17:275038	rs5651078	17	27503872	C	T	0.0002	NA	NA	1	rs7932762	2	MYO18A	0	intronic	8.583	NA	1	1	1	0	1
17:277419	rs5682610	17	27741907	T	C	0.0002	NA	NA	1	rs7932762	2	TAOK1	0	intronic	3.127	NA	4	5	1	0	0
17:278289	rs1998622	17	27828950	G	A	0.0002	NA	NA	1	rs7932762	2	TAOK1	0	intronic	14.68	5	4	4	1	0	1

Supplementary Table 5b Prioirtized genes identified from WGS association analysis for IVIG refractory by positional and functional (eQTL and chromatin interactions) mapping

ensg : ENSG ID
symbol : Gene Symbol
chr : chromosome
start : Starting position of the gene
end : Ending position of the gene
strand : Strand of the gene
type : Gene biotype from Ensembl
entrezID : entrez ID (if available)
HUGO : HUGO (HGNC) gene symbol
pLI : pLI score from ExAC database. The probability of being loss-of-function intolerant. The higher the score is, the more intolerant to loss-of-function mutations the gene is.
ncRVIS : Non-coding residual variation intolerance score. The higher the score is, the more intolerant to non-coding variation the gene is.
posMapSNPs (posMap): Number of SNPs mapped to gene based on positional mapping (after functional filtering if parameters are given).
posMapMaxCADD (posMap): The maximum CADD score of mapped SNPs by positional mapping.
eqtlMapSNPs (eqtlMap): Number of SNPs mapped to the gene based on eQTL mapping.
eqtlMapminP (eqtlMap): The minimum eQTL P-value of mapped SNPs.
eqtlMapminQ (eqtlMap): The minimum eQTL FDR of mapped SNPs.
eqtlMapts (eqtlMap): Tissue types of mapped eQTL SNPs.
eqtlDirection (eqtlMap): Consequential direction of mapped eQTL SNPs after aligning risk increasing alleles in GWAS and tested alleles in eQTL data source.
NA if risk increase alleles are not defined either because signed effect is not provided in the input GWAS file or all of eQTL SNPs are not in the input GWAS file but included from reference panel for the gene.
ciMap (ciMap): "Yes" if the gene is mapped by chromatin interaction mapping, "No" otherwise.
ciMapts (ciMap): Tissue/cell types of mapped chromatin interactions.
minGwasP : The minimum P-value of mapped SNPs.
IndSigSNPs : rsID of the independent significant SNPs that are in LD with the mapped SNPs.
Note that this column does not represent SNPs that are mapped to the gene.
The actual mapped SNPs are available in gene_snps.txt file.
GenomicLocus : Index of genomic loci where mapped SNPs are from. Multiple loci can be assigned with ":" delimiter.

ensg	symbol	chr	start	end	strand	type	entrezID	HUGO	pLI	ncRVIS	posMapSNPs	posMapMaxCADD	eqtlMapSNPs	qtlMapmin	qtlMapmin	eqtlMapts	qtlDirectio	ciMap	ciMapts	minGwasP	IndSigSNPs	enomicLocus
ENSG0000(	TMEM261	9	7796490	7888380	-1	protein_co	90871	TMEM261	0.000778	1.276229	0	0	0	NA	NA	NA	NA	Yes	Mesenchymal_St	7.23E-06	rs1237839	1
ENSG0000(	WSB1	17	25621102	25640657	1	protein_co	26118	WSB1	0.923194	-0.81155	0	0	0	NA	NA	NA	NA	Yes	IMR90:Mesenchy	NA	rs7932762	2
ENSG0000(	AC015688.	17	25950390	25967803	1	protein_co	NA	NA	NA	NA	0	0	0	NA	NA	NA	NA	Yes	IMR90:hESC	NA	rs7932762	2
ENSG0000(	LGALS9	17	25956824	25976586	1	protein_co	3965	LGALS9	0.001391	0.597683	0	0	0	NA	NA	NA	NA	Yes	IMR90:hESC	NA	rs7932762	2
ENSG0000(	NOS2	17	26083792	26127525	-1	protein_co	4843	NOS2	7.26E-09	0.469983	0	0	0	NA	NA	NA	NA	Yes	Fetal_Cortex:Aort	NA	rs7932762	2
ENSG0000(	RP1-66C13	17	26125832	26220391	-1	protein_co	NA	NA	NA	NA	0	0	0	NA	NA	NA	NA	Yes	Adult_Cortex:Fet	NA	rs7932762	2
ENSG0000(	LYRM9	17	26205340	26221778	-1	protein_co	201229	LYRM9	0.045	-0.7553	0	0	0	NA	NA	NA	NA	Yes	Adult_Cortex:Fet	NA	rs7932762	2
ENSG0000(	NLK	17	26368763	26523407	1	protein_co	51701	NLK	0.998934	-1.1514	2	2.408	0	NA	NA	NA	NA	Yes	Aorta:Left_Ventri	NA	rs7932762	2
ENSG0000(	AC061975.	17	26638667	26642323	1	protein_co	NA	NA	NA	NA	0	0	0	NA	NA	NA	NA	Yes	Left_Ventricle:IM	NA	rs7932762	2
ENSG0000(	TMEM97	17	26646121	26655707	1	protein_co	27346	TMEM97	0.028406	-1.00259	1	4.857	0	NA	NA	NA	NA	Yes	IMR90:Mesenchy	NA	rs7932762	2
ENSG0000(	IFT20	17	26655352	26662515	-1	protein_co	90410	IFT20	0.482558	-0.52329	1	4.857	0	NA	NA	NA	NA	Yes	Promoter_anchor	NA	rs7932762	2
ENSG0000(	TNFAIP1	17	26662628	26674035	1	protein_co	7126	TNFAIP1	0.007362	-1.38635	1	4.857	0	NA	NA	NA	NA	Yes	Promoter_anchor	NA	rs7932762	2
ENSG0000(	POLDIP2	17	26673659	26684545	-1	protein_co	26073	POLDIP2	0.049245	NA	0	0	0	NA	NA	NA	NA	Yes	IMR90:Mesenchy	NA	rs7932762	2
ENSG0000(	TMEM199	17	26684604	26708716	1	protein_co	147007	TMEM199	0.000654	-0.45221	1	3.378	0	NA	NA	NA	NA	Yes	IMR90:Mesenchy	NA	rs7932762	2
ENSG0000(	VTN	17	26691290	26700110	-1	protein_co	7448	VTN	1.14E-09	0.058431	1	3.378	0	NA	NA	NA	NA	Yes	Adult_Cortex:Fet	4.93E-06	rs7932762	2
ENSG0000(	CTB-96E2.2	17	26691310	26695033	-1	protein_co	NA	NA	NA	NA	1	3.378	0	NA	NA	NA	NA	Yes	IMR90:Mesenchy	NA	rs7932762	2
ENSG0000(	SARM1	17	26691378	26728065	1	protein_co	23098	SARM1	9.19E-09	NA	1	3.378	0	NA	NA	NA	NA	Yes	IMR90:Mesenchy	NA	rs7932762	2
ENSG0000(	SLC46A1	17	26721661	26734215	-1	protein_co	113235	SLC46A1	0.104423	NA	0	0	0	NA	NA	NA	NA	Yes	IMR90:Mesendo	NA	rs7932762	2
ENSG0000(	RP11-192H	17	26782770	26941215	-1	protein_co	NA	NA	NA	NA	2	7.7	0	NA	NA	NA	NA	Yes	IMR90:Mesenchy	4.93E-06	rs7932762	2
ENSG0000(	SLC13A2	17	26800311	26824799	1	protein_co	9058	SLC13A2	8.29E-07	-0.15257	0	0	0	NA	NA	NA	NA	Yes	GM12878:IMR90	4.93E-06	rs7932762	2
ENSG0000(	FOXN1	17	26833261	26865914	1	protein_co	8456	FOXN1	0.969498	0.179225	0	0	0	NA	NA	NA	NA	Yes	GM12878:IMR90	4.93E-06	rs7932762	2
ENSG0000(	PIGS	17	26880401	26898890	-1	protein_co	94005	PIGS	0.079952	-1.05496	0	0	0	NA	NA	NA	NA	Yes	IMR90:Mesenchy	4.93E-06	rs7932762	2
ENSG0000(	ALDOC	17	26900133	26904282	-1	protein_co	230	ALDOC	0.790721	-0.44703	1	7.7	0	NA	NA	NA	NA	Yes	IMR90:Mesenchy	4.93E-06	rs7932762	2
ENSG0000(	SPAG5	17	26904588	26926297	-1	protein_co	10615	SPAG5	7.26E-06	-0.63518	1	7.7	0	NA	NA	NA	NA	Yes	Adult_Cortex:IM	NA	rs7932762	2
ENSG0000(	SGK494	17	26934982	26941218	-1	protein_co	124923	SGK494	2.61E-05	NA	1	4.371	0	NA	NA	NA	NA	Yes	IMR90:Mesenchy	4.93E-06	rs7932762	2
ENSG0000(	KIAA0100	17	26941458	26972472	-1	protein_co	9703	KIAA0100	0.265244	-0.49591	1	4.371	0	NA	NA	NA	NA	Yes	Adult_Cortex:Fet	4.93E-06	rs7932762	2
ENSG0000(	SDF2	17	26975374	26989207	-1	protein_co	6388	SDF2	0.68281	-0.68777	0	0	0	NA	NA	NA	NA	Yes	Promoter_anchor	4.93E-06	rs7932762	2
ENSG0000(	SUPT6H	17	26989109	27029697	1	protein_co	6830	SUPT6H	1	-1.01782	0	0	0	NA	NA	NA	NA	Yes	Promoter_anchor	4.93E-06	rs7932762	2
ENSG0000(	PROCA1	17	27030215	27038872	-1	protein_co	147011	PROCA1	0.000876	-0.63344	1	6.422	0	NA	NA	NA	NA	Yes	Adult_Cortex:IM	4.93E-06	rs7932762	2
ENSG0000(	RAB34	17	27041299	27045447	-1	protein_co	83871	RAB34	4.68E-06	-0.41151	1	6.422	0	NA	NA	NA	NA	Yes	Promoter_anchor	NA	rs7932762	2
ENSG0000(	RPL23A	17	27046411	27051377	1	protein_co	6147	RPL23A	0.876904	-1.22697	1	6.422	0	NA	NA	NA	NA	Yes	Promoter_anchor	NA	rs7932762	2
ENSG0000(	TLCD1	17	27051366	27054953	-1	protein_co	116238	TLCD1	0.01115	NA	1	6.422	0	NA	NA	NA	NA	Yes	Promoter_anchor	NA	rs7932762	2
ENSG0000(	NEK8	17	27052915	27070473	1	protein_co	284086	NEK8	1.56E-06	-0.50158	1	6.422	0	NA	NA	NA	NA	Yes	Promoter_anchor	NA	rs7932762	2
ENSG0000(	TRAF4	17	27071002	27077974	1	protein_co	9618	TRAF4	0.840039	-0.45882	0	0	0	NA	NA	NA	NA	Yes	Adult_Cortex:Lef	NA	rs7932762	2
ENSG0000(	FAM222B	17	27082996	27182250	-1	protein_co	55731	FAM222B	0.549447	-1.29497	0	0	0	NA	NA	NA	NA	Yes	Fetal_Cortex:Live	NA	rs7932762	2
ENSG0000(	ERAL1	17	27181956	27188085	1	protein_co	26284	ERAL1	1.04E-06	0.014401	0	0	0	NA	NA	NA	NA	Yes	Fetal_Cortex:Live	NA	rs7932762	2
ENSG0000(	FLOT2	17	27206353	27224697	-1	protein_co	2319	FLOT2	0.017234	-0.08168	0	0	0	NA	NA	NA	NA	Yes	Adult_Cortex:Fet	NA	rs7932762	2
ENSG0000(	DHRS13	17	27224799	27230089	-1	protein_co	147015	DHRS13	0.000234	-0.21991	0	0	0	NA	NA	NA	NA	Yes	Adult_Cortex:Fet	NA	rs7932762	2

ENSG0000(	PHF12	17	27232268	27278789	-1	protein_co	57649	PHF12	0.99996	NA	1	7.352	0	NA	NA	NA	NA	Yes	Promoter_ancho	4.93E-06	rs7932762	2
ENSG0000(	PIPOX	17	27277531	27384234	1	protein_co	51268	PIPOX	9.88E-11	0.024004	2	7.352	0	NA	NA	NA	NA	Yes	Promoter_ancho	4.93E-06	rs7932762	2
ENSG0000(	SEZ6	17	27281919	27333458	-1	protein_co	124925	SEZ6	NA	-0.40724	1	7.352	0	NA	NA	NA	NA	Yes	Adult_Cortex:Fet	NA	rs7932762	2
ENSG0000(	MYO18A	17	27400528	27507430	-1	protein_co	399687	MYO18A	0.999418	NA	3	8.583	0	NA	NA	NA	NA	Yes	IMR90	NA	rs7932762	2
ENSG0000(	TIAF1	17	27400537	27418537	-1	protein_co	9220	TIAF1	0.026394	-1.09167	0	0	0	NA	NA	NA	NA	Yes	Liver:IMR90:Mes	NA	rs7932762	2
ENSG0000(	CRYBA1	17	27573881	27581512	1	protein_co	1411	CRYBA1	0.000722	-0.21331	0	0	0	NA	NA	NA	NA	Yes	Left_Ventricle:Liv	NA	rs7932762	2
ENSG0000(	NUFIP2	17	27582854	27621136	-1	protein_co	57532	NUFIP2	0.992086	-2.76185	0	0	0	NA	NA	NA	NA	Yes	Left_Ventricle:IM	NA	rs7932762	2
ENSG0000(	TAOK1	17	27717482	27878922	1	protein_co	57551	TAOK1	1	-2.55769	2	14.68	0	NA	NA	NA	NA	Yes	Mesenchymal_St	NA	rs7932762	2
ENSG0000(	CORO6	17	27941774	27949925	-1	protein_co	84940	CORO6	3.14E-05	-0.49879	0	0	0	NA	NA	NA	NA	Yes	Fetal_Cortex	NA	rs7932762	2
ENSG0000(	CPD	17	28705923	28797007	1	protein_co	1362	CPD	0.247132	-1.13936	0	0	0	NA	NA	NA	NA	Yes	Fetal_Cortex	NA	rs7932762	2
ENSG0000(	RAB11FIP4	17	29718642	29865236	1	protein_co	84440	RAB11FIP4	0.993664	4.272929	0	0	0	NA	NA	NA	NA	Yes	IMR90	NA	rs7932762	2
ENSG0000(	AC003101.	17	29898161	29902678	1	protein_co	NA	NA	0.113322	NA	0	0	0	NA	NA	NA	NA	Yes	IMR90	NA	rs7932762	2

Supplementary Table 6a Combined and Race-specific Analysis results for SNPs in genes previously associated with IVIG response

Ord	Gene	SNP	Chr	Pos	Comb_P	CAU_P	AA_P	AsPI_P	Hisp_P
1	HMGB1	rs71093072	13	30491660	NA	NA	NA	NA	NA
2	MMP3	rs650108	11	102838056	0.182	0.2075	0.4947	0.2972	0.9742
3	ITPR3	rs3831079	6	33657823	0.4022	0.6568	0.6667	0.5945	0.8361
4	HMGB1	rs34700691	13	30485545	0.01876	0.2276	0.0528	0.1329	0.3582
5	FCGR2A	rs6427598	1	161514774	0.4303	0.9852	NA	NA	0.83
6	MMP3	C11_M625011	11	102838730	0.03197	0.7124	0.7576	0.02302	0.2698
7	FCGR2A	C1_161515309	1	161515309	NA	NA	NA	NA	NA
8	TGF-bR2	C3_M151187	3	30673572	0.005937	0.07361	0.3564	0.03019	0.1282
9	TGF-bR2	rs1461082	3	30660114	0.001917	0.0543	0.1561	0.004372	0.6066
10	ITPR3	rs1570759	6	33666163	0.2171	0.5984	0.7389	0.5911	0.4664
11	TGF-bR2	rs3773635	3	30658940	0.0334	0.3317	0.4101	0.02592	0.3031
12	ITPR3	rs6901411	6	33665750	0.5921	0.9314	0.6775	0.6193	0.3884
13	FCGR2A	rs6681063	1	161514511	0.4971	0.9853	0.1947	0.6142	0.6498
14	TGF-bR2	rs3773644	3	30670852	0.004229	0.06888	0.3564	0.0284	0.1336
15	TGF-bR2	rs3773647	3	30674767	0.01347	0.05385	0.6088	0.1169	0.1617
16	TGF-bR2	rs3773634	3	30656444	0.04528	0.3138	0.4907	0.04126	0.3031
17	ITPR3	rs2296337	6	33663919	0.5247	0.9485	0.6775	0.4623	0.5794
18	HMGB1	rs35962852	13	30505340	0.08417	0.3992	0.4669	0.02437	0.8555
19	ITPR3	rs9366825	6	33650408	0.06653	0.6727	0.4002	0.2086	0.1995
20	HMGB1	rs17074652	13	30484947	0.1023	0.2459	0.9759	0.08716	0.5927
21	HMGB1	rs35193740	13	30485416	0.111	0.2482	0.9759	0.1329	0.5927
22	ITPR3	rs2296343	6	33658940	0.8519	0.7201	0.4019	0.6004	0.5273
23	FCGR2A	C1_161514865	1	161514865	0.0288	0.2449	NA	0.4579	0.261
24	FCGR2A	rs4233372	1	161513144	NA	NA	NA	NA	NA
25	TGF-bR2	rs17025791	3	30632187	0.6018	NA	0.3366	NA	0.449
26	TGF-bR2	rs11466489	3	30633492	0.595	NA	0.3366	NA	0.449
27	TGF-bR2	rs11466490	3	30633541	0.595	NA	0.3366	NA	0.449
28	HMGB1	C13_M684757	13	30483356	0.3124	0.2791	0.9813	0.5091	0.9392
29	ITPR3	rs9394158	6	33645590	0.08808	0.7456	0.5017	0.2086	0.1995
30	TGF-bR2	rs9831477	3	30652030	0.1133	0.2145	0.6828	0.9598	0.2028
31	ITPR3	C6_M380378	6	33662387	0.6113	0.9438	0.2527	0.4623	0.5794
32	KCNN2	rs11745974	5	114491042	0.808	0.5216	0.2513	0.625	0.9215
33	ITPR3	rs12662536	6	33667630	0.7715	0.8429	0.3632	0.5327	0.5794
34	ITPR3	rs2229634	6	33670403	0.4936	0.9719	0.5985	0.5219	0.5229
35	HMGB1	rs9578178	13	30495833	0.0209	0.6073	0.024	0.1095	0.06355
36	HMGB1	rs77800880	13	30597158	0.06651	0.2719	0.5172	0.07076	0.7391
37	HMGB1	rs12431028	13	30600091	0.06651	0.2719	0.5172	0.07076	0.7391
38	FCGR2A	rs1050399	1	161518447	0.06881	0.03614	0.8524	0.5838	0.8672
39	ITPR3	rs1408711	6	33652231	0.05838	0.6802	0.7415	0.2086	0.09387
40	MMP3	rs679620	11	102842889	0.4371	0.5638	0.9495	0.1251	0.7118
41	ITPR3	rs9368771	6	33673944	0.5843	0.8895	0.2816	0.5849	0.468
42	KCNN2	rs10050725	5	114443724	0.4967	0.3025	0.0844	0.1422	0.1447
43	MMP3	rs639752	11	102836608	0.5849	0.5984	0.6613	0.1298	0.4901
44	MMP3	rs575027	11	102837183	0.6222	0.634	0.6613	0.1298	0.4901
45	TGF-bR2	rs11466481	3	30622656	0.9577	NA	0.9491	NA	0.449
46	FCGR2A	rs4285675	1	161517585	NA	NA	NA	NA	NA
47	TGF-bR2	rs1350782	3	30662955	0.1654	0.244	0.2762	0.3803	0.8286
48	TGF-bR2	rs60793420	3	30630765	0.0757	0.7249	0.7373	0.06248	0.6136
49	MMP3	rs520540	11	102838694	0.69	0.7741	0.8331	0.1298	0.4901
50	ITPR3	rs2296336	6	33668883	0.7052	0.8232	0.5985	0.5846	0.6315
51	MMP3	rs678815	11	102843046	0.4854	0.5638	0.6591	0.1251	0.6804
52	TGF-bR2	rs3773646	3	30673265	0.3626	0.5421	0.4697	0.8107	NA
53	HMGB1	rs12428576	13	30498310	0.05741	0.8583	0.024	0.2139	0.06355
54	KCNN2	rs1380478	5	114469665	0.787	0.4508	0.4312	0.4003	0.9488

55	ITPR3	rs11755817	6	33642654	0.29	0.9801	0.9141	NA		0.3237
56	MMP3	rs591058	11	102840607	0.5818	0.5638	0.6591	0.1513		0.6022
57	KCNN2	rs11738819	5	114484755	0.2832	0.781	0.29	0.1847		0.7299
58	HMGB1	rs9579584	13	30498212	0.05076	0.8474	0.024	0.2139		0.06355
59	KCNN2	rs7710366	5	114444490	0.3549	0.514	0.3283	0.1014		0.1447
60	HMGB1	rs61947766	13	30498499	0.05259	0.9168	0.024	0.1403		0.06355
61	HMGB1	rs12583377	13	30498980	0.03893	0.7855	0.024	0.1586		0.06355
62	HMGB1	rs9579586	13	30499729	0.04842	0.8698	0.024	0.1586		0.06355
63	HMGB1	rs9579587	13	30501741	0.03895	0.7237	0.024	0.1586		0.07383
64	KCNN2	rs33975523	5	114449900	NA	NA	NA	NA		NA
65	TGF-bR2	rs79858912	3	30638502	0.1167	0.166	0.666	NA		NA
66	HMGB1	rs12431309	13	30497100	0.03735	0.819	0.024	0.1403		0.06355
67	KCNN2	rs181949	5	114372144	0.2533	0.2434	0.0839	0.6415		0.4086
68	BAZ1A|C14orf19	rs1200332	14	34,897,929	NA	NA				
69	GTSF1L|TOX2	rs6017164	20	43,753,466	0.7885	0.9099				
70	GPR6	rs4354185	6	109,980,458	0.4496	0.4256				
71	SLITRK5|MIRH1	rs7999399	13	88,581,250	0.05017	0.05396				
72	GPR6|WASF1	rs4317449	6	109,980,742	0.3416	0.3688				
73	REV3L	rs72945401	6	111,405,119	0.3355	0.1373				
74	CDH12	rs10473594	5	22,475,991	0.157	0.03669				
75	LOC728597|LOC727982	rs7579420	2	3,912,545	0.5158	0.01877				
76	GOLGA8B|GJD2	rs7163190	15	34,733,295	0.3654	0.6847				
77	NOTCH4|C6orf10	rs3134926	6	32,232,370	0.0587	0.5477				
78	TRAF3IP2	rs3777914	6	111,583,964	0.1718	0.0005896				
79	LARGE|ISX	rs16995211	22	35,055,646	0.5017	0.1573				
80	APBB2	rs719379	4	40,840,884	0.381	0.3883				
81	TRAF3IP2	rs1883137	6	111,585,943	0.234	0.0009908				
82	CDYL2	rs11641163	16	80,735,976	0.5453	0.8816				

*69-82 SNPs are top hits from our own family based study in Whites (TDT test)

Supplementary Table 6b SKAT analysis of genes previously associated with IVIG response

Run	Group	Name	Sub	Chr	St	En	Group	Gene	Sub	SKAT	SKATO	RareComm	RareCommO	Vars
223	1	TGFB2	E1I1	1	218345335	218363333	1	TGFB2	E1I1	0.92	0.74	0.88	0.63	278
1	1	TGFB2	ALL	1	218345336	218444619	1	TGFB2	ALL	0.89	0.55	0.96	0.43	1,655
224	1	TGFB2	I1E2	1	218347047	218363417	1	TGFB2	I1E2	0.92	0.77	0.93	0.71	256
225	1	TGFB2	E2I2	1	218363333	218405168	1	TGFB2	E2I2	0.89	0.51	0.87	0.37	746
226	1	TGFB2	I2E3	1	218363417	218405332	1	TGFB2	I2E3	0.88	0.54	0.85	0.41	738
227	1	TGFB2	E3I3	1	218405168	218434081	1	TGFB2	E3I3	0.57	0.76	0.75	0.54	469
228	1	TGFB2	I3E4	1	218405332	218434214	1	TGFB2	I3E4	0.59	0.68	0.77	0.47	468
229	1	TGFB2	E4I4	1	218434081	218434337	1	TGFB2	E4I4	0.42	0.40	0.47	0.33	2
230	1	TGFB2	I4E5	1	218434214	218434448								1
231	1	TGFB2	E5I5	1	218434337	218435969	1	TGFB2	E5I5	0.94	0.59	0.98	0.53	22
232	1	TGFB2	I5E6	1	218434448	218436147	1	TGFB2	I5E6	0.94	0.59	0.98	0.53	22
233	1	TGFB2	E6I6	1	218435969	218437342	1	TGFB2	E6I6	0.17	0.29	0.40	0.40	15
234	1	TGFB2	I6E7	1	218436147	218437496	1	TGFB2	I6E7	0.17	0.29	0.38	0.38	15
235	1	TGFB2	E7I7	1	218437342	218441203	1	TGFB2	E7I7	0.64	0.82	0.66	0.37	65
236	1	TGFB2	I7E8	1	218437496	218444619	1	TGFB2	I7E8	0.50	0.72	0.62	0.62	123
249	1	IL1B	E1I1	2	112829750	112831291	1	IL1B	E1I1	0.17	0.02	0.30	0.02	23
2	1	IL1B	ALL	2	112829751	112836779	1	IL1B	ALL	0.27	0.07	0.51	0.08	91
250	1	IL1B	I1E2	2	112830573	112831422	1	IL1B	I1E2	0.18	0.12	0.30	0.09	13
251	1	IL1B	E2I2	2	112831291	112832661								18
252	1	IL1B	I2E3	2	112831422	112832826	1	IL1B	I2E3	0.20	0.21	0.25	0.18	20
253	1	IL1B	E3I3	2	112832661	112833373	1	IL1B	E3I3	0.63	0.81	0.82	0.82	7
254	1	IL1B	I3E4	2	112832826	112833575	1	IL1B	I3E4	0.67	0.46	0.82	0.54	9
255	1	IL1B	E4I4	2	112833373	112835565	1	IL1B	E4I4	0.39	0.60	0.76	0.76	34
256	1	IL1B	I4E5	2	112833575	112835617	1	IL1B	I4E5	0.31	0.49	0.67	0.67	30
257	1	IL1B	E5I5	2	112835565	112836182	1	IL1B	E5I5	0.53	0.72	0.73	0.73	6
258	1	IL1B	I5E6	2	112835617	112836244	1	IL1B	I5E6	0.50	0.70	0.70	0.70	7
259	1	IL1B	E6I6	2	112836182	112836707	1	IL1B	E6I6	0.81	0.85	1.00	0.73	4
260	1	IL1B	I6E7	2	112836244	112836779	1	IL1B	I6E7	1.00	1.00	1.00	1.00	3
261	1	TGFBR2	E1I1	3	30606355	30644746	1	TGFBR2	E1I1	0.80	0.19	0.90	0.14	663
3	1	TGFBR2	ALL	3	30606356	30694142	1	TGFBR2	ALL	0.84	0.11	0.99	0.16	1,566
262	1	TGFBR2	I1E2	3	30606593	30644915	1	TGFBR2	I1E2	0.80	0.19	0.91	0.14	661
263	1	TGFBR2	E2I2	3	30644746	30650269	1	TGFBR2	E2I2	0.47	0.69	0.60	0.60	122
264	1	TGFBR2	I2E3	3	30644915	30650460	1	TGFBR2	I2E3	0.48	0.69	0.59	0.59	123
265	1	TGFBR2	E3I3	3	30650269	30671637	1	TGFBR2	E3I3	0.74	0.06	0.94	0.10	403
266	1	TGFBR2	I3E4	3	30650460	30672437	1	TGFBR2	I3E4	0.73	0.09	0.94	0.15	413
267	1	TGFBR2	E4I4	3	30671637	30674104	1	TGFBR2	E4I4	0.91	0.63	0.93	0.68	43
268	1	TGFBR2	I4E5	3	30672437	30674246	1	TGFBR2	I4E5	0.97	1.00	0.96	0.91	33
269	1	TGFBR2	E5I5	3	30674104	30688383	1	TGFBR2	E5I5	0.77	0.32	0.72	0.49	250
270	1	TGFBR2	I5E6	3	30674246	30688511	1	TGFBR2	I5E6	0.76	0.33	0.71	0.47	249
271	1	TGFBR2	E6I6	3	30688383	30691419	1	TGFBR2	E6I6	0.34	0.52	0.19	0.19	42
272	1	TGFBR2	I6E7	3	30688511	30694142	1	TGFBR2	I6E7	0.32	0.50	0.22	0.22	85
273	1	P2RY12	E1I1	3	151336842	151340595	1	P2RY12	E1I1	0.72	0.59	0.37	0.50	50
4	1	P2RY12	ALL	3	151336843	151384753	1	P2RY12	ALL	0.87	0.29	0.52	0.45	824
274	1	P2RY12	I1E2	3	151338859	151340760	1	P2RY12	I1E2	0.77	0.48	0.31	0.13	29
275	1	P2RY12	E2I2	3	151340595	151384691	1	P2RY12	E2I2	0.87	0.29	0.53	0.44	773
276	1	P2RY12	I2E3	3	151340760	151384753	1	P2RY12	I2E3	0.87	0.30	0.54	0.47	771
277	1	CASP3	E1I1	4	184627695	184631036	1	CASP3	E1I1	0.65	0.82	0.51	0.27	62
5	1	CASP3	ALL	4	184627696	184649447	1	CASP3	ALL	0.03	0.05	0.05	0.05	359
278	1	CASP3	I1E2	4	184629501	184631157	1	CASP3	I1E2	0.81	1.00	0.76	0.76	27
279	1	CASP3	E2I2	4	184631036	184631764	1	CASP3	E2I2	0.02	0.03	0.02	0.01	16
280	1	CASP3	I2E3	4	184631157	184631940	1	CASP3	I2E3	0.02	0.03	0.02	0.01	16
281	1	CASP3	E3I3	4	184631764	184632267	1	CASP3	E3I3	0.23	0.36	0.30	0.30	6
282	1	CASP3	I3E4	4	184631940	184632396	1	CASP3	I3E4	0.13	0.15	0.17	0.12	7
283	1	CASP3	E4I4	4	184632267	184635293	1	CASP3	E4I4	0.18	0.30	0.12	0.12	53
284	1	CASP3	I4E5	4	184632396	184635418	1	CASP3	I4E5	0.19	0.32	0.12	0.12	53

285	1	CASP3	E5I5	4	184635293	184638400	1	CASP3	E5I5	0.28	0.11	0.26	0.16	43
286	1	CASP3	I5E6	4	184635418	184638468	1	CASP3	I5E6	0.29	0.13	0.27	0.19	43
287	1	CASP3	E6I6	4	184638400	184648502	1	CASP3	E6I6	0.03	0.06	0.08	0.08	156
288	1	CASP3	I6E7	4	184638468	184648631	1	CASP3	I6E7	0.03	0.06	0.07	0.07	156
289	1	CASP3	E7I7	4	184648502	184649394	1	CASP3	E7I7	0.03	0.04	0.08	0.08	22
290	1	CASP3	I7E8	4	184648631	184649447	1	CASP3	I7E8	0.03	0.04	0.11	0.11	23
319	1	SMAD5	E1I1	5	136132844	136147831	1	SMAD5	E1I1	0.65	0.15	0.38	0.10	212
6	1	SMAD5	ALL	5	136132845	136182733	1	SMAD5	ALL	0.66	0.28	0.40	0.17	712
320	1	SMAD5	I1E2	5	136132962	136147906	1	SMAD5	I1E2	0.61	0.14	0.37	0.10	209
321	1	SMAD5	E2I2	5	136147831	136152674	1	SMAD5	E2I2	0.86	0.44	0.59	0.26	75
322	1	SMAD5	I2E3	5	136147906	136152758	1	SMAD5	I2E3	0.86	0.41	0.60	0.24	77
323	1	SMAD5	E3I3	5	136152674	136153591	1	SMAD5	E3I3	0.66	0.86	0.68	0.68	12
324	1	SMAD5	I3E4	5	136152758	136154163	1	SMAD5	I3E4	0.84	1.00	0.95	0.95	13
325	1	SMAD5	E4I4	5	136153591	136160855	1	SMAD5	E4I4	0.77	0.90	0.41	0.33	89
326	1	SMAD5	I4E5	5	136154163	136161107	1	SMAD5	I4E5	0.69	0.89	0.37	0.37	87
327	1	SMAD5	E5I5	5	136160855	136163271	1	SMAD5	E5I5	0.20	0.20	0.17	0.14	29
328	1	SMAD5	I5E6	5	136161107	136163391	1	SMAD5	I5E6	0.20	0.17	0.18	0.13	34
329	1	SMAD5	E6I6	5	136163271	136172433	1	SMAD5	E6I6	0.24	0.40	0.17	0.17	149
330	1	SMAD5	I6E7	5	136163391	136172655	1	SMAD5	I6E7	0.23	0.38	0.17	0.17	144
331	1	SMAD5	E7I7	5	136172433	136174375	1	SMAD5	E7I7	0.94	1.00	0.62	0.41	33
332	1	SMAD5	I7E8	5	136172655	136174632	1	SMAD5	I7E8	0.97	0.80	0.67	0.35	33
333	1	SMAD5	E8I8	5	136174375	136177336	1	SMAD5	E8I8	0.85	1.00	0.75	0.75	34
334	1	SMAD5	I8E9	5	136174632	136182733	1	SMAD5	I8E9	0.80	0.71	0.50	0.37	112
335	1	EXOC2	E1I1	6	485153	488978	1	EXOC2	E1I1	0.77	0.77	0.98	0.82	99
7	1	EXOC2	ALL	6	485154	693139	1	EXOC2	ALL	0.78	0.38	0.87	0.28	3,619
336	1	EXOC2	I1E2	6	486764	489038	1	EXOC2	I1E2	0.72	0.89	0.85	0.81	61
337	1	EXOC2	E2I2	6	488978	491124	1	EXOC2	E2I2	0.36	0.24	0.50	0.37	40
338	1	EXOC2	I2E3	6	489038	491186	1	EXOC2	I2E3	0.38	0.24	0.53	0.36	42
339	1	EXOC2	E3I3	6	491124	497366	1	EXOC2	E3I3	0.67	0.87	0.85	0.67	138
340	1	EXOC2	I3E4	6	491186	497489	1	EXOC2	I3E4	0.65	0.86	0.84	0.84	137
341	1	EXOC2	E4I4	6	497366	499644	1	EXOC2	E4I4	0.05	0.08	0.14	0.13	37
342	1	EXOC2	I4E5	6	497489	499700	1	EXOC2	I4E5	0.05	0.10	0.16	0.16	39
343	1	EXOC2	E5I5	6	499644	532468	1	EXOC2	E5I5	0.86	0.56	0.90	0.36	494
344	1	EXOC2	I5E6	6	499700	532610	1	EXOC2	I5E6	0.86	0.57	0.90	0.38	491
345	1	EXOC2	E6I6	6	532468	549174	1	EXOC2	E6I6	0.72	0.91	0.68	0.68	275
346	1	EXOC2	I6E7	6	532610	549291	1	EXOC2	I6E7	0.73	0.91	0.67	0.67	276
347	1	EXOC2	E7I7	6	549174	553853	1	EXOC2	E7I7	0.68	0.71	0.50	0.42	80
348	1	EXOC2	I7E8	6	549291	553920	1	EXOC2	I7E8	0.68	0.73	0.51	0.45	79
349	1	EXOC2	E8I8	6	553853	555226	1	EXOC2	E8I8	0.93	0.58	1.00	0.65	21
350	1	EXOC2	I8E9	6	553920	555288	1	EXOC2	I8E9	0.93	0.58	1.00	0.65	21
351	1	EXOC2	E9I9	6	555226	555953	1	EXOC2	E9I9	0.57	0.76	0.72	0.72	8
352	1	EXOC2	I9E10	6	555288	556013	1	EXOC2	I9E10	0.57	0.76	0.72	0.72	8
353	1	EXOC2	E10I10	6	555953	556483	1	EXOC2	E10I10	0.50	0.69	0.38	0.38	6
354	1	EXOC2	I10E11	6	556013	556564	1	EXOC2	I10E11	0.50	0.69	0.38	0.38	7
355	1	EXOC2	E11I11	6	556483	562783	1	EXOC2	E11I11	0.37	0.28	0.62	0.32	99
356	1	EXOC2	I11E12	6	556564	562845	1	EXOC2	I11E12	0.37	0.28	0.62	0.33	98
357	1	EXOC2	E12I12	6	562783	564032	1	EXOC2	E12I12	0.10	0.17	0.11	0.11	22
358	1	EXOC2	I12E13	6	562845	564154	1	EXOC2	I12E13	0.11	0.18	0.15	0.15	24
359	1	EXOC2	E13I13	6	564032	564544	1	EXOC2	E13I13	0.28	0.40	0.49	0.49	5
360	1	EXOC2	I13E14	6	564154	564702	1	EXOC2	I13E14	0.34	0.49	0.49	0.49	5
361	1	EXOC2	E14I14	6	564544	564863	1	EXOC2	E14I14	0.58	0.10	0.65	0.05	6
362	1	EXOC2	I14E15	6	564702	564929	1	EXOC2	I14E15	0.62	0.21	0.76	0.12	6
363	1	EXOC2	E15I15	6	564863	572519	1	EXOC2	E15I15	0.30	0.24	0.38	0.26	155
364	1	EXOC2	I15E16	6	564929	572644	1	EXOC2	I15E16	0.29	0.28	0.36	0.30	154
365	1	EXOC2	E16I16	6	572519	576756	1	EXOC2	E16I16	0.56	0.16	0.79	0.24	66
366	1	EXOC2	I16E17	6	572644	576882	1	EXOC2	I16E17	0.57	0.13	0.79	0.20	66
367	1	EXOC2	E17I17	6	576756	592468	1	EXOC2	E17I17	0.65	0.49	0.77	0.18	291
368	1	EXOC2	I17E18	6	576882	592587	1	EXOC2	I17E18	0.65	0.47	0.77	0.18	291

369	1	EXOC2	E18I18	6	592468	598020	1	EXOC2	E18I18	0.53	0.72	0.48	0.48	126
370	1	EXOC2	I18E19	6	592587	598123	1	EXOC2	I18E19	0.53	0.72	0.48	0.48	127
371	1	EXOC2	E19I19	6	598020	598859	1	EXOC2	E19I19	0.67	0.79	0.46	0.90	22
372	1	EXOC2	I19E20	6	598123	598941	1	EXOC2	I19E20	0.67	0.72	0.47	0.68	21
373	1	EXOC2	E20I20	6	598859	599079	1	EXOC2	E20I20	0.38	0.52	0.46	0.46	4
374	1	EXOC2	I20E21	6	598941	599225	1	EXOC2	I20E21	0.52	0.67	0.51	0.51	6
375	1	EXOC2	E21I21	6	599079	610097	1	EXOC2	E21I21	0.85	1.00	0.80	0.80	198
376	1	EXOC2	I21E22	6	599225	610178	1	EXOC2	I21E22	0.84	1.00	0.79	0.79	195
377	1	EXOC2	E22I22	6	610097	617710	1	EXOC2	E22I22	0.74	0.91	0.83	0.83	136
378	1	EXOC2	I22E23	6	610178	617835	1	EXOC2	I22E23	0.71	0.89	0.79	0.79	138
379	1	EXOC2	E23I23	6	617710	619429	1	EXOC2	E23I23	0.60	0.56	0.68	0.61	27
380	1	EXOC2	I23E24	6	617835	619543	1	EXOC2	I23E24	0.73	0.88	0.80	0.74	26
381	1	EXOC2	E24I24	6	619429	629834	1	EXOC2	E24I24	0.63	0.72	0.73	0.48	186
382	1	EXOC2	I24E25	6	619543	629961	1	EXOC2	I24E25	0.63	0.68	0.73	0.44	186
383	1	EXOC2	E25I25	6	629834	632940	1	EXOC2	E25I25	0.51	0.74	0.71	0.71	54
384	1	EXOC2	I25E26	6	629961	633117	1	EXOC2	I25E26	0.51	0.64	0.71	0.79	53
385	1	EXOC2	E26I26	6	632940	637700	1	EXOC2	E26I26	0.39	0.59	0.33	0.33	91
386	1	EXOC2	I26E27	6	633117	637861	1	EXOC2	I26E27	0.39	0.60	0.34	0.34	94
387	1	EXOC2	E27I27	6	637700	657162	1	EXOC2	E27I27	0.56	0.47	0.63	0.66	343
388	1	EXOC2	I27E28	6	637861	657250	1	EXOC2	I27E28	0.55	0.47	0.63	0.66	341
389	1	EXOC2	E28I28	6	657162	689138	1	EXOC2	E28I28	0.51	0.30	0.52	0.38	512
390	1	EXOC2	I28E29	6	657250	689212	1	EXOC2	I28E29	0.51	0.29	0.51	0.34	511
391	1	EXOC2	E29I29	6	689138	693018	1	EXOC2	E29I29	0.79	0.29	0.82	0.25	72
392	1	EXOC2	I29E30	6	689212	693139	1	EXOC2	I29E30	0.78	0.42	0.77	0.40	78
393	1	ADTRP	E1I1	6	11713522	11723348	1	ADTRP	E1I1	0.20	0.32	0.27	0.27	197
8	1	ADTRP	ALL	6	11713523	11778803	1	ADTRP	ALL	0.42	0.63	0.54	0.54	1,175
394	1	ADTRP	I1E2	6	11714512	11723500	1	ADTRP	I1E2	0.19	0.32	0.28	0.28	188
395	1	ADTRP	E2I2	6	11723348	11735567	1	ADTRP	E2I2	0.36	0.05	0.50	0.11	215
396	1	ADTRP	I2E3	6	11723500	11735683	1	ADTRP	I2E3	0.36	0.05	0.50	0.10	216
397	1	ADTRP	E3I3	6	11735567	11766273	1	ADTRP	E3I3	0.76	0.60	0.77	0.60	528
398	1	ADTRP	I3E4	6	11735683	11766375	1	ADTRP	I3E4	0.76	0.59	0.78	0.60	528
399	1	ADTRP	E4I4	6	11766273	11768248								33
400	1	ADTRP	I4E5	6	11766375	11768383								33
401	1	ADTRP	E5I5	6	11768248	11770030	1	ADTRP	E5I5	0.41	0.60	0.30	0.30	26
402	1	ADTRP	I5E6	6	11768383	11770084	1	ADTRP	I5E6	0.35	0.53	0.27	0.27	25
403	1	ADTRP	E6I6	6	11770030	11778606	1	ADTRP	E6I6	0.29	0.46	0.45	0.45	172
404	1	ADTRP	I6E7	6	11770084	11778803	1	ADTRP	I6E7	0.30	0.48	0.47	0.47	175
455	1	PLA2G7	E1I1	6	46704200	46705152	1	PLA2G7	E1I1	0.93	0.50	0.87	0.39	10
9	1	PLA2G7	ALL	6	46704201	46735721	1	PLA2G7	ALL	0.94	0.78	0.90	0.44	480
456	1	PLA2G7	I1E2	6	46704696	46705301	1	PLA2G7	I1E2	0.75	0.17	0.59	0.28	8
457	1	PLA2G7	E2I2	6	46705152	46707990	1	PLA2G7	E2I2	0.79	1.00	0.51	0.51	38
458	1	PLA2G7	I2E3	6	46705301	46708161	1	PLA2G7	I2E3	0.76	0.93	0.49	0.49	36
459	1	PLA2G7	E3I3	6	46707990	46709326	1	PLA2G7	E3I3	0.43	0.31	0.41	0.23	26
460	1	PLA2G7	I3E4	6	46708161	46709418	1	PLA2G7	I3E4	0.44	0.24	0.41	0.18	29
461	1	PLA2G7	E4I4	6	46709326	46710544	1	PLA2G7	E4I4	0.49	0.63	0.53	0.46	18
462	1	PLA2G7	I4E5	6	46709418	46710658	1	PLA2G7	I4E5	0.50	0.71	0.54	0.54	15
463	1	PLA2G7	E5I5	6	46710544	46711495	1	PLA2G7	E5I5	0.87	0.22	0.99	0.13	11
464	1	PLA2G7	I5E6	6	46710658	46711619	1	PLA2G7	I5E6	0.95	0.35	1.00	0.41	12
465	1	PLA2G7	E6I6	6	46711495	46712268	1	PLA2G7	E6I6	0.97	0.51	1.00	0.54	10
466	1	PLA2G7	I6E7	6	46711619	46712337	1	PLA2G7	I6E7	0.89	0.53	1.00	0.40	7
467	1	PLA2G7	E7I7	6	46712268	46714459	1	PLA2G7	E7I7	0.63	0.83	0.50	0.50	30
468	1	PLA2G7	I7E8	6	46712337	46714553	1	PLA2G7	I7E8	0.61	0.77	0.48	0.70	31
469	1	PLA2G7	E8I8	6	46714459	46716383	1	PLA2G7	E8I8	0.63	0.83	0.44	0.44	18
470	1	PLA2G7	I8E9	6	46714553	46716528	1	PLA2G7	I8E9	0.62	0.82	0.58	0.58	20
471	1	PLA2G7	E9I9	6	46716383	46716974	1	PLA2G7	E9I9	0.61	0.80	0.93	0.93	8
472	1	PLA2G7	I9E10	6	46716528	46717096	1	PLA2G7	I9E10	0.69	0.76	0.81	0.59	8
473	1	PLA2G7	E10I10	6	46716974	46722782	1	PLA2G7	E10I10	0.65	0.85	0.31	0.31	84
474	1	PLA2G7	I10E11	6	46717096	46722925	1	PLA2G7	I10E11	0.66	0.85	0.32	0.27	82

475	1	PLA2G7	E11I11	6	46722782	46735549	1	PLA2G7	E11I11	0.92	0.69	0.98	0.67	221
476	1	PLA2G7	I11E12	6	46722925	46735721	1	PLA2G7	I11E12	0.91	0.50	1.00	0.50	227
477	1	SAMD9L	E1I1	7	93130055	93144731	1	SAMD9L	E1I1	0.54	0.75	0.60	0.60	197
10	1	SAMD9L	ALL	7	93130056	93148385	1	SAMD9L	ALL	0.55	0.76	0.62	0.62	265
478	1	SAMD9L	I1E2	7	93135991	93144833	1	SAMD9L	I1E2	0.46	0.66	0.59	0.59	131
479	1	SAMD9L	E2I2	7	93144731	93145384	1	SAMD9L	E2I2	0.34	0.48	0.31	0.31	5
480	1	SAMD9L	I2E3	7	93144833	93145532	1	SAMD9L	I2E3	0.61	0.78	0.65	0.65	6
481	1	SAMD9L	E3I3	7	93145384	93145908	1	SAMD9L	E3I3	0.40	0.59	0.59	0.59	20
482	1	SAMD9L	I3E4	7	93145532	93146040	1	SAMD9L	I3E4	0.28	0.44	0.44	0.44	19
483	1	SAMD9L	E4I4	7	93145908	93146882	1	SAMD9L	E4I4	0.81	0.67	0.91	0.60	15
484	1	SAMD9L	I4E5	7	93146040	93147146	1	SAMD9L	I4E5	0.80	0.51	0.89	0.50	19
485	1	SAMD9L	E5I5	7	93146882	93148193	1	SAMD9L	E5I5	0.44	0.29	0.34	0.18	22
486	1	SAMD9L	I5E6	7	93147146	93148385	1	SAMD9L	I5E6	0.34	0.54	0.28	0.28	24
503	1	KLF6	E1I1	10	3775995	3780105	1	KLF6	E1I1	0.26	0.32	0.13	0.15	72
11	1	KLF6	ALL	10	3775996	3785209	1	KLF6	ALL	0.22	0.37	0.14	0.14	169
504	1	KLF6	I1E2	10	3779590	3780229	1	KLF6	I1E2	0.25	0.38	0.26	0.26	7
505	1	KLF6	E2I2	10	3780105	3781766	1	KLF6	E2I2	0.16	0.28	0.32	0.32	31
506	1	KLF6	I2E3	10	3780229	3782214	1	KLF6	I2E3	0.22	0.37	0.42	0.42	43
507	1	KLF6	E3I3	10	3781766	3784912	1	KLF6	E3I3	0.26	0.43	0.07	0.07	57
508	1	KLF6	I3E4	10	3782214	3785209	1	KLF6	I3E4	0.36	0.58	0.10	0.10	53
571	1	MMP12	E1I1	11	102862735	102864145	1	MMP12	E1I1	0.69	0.39	0.48	0.34	21
12	1	MMP12	ALL	11	102862736	102874982	1	MMP12	ALL	0.57	0.80	0.63	0.63	182
572	1	MMP12	I1E2	11	102863200	102864252	1	MMP12	I1E2	0.72	0.82	0.68	0.66	17
573	1	MMP12	E2I2	11	102864145	102865775	1	MMP12	E2I2	0.35	0.51	0.29	0.47	34
574	1	MMP12	I2E3	11	102864252	102865935	1	MMP12	I2E3	0.30	0.37	0.25	0.38	36
575	1	MMP12	E3I3	11	102865775	102866314	1	MMP12	E3I3	0.57	0.25	0.33	0.21	8
576	1	MMP12	I3E4	11	102865935	102866448	1	MMP12	I3E4	0.92	0.79	0.94	0.64	8
577	1	MMP12	E4I4	11	102866314	102867269	1	MMP12	E4I4	0.34	0.19	0.47	0.30	16
578	1	MMP12	I4E5	11	102866448	102867393	1	MMP12	I4E5	0.27	0.13	0.35	0.19	16
579	1	MMP12	E5I5	11	102867269	102867907	1	MMP12	E5I5	0.13	0.15	0.23	0.20	7
580	1	MMP12	I5E6	11	102867393	102868069								19
581	1	MMP12	E6I6	11	102867907	102871593	1	MMP12	E6I6	0.34	0.54	0.47	0.47	52
582	1	MMP12	I6E7	11	102868069	102871719	1	MMP12	I6E7	0.39	0.60	0.53	0.53	39
583	1	MMP12	E7I7	11	102871593	102871803	1	MMP12	E7I7	1.00	1.00	1.00	1.00	2
584	1	MMP12	I7E8	11	102871719	102871952	1	MMP12	I7E8	0.59	0.55	0.89	0.61	5
585	1	MMP12	E8I8	11	102871803	102872864	1	MMP12	E8I8	0.40	0.06	0.37	0.05	23
586	1	MMP12	I8E9	11	102871952	102873112	1	MMP12	I8E9	0.50	0.35	0.46	0.29	23
587	1	MMP12	E9I9	11	102872864	102874835	1	MMP12	E9I9	0.71	0.89	0.96	0.96	19
588	1	MMP12	I9E10	11	102873112	102874982	1	MMP12	I9E10	0.70	0.71	0.91	0.80	16
589	1	HMGB1	E1I1	13	30456703	30462537	1	HMGB1	E1I1	0.49	0.70	0.75	0.75	120
13	1	HMGB1	ALL	13	30456704	30617597	1	HMGB1	ALL	0.29	0.19	0.27	0.24	2,780
590	1	HMGB1	I1E2	13	30461533	30462712	1	HMGB1	I1E2	0.61	0.61	0.81	0.72	25
591	1	HMGB1	E2I2	13	30462537	30463206	1	HMGB1	E2I2	0.53	0.39	0.52	0.21	8
592	1	HMGB1	I2E3	13	30462712	30463352	1	HMGB1	I2E3	0.42	0.32	0.36	0.22	12
593	1	HMGB1	E3I3	13	30463206	30463530	1	HMGB1	E3I3	0.58	0.43	0.37	0.27	8
594	1	HMGB1	I3E4	13	30463352	30463694								1
595	1	HMGB1	E4I4	13	30463530	30617215	1	HMGB1	E4I4	0.27	0.18	0.25	0.23	2,638
596	1	HMGB1	I4E5	13	30463694	30617587	1	HMGB1	I4E5	0.27	0.17	0.25	0.23	2,644
597	1	ABCC4	E1I1	13	95019834	95034604	1	ABCC4	E1I1	0.70	0.53	0.65	0.21	266
14	1	ABCC4	ALL	13	95019835	95301451	1	ABCC4	ALL	0.36	0.01	0.22	0.02	5,098
598	1	ABCC4	I1E2	13	95021682	95034739	1	ABCC4	I1E2	0.70	0.41	0.60	0.17	226
599	1	ABCC4	E2I2	13	95034604	95043681	1	ABCC4	E2I2	0.35	0.33	0.18	0.34	124
600	1	ABCC4	I2E3	13	95034739	95043787	1	ABCC4	I2E3	0.35	0.35	0.18	0.36	121
601	1	ABCC4	E3I3	13	95043681	95044265	1	ABCC4	E3I3	0.77	1.00	0.49	0.49	10
602	1	ABCC4	I3E4	13	95043787	95044438	1	ABCC4	I3E4	0.91	1.00	0.55	0.55	12
603	1	ABCC4	E4I4	13	95044265	95053094	1	ABCC4	E4I4	0.15	0.22	0.39	0.56	150
604	1	ABCC4	I4E5	13	95044438	95053184	1	ABCC4	I4E5	0.13	0.17	0.37	0.52	152
605	1	ABCC4	E5I5	13	95053094	95062703	1	ABCC4	E5I5	0.26	0.02	0.21	0.00	184

606	1	ABCC4	I5E6	13	95053184	95062859	1	ABCC4	I5E6	0.23	0.02	0.18	0.00	183
607	1	ABCC4	E6I6	13	95062703	95071661	1	ABCC4	E6I6	0.36	0.56	0.10	0.10	171
608	1	ABCC4	I6E7	13	95062859	95071853	1	ABCC4	I6E7	0.39	0.60	0.12	0.12	168
609	1	ABCC4	E7I7	13	95071661	95073203	1	ABCC4	E7I7	0.27	0.43	0.17	0.17	26
610	1	ABCC4	I7E8	13	95071853	95073304	1	ABCC4	I7E8	0.27	0.43	0.17	0.17	26
611	1	ABCC4	E8I8	13	95073203	95074213	1	ABCC4	E8I8	0.41	0.61	0.62	0.62	12
612	1	ABCC4	I8E9	13	95073304	95074324	1	ABCC4	I8E9	0.20	0.31	0.38	0.38	13
613	1	ABCC4	E9I9	13	95074213	95075431	1	ABCC4	E9I9	0.28	0.44	0.10	0.10	17
614	1	ABCC4	I9E10	13	95074324	95075551	1	ABCC4	I9E10	0.35	0.53	0.12	0.12	18
615	1	ABCC4	E10I10	13	95075431	95083139	1	ABCC4	E10I10	0.48	0.68	0.32	0.32	165
616	1	ABCC4	I10E11	13	95075551	95083290	1	ABCC4	I10E11	0.48	0.69	0.33	0.33	166
617	1	ABCC4	E11I11	13	95083139	95115921	1	ABCC4	E11I11	0.05	0.09	0.02	0.01	512
618	1	ABCC4	I11E12	13	95083290	95116001	1	ABCC4	I11E12	0.06	0.10	0.02	0.01	509
619	1	ABCC4	E12I12	13	95115921	95161188	1	ABCC4	E12I12	0.32	0.05	0.04	0.01	776
620	1	ABCC4	I12E13	13	95116001	95161335	1	ABCC4	I12E13	0.32	0.05	0.04	0.01	777
621	1	ABCC4	E13I13	13	95161188	95163121	1	ABCC4	E13I13	0.74	0.31	0.79	0.20	23
622	1	ABCC4	I13E14	13	95161335	95163216	1	ABCC4	I13E14	0.83	0.34	0.95	0.24	24
623	1	ABCC4	E14I14	13	95163121	95163609	1	ABCC4	E14I14	0.52	0.23	0.31	0.15	10
624	1	ABCC4	I14E15	13	95163216	95163647	1	ABCC4	I14E15	0.32	0.23	0.20	0.13	8
625	1	ABCC4	E15I15	13	95163609	95164377	1	ABCC4	E15I15	0.32	0.08	0.19	0.04	19
626	1	ABCC4	I15E16	13	95163647	95164518	1	ABCC4	I15E16	0.47	0.15	0.27	0.09	23
627	1	ABCC4	E16I16	13	95164377	95166157	1	ABCC4	E16I16	0.88	1.00	0.33	0.33	25
628	1	ABCC4	I16E17	13	95164518	95166367	1	ABCC4	I16E17	0.78	1.00	0.26	0.26	23
629	1	ABCC4	E17I17	13	95166157	95170531	1	ABCC4	E17I17	0.92	1.00	0.97	0.69	82
630	1	ABCC4	I17E18	13	95166367	95170628	1	ABCC4	I17E18	0.93	1.00	0.98	0.98	80
631	1	ABCC4	E18I18	13	95170531	95177706	1	ABCC4	E18I18	0.50	0.73	0.40	0.40	146
632	1	ABCC4	I18E19	13	95170628	95177793	1	ABCC4	I18E19	0.50	0.74	0.41	0.41	148
633	1	ABCC4	E19I19	13	95177706	95177996	1	ABCC4	E19I19	0.62	0.33	0.87	0.25	6
634	1	ABCC4	I19E20	13	95177793	95178091	1	ABCC4	I19E20	0.51	0.39	0.83	0.41	4
635	1	ABCC4	E20I20	13	95177996	95186700	1	ABCC4	E20I20	0.35	0.54	0.51	0.51	153
636	1	ABCC4	I20E21	13	95178091	95186892	1	ABCC4	I20E21	0.34	0.53	0.51	0.51	155
637	1	ABCC4	E21I21	13	95186700	95188452	1	ABCC4	E21I21	0.43	0.07	0.73	0.18	36
638	1	ABCC4	I21E22	13	95186892	95188542	1	ABCC4	I21E22	0.53	0.04	0.79	0.11	37
639	1	ABCC4	E22I22	13	95188452	95194835	1	ABCC4	E22I22	0.60	0.17	0.82	0.33	103
640	1	ABCC4	I22E23	13	95188542	95194937	1	ABCC4	I22E23	0.60	0.22	0.82	0.42	102
641	1	ABCC4	E23I23	13	95194835	95206531	1	ABCC4	E23I23	0.95	0.08	0.99	0.18	206
642	1	ABCC4	I23E24	13	95194937	95206781	1	ABCC4	I23E24	0.95	0.05	0.98	0.13	210
643	1	ABCC4	E24I24	13	95206531	95207799	1	ABCC4	E24I24	0.26	0.42	0.44	0.44	27
644	1	ABCC4	I24E25	13	95206781	95207925	1	ABCC4	I24E25	0.26	0.42	0.48	0.48	22
645	1	ABCC4	E25I25	13	95207799	95209433	1	ABCC4	E25I25	0.62	0.84	0.61	0.61	35
646	1	ABCC4	I25E26	13	95207925	95209597	1	ABCC4	I25E26	0.62	0.84	0.59	0.59	41
647	1	ABCC4	E26I26	13	95209433	95210691	1	ABCC4	E26I26	0.16	0.27	0.27	0.27	33
648	1	ABCC4	I26E27	13	95209597	95210781	1	ABCC4	I26E27	0.33	0.51	0.34	0.34	33
649	1	ABCC4	E27I27	13	95210691	95234609	1	ABCC4	E27I27	0.32	0.12	0.44	0.26	483
650	1	ABCC4	I27E28	13	95210781	95234834	1	ABCC4	I27E28	0.31	0.12	0.44	0.27	480
651	1	ABCC4	E28I28	13	95234609	95246974	1	ABCC4	E28I28	0.22	0.05	0.34	0.25	235
652	1	ABCC4	I28E29	13	95234834	95247095	1	ABCC4	I28E29	0.22	0.06	0.33	0.28	235
653	1	ABCC4	E29I29	13	95246974	95247642	1	ABCC4	E29I29	0.17	0.27	0.20	0.20	15
654	1	ABCC4	I29E30	13	95247095	95247753	1	ABCC4	I29E30	0.18	0.28	0.19	0.19	12
655	1	ABCC4	E30I30	13	95247642	95301240	1	ABCC4	E30I30	0.43	0.02	0.34	0.04	1,046
656	1	ABCC4	I30E31	13	95247753	95301451	1	ABCC4	I30E31	0.43	0.02	0.36	0.04	1,048
683	1	IL16	E1I1	15	81182711	81188352	1	IL16	E1I1	0.94	0.88	0.94	0.88	98
15	1	IL16	ALL	15	81182712	81314058	1	IL16	ALL	0.52	0.72	0.56	0.56	2,350
684	1	IL16	I1E2	15	81182896	81188403	1	IL16	I1E2	0.94	0.86	0.93	0.86	98
685	1	IL16	E2I2	15	81188352	81225298	1	IL16	E2I2	0.63	0.35	0.72	0.34	627
686	1	IL16	I2E3	15	81188403	81225711	1	IL16	I2E3	0.63	0.34	0.72	0.31	628
687	1	IL16	E3I3	15	81225298	81259771	1	IL16	E3I3	0.24	0.37	0.23	0.23	659
688	1	IL16	I3E4	15	81225711	81259880	1	IL16	I3E4	0.23	0.37	0.23	0.23	656

689	1	IL16	E4I4	15	81259771	81265658	1	IL16	E4I4	0.55	0.61	0.49	0.34	101
690	1	IL16	I4E5	15	81259880	81265801	1	IL16	I4E5	0.52	0.61	0.48	0.34	103
691	1	IL16	E5I5	15	81265658	81269537	1	IL16	E5I5	0.25	0.31	0.38	0.32	67
692	1	IL16	I5E6	15	81265801	81269648	1	IL16	I5E6	0.24	0.34	0.36	0.32	66
693	1	IL16	E6I6	15	81269537	81273089	1	IL16	E6I6	0.52	0.68	0.61	0.66	70
694	1	IL16	I6E7	15	81269648	81273204	1	IL16	I6E7	0.57	0.77	0.64	0.64	71
695	1	IL16	E7I7	15	81273089	81278816	1	IL16	E7I7	0.73	0.89	0.72	0.72	123
696	1	IL16	I7E8	15	81273204	81278890	1	IL16	I7E8	0.73	0.89	0.72	0.72	122
697	1	IL16	E8I8	15	81278816	81279557								13
698	1	IL16	I8E9	15	81278890	81279774								16
699	1	IL16	E9I9	15	81279557	81282638	1	IL16	E9I9	0.73	0.30	0.84	0.26	48
700	1	IL16	I9E10	15	81279774	81282756	1	IL16	I9E10	0.75	0.45	0.88	0.38	46
701	1	IL16	E10I10	15	81282638	81285697	1	IL16	E10I10	0.77	0.74	0.90	0.70	66
702	1	IL16	I10E11	15	81282756	81285830	1	IL16	I10E11	0.79	0.62	0.93	0.59	69
703	1	IL16	E11I11	15	81285697	81290452	1	IL16	E11I11	0.19	0.33	0.43	0.43	79
704	1	IL16	I11E12	15	81285830	81290540	1	IL16	I11E12	0.16	0.28	0.39	0.39	76
705	1	IL16	E12I12	15	81290452	81292555	1	IL16	E12I12	0.10	0.17	0.29	0.29	34
706	1	IL16	I12E13	15	81290540	81293037	1	IL16	I12E13	0.13	0.22	0.36	0.36	39
707	1	IL16	E13I13	15	81292555	81296927	1	IL16	E13I13	0.98	1.00	0.86	0.44	66
708	1	IL16	I13E14	15	81293037	81297078	1	IL16	I13E14	0.98	1.00	0.81	0.53	66
709	1	IL16	E14I14	15	81296927	81299379	1	IL16	E14I14	0.70	0.75	0.44	0.25	36
710	1	IL16	I14E15	15	81297078	81300475	1	IL16	I14E15	0.65	0.85	0.39	0.39	46
711	1	IL16	E15I15	15	81299379	81301343	1	IL16	E15I15	0.60	0.80	0.31	0.31	28
712	1	IL16	I15E16	15	81300475	81301512	1	IL16	I15E16	0.54	0.11	0.32	0.15	14
713	1	IL16	E16I16	15	81301343	81303548	1	IL16	E16I16	0.05	0.05	0.04	0.05	30
714	1	IL16	I16E17	15	81301512	81303650	1	IL16	I16E17	0.06	0.02	0.04	0.02	27
715	1	IL16	E17I17	15	81303548	81305907	1	IL16	E17I17	0.78	0.59	0.61	0.40	48
716	1	IL16	I17E18	15	81303650	81306166	1	IL16	I17E18	0.81	0.57	0.65	0.42	52
717	1	IL16	E18I18	15	81305907	81306419	1	IL16	E18I18	0.75	0.89	0.60	0.60	6
718	1	IL16	I18E19	15	81306166	81306545	1	IL16	I18E19	0.60	0.73	0.63	0.63	2
719	1	IL16	E19I19	15	81306419	81308604	1	IL16	E19I19	0.78	0.87	0.76	0.41	37
720	1	IL16	I19E20	15	81306545	81314058	1	IL16	I19E20	0.48	0.39	0.46	0.16	151
721	1	IL4R	E1I1	16	27313755	27330065	1	IL4R	E1I1	0.32	0.49	0.17	0.17	271
16	1	IL4R	ALL	16	27313756	27364778	1	IL4R	ALL	0.02	0.03	0.01	0.01	921
722	1	IL4R	I1E2	16	27313817	27330198	1	IL4R	I1E2	0.31	0.49	0.17	0.17	268
723	1	IL4R	E2I2	16	27330065	27340185	1	IL4R	E2I2	0.17	0.15	0.02	0.11	185
724	1	IL4R	I2E3	16	27330198	27340273	1	IL4R	I2E3	0.16	0.16	0.02	0.11	186
725	1	IL4R	E3I3	16	27340185	27342120	1	IL4R	E3I3	0.15	0.09	0.00	0.00	35
726	1	IL4R	I3E4	16	27340273	27342259	1	IL4R	I3E4	0.19	0.04	0.00	0.00	37
727	1	IL4R	E4I4	16	27342120	27344868	1	IL4R	E4I4	0.13	0.15	0.00	0.01	55
728	1	IL4R	I4E5	16	27342259	27345020	1	IL4R	I4E5	0.12	0.18	0.00	0.00	60
729	1	IL4R	E5I5	16	27344868	27346466	1	IL4R	E5I5	0.10	0.17	0.21	0.21	42
730	1	IL4R	I5E6	16	27345020	27346618	1	IL4R	I5E6	0.05	0.09	0.10	0.10	36
731	1	IL4R	E6I6	16	27346466	27352539	1	IL4R	E6I6	0.01	0.01	0.05	0.05	110
732	1	IL4R	I6E7	16	27346618	27352696	1	IL4R	I6E7	0.01	0.02	0.06	0.05	109
733	1	IL4R	E7I7	16	27352539	27355807	1	IL4R	E7I7	0.00	0.00	0.01	0.01	56
734	1	IL4R	I7E8	16	27352696	27355907	1	IL4R	I7E8	0.00	0.00	0.01	0.01	56
735	1	IL4R	E8I8	16	27355807	27358915	1	IL4R	E8I8	0.24	0.05	0.04	0.03	52
736	1	IL4R	I8E9	16	27355907	27358994	1	IL4R	I8E9	0.10	0.08	0.02	0.03	53
737	1	IL4R	E9I9	16	27358915	27360765	1	IL4R	E9I9	0.05	0.08	0.02	0.02	37
738	1	IL4R	I9E10	16	27358994	27360815	1	IL4R	I9E10	0.22	0.23	0.05	0.10	35
739	1	IL4R	E10I10	16	27360765	27362251	1	IL4R	E10I10	0.14	0.09	0.18	0.08	26
740	1	IL4R	I10E11	16	27360815	27364778	1	IL4R	I10E11	0.08	0.13	0.15	0.15	78
741	1	TNFSF14	E1I1	19	6661252	6667112	1	TNFSF14	E1I1	0.45	0.45	0.48	0.04	118
17	1	TNFSF14	ALL	19	6661253	6670588	1	TNFSF14	ALL	0.34	0.25	0.50	0.21	160
742	1	TNFSF14	I1E2	19	6665350	6667154	1	TNFSF14	I1E2	0.63	0.83	0.79	0.79	35
743	1	TNFSF14	E2I2	19	6667112	6667412								1
744	1	TNFSF14	I2E3	19	6667154	6667449								2

745	1	TNFSF14	E3I3	19	6667412	6669850	1	TNFSF14	E3I3	0.19	0.32	0.39	0.39	32
746	1	TNFSF14	I3E4	19	6667449	6670158	1	TNFSF14	I3E4	0.16	0.27	0.36	0.36	35
747	1	TNFSF14	E4I4	19	6669850	6670295	1	TNFSF14	E4I4	0.19	0.11	0.37	0.35	7
748	1	TNFSF14	I4E5	19	6670158	6670588	1	TNFSF14	I4E5	0.49	0.66	0.30	0.30	4
749	1	CD209	E1I1	19	7739992	7744106	1	CD209	E1I1	0.20	0.26	0.12	0.12	94
18	1	CD209	ALL	19	7739993	7747534	1	CD209	ALL	0.27	0.36	0.19	0.19	151
750	1	CD209	I1E2	19	7743240	7744219	1	CD209	I1E2	0.40	0.46	0.49	0.49	36
751	1	CD209	E2I2	19	7744106	7744940	1	CD209	E2I2	0.62	0.74	0.85	0.85	17
752	1	CD209	I2E3	19	7744219	7745092	1	CD209	I2E3	0.77	0.85	0.92	0.95	16
753	1	CD209	E3I3	19	7744940	7745793	1	CD209	E3I3	0.82	1.00	0.73	0.73	14
754	1	CD209	I3E4	19	7745092	7746087	1	CD209	I3E4	0.87	0.82	0.78	0.55	16
755	1	CD209	E4I4	19	7745793	7746459	1	CD209	E4I4	0.12	0.20	0.09	0.09	6
756	1	CD209	I4E5	19	7746087	7746531	1	CD209	I4E5	0.07	0.10	0.06	0.06	3
757	1	CD209	E5I5	19	7746459	7747305	1	CD209	E5I5	0.30	0.46	0.30	0.38	16
758	1	CD209	I5E6	19	7746531	7747365	1	CD209	I5E6	0.32	0.49	0.31	0.31	15
759	1	CD209	E6I6	19	7747305	7747465	1	CD209	E6I6	0.40	0.34	0.35	0.21	3
760	1	CD209	I6E7	19	7747365	7747534	1	CD209	I6E7	0.35	0.32	0.31	0.23	4
761	1	ITPKC	E1I1	19	40717111	40725339	1	ITPKC	E1I1	0.83	0.88	0.78	0.66	126
19	1	ITPKC	ALL	19	40717112	40740860	1	ITPKC	ALL	0.55	0.76	0.63	0.63	379
762	1	ITPKC	I1E2	19	40718290	40725439	1	ITPKC	I1E2	0.80	1.00	0.76	0.76	106
763	1	ITPKC	E2I2	19	40725339	40729201	1	ITPKC	E2I2	0.76	0.91	0.85	0.85	59
764	1	ITPKC	I2E3	19	40725439	40729415	1	ITPKC	I2E3	0.86	1.00	0.94	0.94	68
765	1	ITPKC	E3I3	19	40729201	40733159	1	ITPKC	E3I3	0.17	0.29	0.21	0.21	71
766	1	ITPKC	I3E4	19	40729415	40733364	1	ITPKC	I3E4	0.09	0.17	0.14	0.14	65
767	1	ITPKC	E4I4	19	40733159	40736985	1	ITPKC	E4I4	0.39	0.60	0.49	0.49	63
768	1	ITPKC	I4E5	19	40733364	40737087	1	ITPKC	I4E5	0.42	0.64	0.51	0.51	60
769	1	ITPKC	E5I5	19	40736985	40737697	1	ITPKC	E5I5	0.22	0.35	0.20	0.20	9
770	1	ITPKC	I5E6	19	40737087	40737769	1	ITPKC	I5E6	0.18	0.30	0.18	0.18	11
771	1	ITPKC	E6I6	19	40737697	40739356	1	ITPKC	E6I6	0.63	0.84	0.83	0.83	33
772	1	ITPKC	I6E7	19	40737769	40739579	1	ITPKC	I6E7	0.64	0.85	0.83	0.83	32
773	1	ITPKC	E7I7	19	40739356	40739949	1	ITPKC	E7I7	0.33	0.50	0.55	0.55	8
774	1	ITPKC	I7E8	19	40739579	40740860	1	ITPKC	I7E8	0.52	0.73	0.71	0.71	17
775	1	NFATC2	E1I1	20	51386962	51398642	1	NFATC2	E1I1	0.30	0.47	0.48	0.48	246
20	1	NFATC2	ALL	20	51386963	51562839	1	NFATC2	ALL	0.93	0.84	0.99	0.69	3,217
776	1	NFATC2	I1E2	20	51391451	51398730	1	NFATC2	I1E2	0.14	0.24	0.30	0.30	182
777	1	NFATC2	E2I2	20	51398642	51432066	1	NFATC2	E2I2	0.92	0.75	0.97	0.82	623
778	1	NFATC2	I2E3	20	51398730	51432756	1	NFATC2	I2E3	0.92	0.77	0.95	0.81	627
779	1	NFATC2	E3I3	20	51432066	51435187	1	NFATC2	E3I3	0.57	0.78	0.67	0.67	51
780	1	NFATC2	I3E4	20	51432756	51435314	1	NFATC2	I3E4	0.62	0.81	0.71	0.71	44
781	1	NFATC2	E4I4	20	51435187	51435705	1	NFATC2	E4I4	0.00	0.00	0.00	0.00	3
782	1	NFATC2	I4E5	20	51435314	51435761	1	NFATC2	I4E5	0.00	0.00	0.00	0.00	2
783	1	NFATC2	E5I5	20	51435705	51454547	1	NFATC2	E5I5	0.91	0.63	0.92	0.50	327
784	1	NFATC2	I5E6	20	51435761	51454688	1	NFATC2	I5E6	0.91	0.64	0.93	0.51	328
785	1	NFATC2	E6I6	20	51454547	51473979	1	NFATC2	E6I6	0.65	0.85	0.49	0.49	349
786	1	NFATC2	I6E7	20	51454688	51474152	1	NFATC2	I6E7	0.65	0.85	0.49	0.49	348
787	1	NFATC2	E7I7	20	51473979	51475457	1	NFATC2	E7I7	0.66	0.57	0.68	0.47	22
788	1	NFATC2	I7E8	20	51474152	51475660	1	NFATC2	I7E8	0.80	0.62	0.73	0.40	26
789	1	NFATC2	E8I8	20	51475457	51516783	1	NFATC2	E8I8	0.77	0.77	0.84	0.75	741
790	1	NFATC2	I8E9	20	51475660	51516955	1	NFATC2	I8E9	0.76	0.77	0.83	0.76	738
791	1	NFATC2	E9I9	20	51516783	51523080	1	NFATC2	E9I9	0.97	1.00	1.00	0.87	121
792	1	NFATC2	I9E10	20	51516955	51524110	1	NFATC2	I9E10	0.97	1.00	1.00	0.79	136
793	1	NFATC2	E10I10	20	51523080	51562559	1	NFATC2	E10I10	0.94	0.85	1.00	1.00	728
794	1	NFATC2	I10E11	20	51524110	51562839	1	NFATC2	I10E11	0.93	0.87	0.98	0.90	718
